# A higher-order quadratic NLS equation on the half-line

**DOI:** 10.1007/s00028-024-01034-w

**Published:** 2024-12-15

**Authors:** A. Alexandrou Himonas, Fangchi Yan

**Affiliations:** 1 Department of Mathematics, University of Notre Dame, Notre Dame, IN 46556 USA; 2 Department of Mathematics, Virginia Polytechnic Institute and State University, Blacksburg, VA 24061 USA

**Keywords:** Higher-order nonlinear Schrödinger equation, Quadratic nonlinearities, Initial-boundary value problem on the half-line, Fokas unified transform method, Well-posedness in Sobolev spaces, Linear and bilinear estimates in Bourgain spaces, Primary 35Q55, 35G31, 35G16, 37K10

## Abstract

The well-posedness of the initial-boundary value problem for higher-order quadratic nonlinear Schrödinger equations on the half-line is studied by utilizing the Fokas solution formula for the corresponding linear problem. Using this formula, linear estimates are derived in Bourgain spaces for initial data in spatial Sobolev spaces on the half-line and boundary data in temporal Sobolev spaces suggested by the time regularity of the linear initial value problem. Then, the needed bilinear estimates are derived and used for showing that the iteration map defined via the Fokas solution formula is a contraction in appropriate solution spaces. Finally, well-posedness is established for optimal Sobolev exponents in a way analogous to the case of the initial value problem on the whole line with solutions in classical Bourgain spaces.

## Introduction and results

The initial-boundary value problem (ibvp) for the nonlinear Schrödinger equation of order *m* and with quadratic nonlinearities *uu*, $u\bar{u}$ and $\bar{u} \bar{u}$, which we denote by QNLS*m*, on the half-line $x>0$ is given by

1.1a $$\begin{aligned}&iu_t + (-1)^{j+1}\partial _{x}^{m}u +N_k(u,\bar{u})=0,  &   \quad (x, t)\in \mathbb {R}^+ \times (0, T), \ T< 1, \end{aligned}$$

1.1b $$\begin{aligned}&u(x, 0) = u_0(x),  &   \quad x\in \mathbb {R}^+, \end{aligned}$$

1.1c $$\begin{aligned}&u(0,t) = g_0(t), \ldots , \partial _x^{j-1}u(0,t) = g_{j-1}(t),  &   \quad t\in (0,T), \end{aligned}$$

where

$$\begin{aligned} m=2j, \qquad j=1,2,3\ldots , \end{aligned}$$

and $N_k(u,\bar{u})$, $k=1,2,3$, denotes the three nonlinearities

1.2 $$\begin{aligned} N_1(u, \bar{u}) = uu, \qquad N_2(u, \bar{u}) = u\bar{u} \quad \text {and} \quad N_3(u, \bar{u}) = \bar{u}\bar{u}. \end{aligned}$$

In this paper we study the well-posedness of this problem with rough initial and boundary data. More precisely, for initial data in spatial Sobolev spaces $H^s(0,\infty )$ with $s>s_k$, where the critical exponents $s_k$ are given by

1.3 $$\begin{aligned} s_{1}(j) = s_{3}(j) = -j+\frac{1}{4} \quad \text {and} \quad s_{2}(j) = -\frac{1}{2}j+\frac{1}{4}, \end{aligned}$$

and boundary data in temporal Sobolev spaces that are suggested by the time regularity of the linear initial value problem, that is

1.4 $$\begin{aligned} g_\ell \in H_t^\frac{2s+m-1-2\ell }{2m}(0,T), \quad \ell =0,\ldots , j-1, \end{aligned}$$

we show that ibvp (1.1) is well-posed with solution residing in appropriate Bourgain spaces restricted to $\mathbb {R}^+\times (0, T_0)$ for some positive lifespan $T_0$. Our proof is based on the Fokas unified transform method (UTM), which is utilized to find a solution formula for the corresponding forced linear problem. Then, using this formula we derive linear estimates in Bourgain solution spaces which indicate the needed bilinear estimates for proving that the iteration map defined by the Fokas solution formula is a contraction. Thus, well-posedness of our ibvp on the half-line is established **in a way analogous to the case of the initial value problem** (ivp) on the whole line, which for the case $m=2$ was studied by Kenig, Ponce and Vega in [45]. There, the well-posedness of this ivp in $H^s(\mathbb {R})$ was proved for the exponents given by (1.3), that is $s>-3/4$ for the nonlinearities *uu*, $\bar{u} \bar{u}$, and $s>-1/4$ for the nonlinearity $u \bar{u}$. Also, for $m=2$ the well-posedness of QNLSm on the half-line was studied in [8]. These results in combination with the well-posedness result for a higher dispersion KdV equation on the half-line [34] provide the main motivation for our work here.

**The Fokas solution formula** Next, we introduce the solution formula of the forced linear problem, which forms the basis of our analysis of QNLSm ibvp (1.1). This problem reads as follows

1.5a $$\begin{aligned}&iu_{t}+(-1)^{j+1}\partial _x^{m}u = f(x,t), \quad  &   x\in \mathbb {R}^+, \,\, t\in (0,T), \end{aligned}$$

1.5b $$\begin{aligned}&u(x,0) = u_{0}(x), \quad  &   x\in \mathbb {R}^+, \end{aligned}$$

1.5c $$\begin{aligned}&u(0,t) = g_0(t), \ldots , \partial _x^{j-1}u(0,t) = g_{j-1}(t), \quad  &   t\in (0,T). \end{aligned}$$

Employing the Fokas unified transform method (UTM) [20, 22], we obtain the following solution formula for the forced linear problem (1.5) (see Sect. 8 for a detailed derivation)

1.6 $$\begin{aligned} u(x,t)&= S\big [u_0,g_0,\ldots ,g_{j-1};f\big ](x, t) \doteq \frac{1}{2\pi }\int _{-\infty }^\infty e^{i\xi x-i\xi ^mt} [{\widehat{u}}_0(\xi )-iF(\xi ,t)]d\xi \nonumber \\&\quad \ + \sum \limits _{p=1}^{j} \sum \limits _{n=1}^{j} C_{p,n} \int _{\partial D_{2p-1}^+}e^{i\xi x-i\xi ^mt}[\widehat{u}(\alpha _{p,n}\xi )-iF(\alpha _{p,n}\xi ,t)]d\xi \nonumber \\&\quad \ + \sum \limits _{p=1}^j \sum \limits _{\ell =0}^{j-1} C'_{p,\ell } \int _{\partial D_{2p-1}^+}e^{i\xi x-i\xi ^mt}\xi ^{m-\ell -1}\tilde{g}_{\ell }(\xi ^m,T)d\xi , \end{aligned}$$

where $C_{p,n}$ and $C'_{p,\ell }$ are constants and the rotation numbers $\alpha _{p,n}$ are given by

1.7 $$\begin{aligned} \alpha _{p,n} \doteq e^{i[m-2p+2n]\cdot \frac{\pi }{m}}, \quad p=1,2,\dots ,j \quad n=1,2,\dots , j. \end{aligned}$$

Also, $\widehat{u}_0(\xi )$ is the half-line Fourier transform defined by

1.8 $$\begin{aligned} \widehat{u}_0(\xi ) = \int _0^\infty e^{-ix\xi } u_0(x) dx,\quad \text {Im}\,\xi \le 0, \end{aligned}$$

and $F(\xi ,t)$ is a time transform on (0, *t*) of the half-line Fourier transform of the forcing *f*

1.9 $$\begin{aligned} F(\xi ,t) \doteq \int _0^t e^{i\xi ^m\tau }{\widehat{f}}(\xi ,\tau )d\tau = \int _0^t e^{i\xi ^m\tau }\int _0^\infty e^{-i\xi x}f(x,\tau )dxd\tau . \end{aligned}$$

Finally, ${\tilde{g}}_\ell (\xi ,T)$ is the temporal transform of the boundary data $g_\ell$ over the interval (0, *T*)

**Fig. 1:**
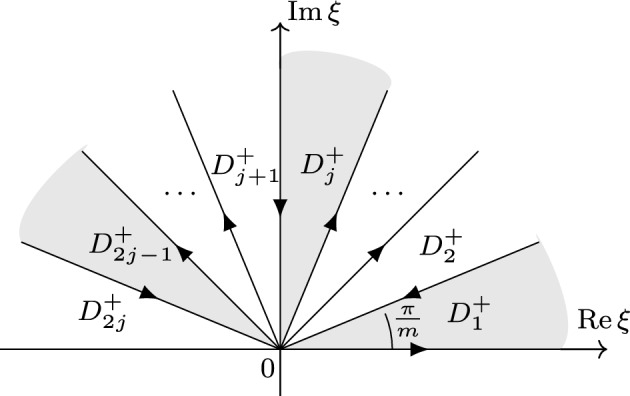
$$D^+$$ j is odd

**Fig. 2:**
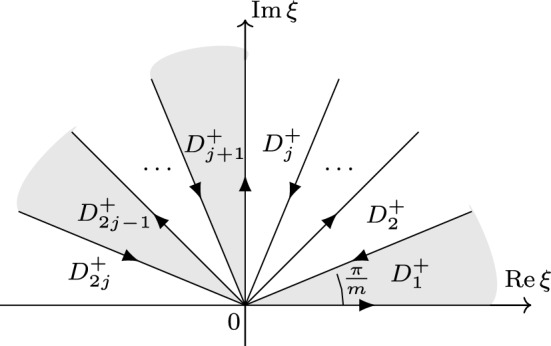
$$D^+$$ j is even

1.10 $$\begin{aligned} {\tilde{g}}_\ell (\xi ,T) \doteq \int _0^Te^{i\xi t}g_\ell (t)dt, \quad \xi \in \mathbb {C}, \end{aligned}$$

and $D_{2p-1}^+$ are the domains in the upper-half complex plain $\mathbb {C}^+$ that are sketched in Figs. 1 and 2.

**Linear estimates** Replacing in the UTM solution formula (1.6) the forcing term *f* with the nonlinearities $N_k(u,\bar{u})$, we obtain the iteration map

1.11 $$\begin{aligned} u \longmapsto S\big [u_0,g_0,\ldots ,g_{j-1};-N_k(u,\bar{u})\big ], \end{aligned}$$

for our nonlinear initial-boundary value problem (1.1). Like in the ivp case, for proving well-posedness of our nonlinear ibvp (1.1), the strategy (and our second step) is to estimate the UTM solution formula $S\big [u_0,g_0,\ldots ,g_{j-1};f\big ]$ in an appropriate Bourgain space restricted to $\mathbb {R}^+\times (0,T)$. Before stating our result of this estimation, we recall the needed spaces.

Following Bourgain [6, 7] and Kenig, Ponce, Vega [45, 46], we define the Bourgain space $X^{s,b}(\mathbb {R}^2)$ associated with the linear QNLSm equation via the norm

1.12 $$\begin{aligned} \Vert u\Vert _{X^{s,b}}^2 = \Vert u\Vert _{X^{s,b}(\mathbb {R}^2)}^2 \doteq \int _{\mathbb {R}^2} (1+ |\xi |)^{2s} (1+|\tau +\xi ^m|)^{2b} |\widehat{u}(\xi ,\tau )|^2 d\xi d\tau , \quad s,\, b \in \mathbb {R},\nonumber \\ \end{aligned}$$

where $\widehat{u}$ denotes the space-time Fourier transform

1.13 $$\begin{aligned} \widehat{u}(\xi ,\tau ) = \int _{\mathbb {R}^2} e^{-i(\xi x +\tau t)} u(x, t) dx dt. \end{aligned}$$

Also, we need the restriction of the space $X^{s,b}(\mathbb {R}^2)$ to the domain $\mathbb {R}^+\times (0, T)$, which is defined as follows

1.14 $$\begin{aligned} X_{\mathbb {R}^+\times (0, T)}^{s,b} \doteq \{ u: u(x,t) = \tilde{u}(x,t) \;\;\text{ on }\;\; \mathbb {R}^+\times (0,T) \,\, \text {with} \,\, \tilde{u}\in X^{s,b}(\mathbb {R}^2) \},\nonumber \\ \end{aligned}$$

and which is equipped with the norm

1.15 $$\begin{aligned} \Vert u\Vert _{X_{\mathbb {R}^+\times (0, T)}^{s,b}} \doteq \inf \limits _{\tilde{u}\in X^{s,b}}\left\{ \Vert \tilde{u}\Vert _{X^{s,b}}:\;\tilde{u}(x,t) = u(x,t) \;\;\text{ on }\;\; \mathbb {R}^+\times (0,T) \right\} . \end{aligned}$$

Moreover, when $s\not \in [-\frac{1}{2},\frac{1}{2})$, we shall need the temporal Bourgain spaces $Y^{s,b}$ defined by the norm

1.16 $$\begin{aligned} \Vert u\Vert _{Y^{s,b}}^2 \doteq \Vert u\Vert _{Y^{s,b}(\mathbb {R}^2)}^2 \doteq \int _{\mathbb {R}^2} (1+|\tau |)^{\frac{2}{m} s} (1+|\tau +\xi ^m|)^{2b} |\widehat{u}(\xi ,\tau )|^2 d\xi d\tau .\qquad \end{aligned}$$

These are motivated by [17, 18]. Also, for $m=2$, these temporal Bourgain spaces $Y^{s,b}$ were introduced in [8]. Their restriction spaces $Y_{\mathbb {R}^+\times (0, T)}^{s,b}$, are defined by

1.17 $$\begin{aligned} Y_{\mathbb {R}^+\times (0, T)}^{s,b} \doteq \{ u: u(x,t) = \tilde{u}(x,t) \;\;\text{ on }\;\; \mathbb {R}^+\times (0,T) \,\, \text {with} \,\, \tilde{u}\in Y^{s,b}(\mathbb {R}^2) \},\qquad \end{aligned}$$

and are equipped with the norm

1.18 $$\begin{aligned} \Vert u\Vert _{Y_{\mathbb {R}^+\times (0, T)}^{s,b}} \doteq \inf \limits _{\tilde{u}\in Y^{s,b}}\left\{ \Vert \tilde{u}\Vert _{Y^{s,b}}:\;\tilde{u}(x,t) = u(x,t) \;\;\text{ on }\;\; \mathbb {R}^+\times (0,T) \right\} . \end{aligned}$$

Finally, we need the following compatibility conditions for the initial and boundary data

1.19 $$\begin{aligned} \partial _x^\ell u_0(0) = g_\ell (0), \quad \text {for } \ell \,\, \text {and} \, s \text { such that } \, \frac{2s+m-1-2\ell }{2m}> \frac{1}{2} \ \left( \text {or } \, s>\ell +\frac{1}{2} \right) .\nonumber \\ \end{aligned}$$

Now, we can state the basic estimate in Bourgain spaces satisfied by the solution of the linear boundary-value problem on the half-line, which is analogous to the corresponding one in the case of the linear initial value problem on the whole line.

### Theorem 1.1

[Linear estimates] Suppose that $-\frac{1}{2}m-\frac{1}{2}<s<\frac{1}{2}m+\frac{1}{2}$, $s\ne \frac{1}{2},\frac{3}{2},\ldots ,j-\frac{1}{2}$ and $0<b<1/2$. Then, the Fokas formula (1.6) defines a solution $u\in X_{\mathbb {R}^+\times (0, T)}^{s,b}$ to the linear ibvp (1.5) with compatibility conditions (1.19), which satisfies the estimate

1.20 $$\begin{aligned}&\Vert S\big [u_0,g_0,\ldots ,g_{j-1};f\big ]\Vert _{X_{\mathbb {R}^+\times (0, T)}^{s,b}} \nonumber \\&\quad \le c_1 \left[ \Vert u_0\Vert _{H_x^{s}(0,\infty )} + \sum \limits _{\ell =0}^{j-1} \Vert g_\ell \Vert _{H^{\frac{2s+m-1-2\ell }{2m}}(0,T)} + \Vert f\Vert _{X_{\mathbb {R}^+\times (0, T)}^{s,-b}} + \Vert f\Vert _{Y_{\mathbb {R}^+\times (0, T)}^{s,-b}} \right] . \end{aligned}$$

Observe that for initial data in $H_x^{s}(0,\infty )$, the formula $\frac{2s+m-1-2\ell }{2m}$ that gives the exponent of the time regularity for the boundary data for the Schrödinger equation of even order *m* is the same as the one for linear KdV equation of odd order *m* studied in [34]. For example, for $m=2$ and $\ell =0$ we get the exponent $\frac{2s+1}{4}$ (Schrödinger equation) and for $m=3$ and $\ell =0$ we get the exponent $\frac{s+1}{3}$ (linear KdV equation).

The key ingredient for proving linear estimate (1.20) is the Fokas solution formula (1.6) which is first utilized in the simplest situation where forcing and initial data are zero while the boundary data are compactly supported. In this situation, the UTM solution formula is the simplest possible and using basic Fourier analysis we are able to bound its Bourgain norm by the Sobolev norms of the boundary data with correct time regularizes (see Theorem 2.1). Using this problem as the main tool we are able to decompose the full linear problem to simpler problems and estimate each one separately. Combining all estimates together gives linear estimate (1.20).

We mention that for $m=2$ and for smooth data, that is $1/2<s<3/2$, linear estimates for the Schrödinger equation ibvp in Hadamard spaces via the Fokas solution formula have been obtained in [24].

**Bilinear estimates** Applying the linear estimates (1.20) with *f* being replaced by the nonlinearities $-N_k(u,\bar{u})$, we see that the iteration map $u\mapsto S\big [u_0,g_0,\ldots ,g_{j-1};-N_k(u,\bar{u})\big ]$ becomes a contraction map in Bourgain spaces, if we can estimate the quantities $\Vert N_k(u,\bar{u}) \Vert _{X^{s,-b}}$ and $\Vert N_k(u,\bar{u}) \Vert _{Y^{s,-b}}$ appropriately. More precisely, we see that we need the following bilinear estimates, which we can show that are optimal.

### Theorem 1.2

(Bilinear estimates) $\bullet$  For some $0<b'\le b<\frac{1}{2}$, we have the **spatial** estimates

1.21 $$\begin{aligned}&\Vert N_k(f,\bar{g})\Vert _{X^{s,-b}} \le c_2 \Vert f\Vert _{X^{s,b'}} \Vert g\Vert _{X^{s,b'}}, \quad s>s_k, \end{aligned}$$

In fact, the bilinear estimates (1.21) holds for any $\frac{1}{2}-\tilde{\beta }_k\le b'\le b<\frac{1}{2},$ where

1.22 $$\begin{aligned}&\tilde{\beta }_k = \tilde{\beta }_k(s,m) = {\left\{ \begin{array}{ll} \frac{1}{6}, \quad &  s\ge 0, \\ \min \{\frac{1}{6}+\frac{s}{3m},\frac{1}{3}+\frac{4s-1}{6m}\}, \quad &  s_k<s<0, \end{array}\right. } \qquad \text {when} \,\, k=1,3.\end{aligned}$$

1.23 $$\begin{aligned}&\tilde{\beta }_2 = \tilde{\beta }_2(s,m) = {\left\{ \begin{array}{ll} \frac{1}{8}, \quad &  s\ge 0, \\ \min \{\frac{1}{6},\frac{1}{4}+\frac{1}{m}s-\frac{1}{4m}\}, \quad &  s_2<s<0. \end{array}\right. } \end{aligned}$$

$\bullet$  The spatial bilinear estimates (1.21) are optimal, i.e., they fail for $s< s_k$.

$\bullet$  In addition, we have the following bilinear estimates in **temporal** Bourgain spaces

1.24 $$\begin{aligned}&\Vert N_k(f,\bar{g})\Vert _{Y^{s,-b}} \le c_2 \Vert f\Vert _{X^{s,b'}} \Vert g\Vert _{X^{s,b'}}, \quad s_k<s<m-\frac{1}{2}, \end{aligned}$$

where $$ \frac{1}{2}-\beta _k\le b'\le b<\frac{1}{2}, $$ with

1.25 $$\begin{aligned} \beta _k = \beta _k(s,m) = \min \left\{ \frac{1}{2} \tilde{\beta }_k, \frac{1}{3}-\frac{2s+1}{6m} \right\} , \quad k=1,2,3. \end{aligned}$$

We mention that for the case $m=2$ and $1/2<b<1$ the spatial bilinear estimates (1.21) were proved in [45]. For $m=2$, $s_k<s\le 0$, and $b<\frac{1}{2}$, the bilinear estimates (1.21) and (1.24) were provided in [8] (see Lemma 7.1–Lemma 7.3). Also, we observe that the critical exponents for the symmetric nonlinearities *uu* and $\bar{u}\bar{u}$ are the same, that is $s_{1} = s_{3} = -j+\frac{1}{4},$ while the critical exponent for the asymmetric nonlinearity $u\bar{u}$ is different, that is $s_{2} = -\frac{1}{2}j+\frac{1}{4}.$ This is an interesting phenomenon considering that all these critical exponents are optimal. The symmetry/asymmetry of the nonlinearities $N_k, k=1,2,3$ is also reflected in their Bourgain quantities $d_{k, m}$ given by

1.26 $$\begin{aligned} d_{1,m}(\xi ,\xi _1) \doteq&\,\xi ^{m}-\xi _1^{m}-(\xi -\xi _1)^{m} = (\tau +\xi ^{m})-(\tau _1+\xi _1^{m})-[(\tau -\tau _1)+(\xi -\xi _1)^{m}], \end{aligned}$$

1.27 $$\begin{aligned} d_{2,m}(\xi ,\xi _1) \doteq&\,\xi ^{m}+\xi _1^{m}-(\xi -\xi _1)^{m} = (\tau +\xi ^{m})-(\tau _1-\xi _1^{m})-[(\tau -\tau _1)+(\xi -\xi _1)^{m}], \end{aligned}$$

1.28 $$\begin{aligned} d_{3,m}(\xi ,\xi _1) \doteq&\,\xi ^{m}+\xi _1^{m}+(\xi -\xi _1)^{m} = (\tau +\xi ^m)-(\tau _1-\xi _1^m)-[(\tau -\tau _1)-(\xi -\xi _1)^m]. \end{aligned}$$

These arise naturally in the $L^2$ formulation of the corresponding bilinear estimates from the multipliers appearing in the Bourgain norm of each term of the nonlinearity (see (3.9), (4.3) and (5.3)).

**Optimal well-posedness** Finally, we state our main local well-posedness result for the QNLSm ibvp considered in this work. This optimal result in the Bourgain spaces framework reads as follows.

### Theorem 1.3

(Well-posedness) Let $m=2j$, $j=1,2,\ldots$, $N_k(u,\bar{u})$, $k=1,2,3$, be the QNLSm nonlinearities defined in (1.2), and $s_k=s_k(m)$ be the corresponding critical exponents defined in (1.3). Then, for $s_k< s<\frac{1}{2}m+\frac{1}{2}$ and $s\ne \frac{1}{2},\frac{3}{2},\ldots ,j-\frac{1}{2}$, there exists $b\in (0,\frac{1}{2})$ depending on *s* such that for any initial data $u_0\in H_x^{s}(0,\infty )$ and boundary data $g_\ell \in H_t^\frac{2\,s+m-1-2\ell }{2\,m}(0,T)$, $\ell =0,1,2,\ldots ,j-1$, satisfying the compatibility conditions (1.19) there is a unique solution $u\in X_{\mathbb {R}^+\times (0, T_0)}^{s,b}$ to ibvp (1.1), which is the restriction to the domain $\mathbb {R}^+\times (0,T_0)$ of a function $\tilde{u}(x,t)$ in $X^{s,b}(\mathbb {R}^2)$ satisfying the size estimate

1.29 $$\begin{aligned} \Vert u\Vert _{X_{\mathbb {R}^+\times (0, T_0)}^{s,b}} \le c \left( \Vert u_0\Vert _{H^{s}(\mathbb {R}^+)} + \sum \limits _{\ell =0}^{j-1} \Vert g_\ell \Vert _{H^{\frac{2s+m-1-2\ell }{2m}}(0,T)} \right) , \end{aligned}$$

and having the following lifespan $T_0$ with $c_0 = c_0(s,b)$ and $\beta _k=\beta _k(s, m)>0$ defined in (1.25)

1.30 $$\begin{aligned} T_{0} = c_0 \left( 1+ \Vert u_0\Vert _{H^{s}(\mathbb {R}^+)} + \sum \limits _{\ell =0}^{j-1} \Vert g_\ell \Vert _{H^{\frac{2s+m-1-2\ell }{2m}}(0,T)} \right) ^{-2/\beta _k}. \end{aligned}$$

Furthermore, the solution depends Lip-continuously on the initial data $u_0$ and the boundary data $g_\ell$. Finally, this result is optimal in the framework of Bourgain spaces.

The proof of this result follows from applying the linear and bilinear estimates stated earlier to show that the iteration map defined from the Fokas solution formula by replacing the forcing with the nonlinearity is a contraction on a ball of an appropriate Bourgain solution space (see Sect. 7).

For $m=2$ the well-posedness of QNLSm in rough spaces was proved by [8] following the approach developed by Colliander and Kenig [12] and by Holmer [38, 39], which is based on expressing an ibvp as a superposition of initial value problems. Also, we mention that there is yet another approach for the study of the well-posedness for the NLS and KdV ibvp on the half-line, which is developed by Bona, Sun and Zhang [3–5] and which is based on using the Laplace transform in the temporal variable to solve the linear ibvp. A variation of this method has been used by Erdoǧan and N. Tzirakis for the study of the cubic NLS ibvp on the half line [15].

As we have mentioned our approach for the study of the well-posedness of QNLS ibvp (1.1) here as well as for our study of ibvp for other dispersive equations and systems, is based on the Fokas unified transform method, which was introduced in [20] and presented in great detail in monograph [22]. This method is motivated by the inverse scattering transform and provides a novel approach for solving initial-boundary value problems for linear and integrable nonlinear partial differential equations. In all of our work, it is the first and crucial step that provides the solution formula for the forced linear ibvp and opens the way for deriving good linear estimates analogues to those for initial value problems. For further results on the study of ibvp via the Fokas method we refer the reader to [1, 14, 20, 21, 23, 25–28, 30–33, 36, 37, 50, 51] and the references therein.

We conclude this introduction by stressing once more the analogy of methods used for studying the well-posedness of ivp and ibvp for dispersive equations, thanks to the Fokas unified transform method. And, this is true for smooth or rough data, and in one or higher dimensions.

Thus, the existing ivp theory plays an important role in the development of the corresponding ibvp theory.

Concerning the well-posedness of initial value problems of dispersive equations, like the KdV and NLS, there is an extensive literature. For some of these results, we refer the reader to [2, 6, 9–11, 13, 16, 40–44, 47–49, 52–55, 57] and the references therein.

**Structure** The paper is organized as follows. In Sect. 2, we derive the linear estimates. We begin with the so called reduced pure linear ibvp, which has forcing and initial data zero and compactly supported boundary data. Then, using this problem we decompose the forced linear ibvp into simpler problems and estimate each one separately to derive linear estimates (1.20). In Sect. 3, we derive the spatial bilinear estimates (1.21) for the symmetric nonlinearity $\bar{u}\bar{u}$, and prove their optimality. In Sect. 4, we derive the spatial bilinear estimates (1.21) for the other symmetric nonlinearity *uu*, and prove their optimality. In Sect. 5, we derive the spatial bilinear estimates (1.21) for the asymmetric nonlinearity $u\bar{u}$ and prove their optimality. In Sect. 6, we prove the temporal bilinear estimates (1.24). In Sect. 7, we outline the proof of our well-posedness result (Theorem 1.3) by using the linear and bilinear estimates. Finally, in Sect. 8, we derive the UTM solution formula for the forced linear ibvp.

**Notation** Here, for two quantities $Q_1$ and $Q_2$ depending on one or several variables, we write $Q_1 \lesssim Q_2$ if there is a constant $c>0$ such that $Q_1 \le c\, Q_2$. If $Q_1 \lesssim Q_2$ and $Q_2\lesssim Q_1$, then we write $Q_1\simeq Q_2$.

## Proof of linear estimates

In this section, we derive the linear estimates stated in Theorem 1.1. The key ingredient in this derivation is the Fokas solution formula (1.6) for the forced linear ibvp. Since estimating a Bourgain norm of this formula directly is difficult, we proceed by breaking the linear problem into simpler problems and estimating each one of them separately. The simplest and most important of all is what we call the reduced pure ibvp, which we consider next.

### Reduced pure ibvp

This problem is obtained from the full linear problem (1.5) by choosing forcing and initial data zero and nonzero boundary data. Furthermore, the boundary data are compactly supported in the interval [0, 2]. This problem is

2.1a $$\begin{aligned}&iv_t+(-1)^{j+1}\partial _x^mv=0,  &   (x,t) \in \mathbb {R}^+ \times (0,2), \end{aligned}$$

2.1b $$\begin{aligned}&v(x,0) = 0,  &   \end{aligned}$$

2.1c $$\begin{aligned}&v(0,t) = h_0(t), \ldots , \partial _{x}^{j-1}v(0,t) = h_{j-1}(t),  &   \,\,\, \text {supp}(h_\ell )\subset (0, 2), \,\, \ell =0, \ldots , j-1, \end{aligned}$$

and has the simplest Fokas solution formula. In fact, applying UTM formula (1.6) and using the support condition $\text {supp}(h_\ell )\subset (0, 2)$, which gives the relation

2.2 $$\begin{aligned} {\tilde{h}}_\ell (\xi ,2) = \int _0^2 e^{i\xi t}h_\ell (t)dt = \int _\mathbb {R}e^{i\xi t}h_\ell (t)dt = \widehat{h}_\ell (-\xi ), \end{aligned}$$

we get its solution in terms of the Fourier transform of the boundary data, that is

2.3 $$\begin{aligned} v(x,t)&= S\big [0,h_0,\ldots ,h_{j-1};0\big ](x, t) \nonumber \\&= \sum \limits _{p=1}^j \sum \limits _{\ell =0}^{j-1} C'_{p,\ell } \int _{\partial D_{2p-1}^+}e^{i\xi x-i\xi ^mt}\xi ^{m-\ell -1}\widehat{h}_{\ell }(-\xi ^m) d\xi = \sum \limits _{p=1}^j \sum \limits _{\ell =0}^{j-1} C'_{p,\ell } v_{p\ell }, \end{aligned}$$

where

2.4 $$\begin{aligned}  &   v_{p\ell }(x,t) \doteq \int _{\partial D_{2p-1}^+}e^{i\xi x-i\xi ^mt}\xi ^{m-\ell -1}{\widehat{h}}_{\ell }(-\xi ^m) d\xi , \nonumber \\  &   \quad p=1,2,\ldots , j, \,\, \ell =0,1,\ldots ,j-1. \end{aligned}$$

Next we estimate this solution in Bourgain spaces and obtain the following result.

#### Theorem 2.1

(Linear estimate for the reduced pure ibvp) For *m* an even positive integer the solution of the reduced pure ibvp (2.1) satisfies the Bourgain spaces estimate:

2.5 $$\begin{aligned} \Vert S\big [0,h_0,\ldots ,h_{j-1};0\big ] \Vert _{X^{s, b}_{\mathbb {R}^+\times (0,2)}}&\lesssim \sum \limits _{\ell =0}^{j-1} \Vert h_\ell \Vert _{H^{\frac{2s+m-1-2\ell }{2m}}(\mathbb {R})}, \nonumber \\ s&>-\frac{1}{2}m-\frac{1}{2}, \,\, 0\le b<\frac{1}{2}. \end{aligned}$$

Here and throughout this paper, we need the following **time localizer**:

2.6 $$\begin{aligned} \psi \in C^{\infty }_0(-1, 1), \,\,\, 0\le \psi \le 1 \,\, \text { and } \,\, \psi (t)=1 \,\, \text { for } \,\, |t|\le 1/2. \end{aligned}$$

#### Proof of Theorem 2.1

It suffices to prove inequality (2.5) for each component $v_{p\ell }$ defined in (2.4), that is for $s>-\frac{1}{2}m-\frac{1}{2}$ and $0\le b<\frac{1}{2}$ we have

2.7 $$\begin{aligned} \Vert v_{p\ell } \Vert _{X^{s, b}_{\mathbb {R}^+\times (0,2)}} \lesssim \Vert h_\ell \Vert _{H^{\frac{2s+m-1-2\ell }{2m}}(\mathbb {R})}, \quad p=1,2,\ldots , j, \,\, \ell =0,1,\ldots ,j-1.\nonumber \\ \end{aligned}$$

**Fig. 3:**
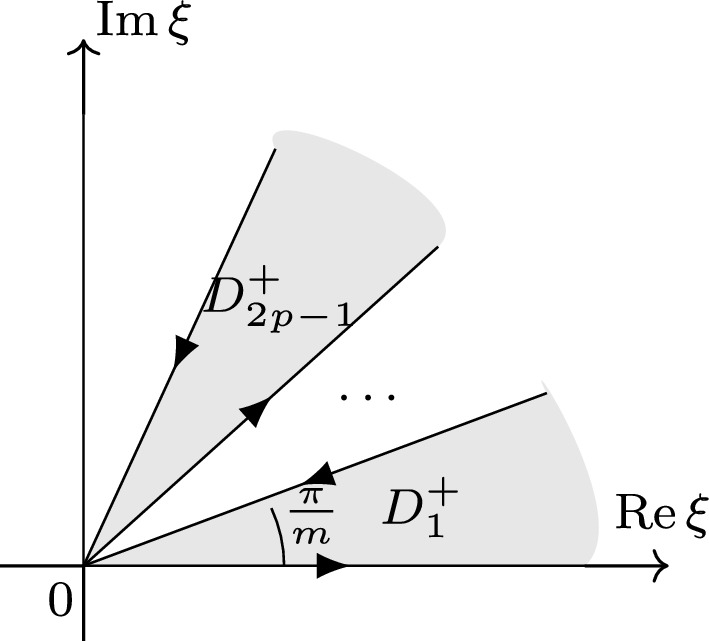
$$D_{2p-1}^+$$

We begin by parametrizing the boundary of the domain $D_{2p-1}^+$ (see Fig. 3) used in defining $v_{p\ell }$.

For the right side of the domain $D_{2p-1}^+$, we use the parametrization $[0,\infty )\ni \xi \rightarrow \gamma \xi$ and for the left side we use the parametrization $[0,\infty )\ni \xi \rightarrow \gamma '\xi$. Thus we write $v_{p\ell } = v_{l}+v_{r},$ where

2.8 $$\begin{aligned} v_{l}(x,t)&= \int _\infty ^0 e^{i\gamma ' \xi x+i\xi ^mt}(\gamma '\xi )^{m-\ell -1}{\widehat{h}}_{\ell }(\xi ^m) \gamma ' d\xi \nonumber \\&\simeq \int _0^\infty e^{i\xi ^mt}e^{i\gamma '\xi x} \xi ^{m-\ell -1} \, {\widehat{h}}_\ell (\xi ^m) d\xi , \end{aligned}$$

2.9 $$\begin{aligned} v_{r}(x,t)&= \int _0^\infty e^{i\gamma \xi x-i\xi ^mt}(\gamma \xi )^{m-\ell -1}{\widehat{h}}_{\ell }(-\xi ^m) \gamma d\xi \nonumber \\&\simeq \int _0^\infty e^{-i\xi ^mt}e^{i\gamma \xi x} \xi ^{m-\ell -1} \, {\widehat{h}}_\ell (-\xi ^m) d\xi , \end{aligned}$$

and

$$\begin{aligned}&\gamma = e^{i(2p-2)\cdot \frac{\pi }{m}} = \cos \left( \frac{2p-2}{m}\pi \right) +i\sin \left( \frac{2p-2}{m}\pi \right) , \,\,\, \\&\gamma ' = e^{i(2p-1)\cdot \frac{\pi }{m}} = \cos \left( \frac{2p-1}{m}\pi \right) +i\sin \left( \frac{2p-1}{m}\pi \right) . \end{aligned}$$

Since the estimation of $v_{r}$ and $v_l$ is similar, here we estimate only $v_{r}$. Also, we notice that when $p=1$, then $\gamma =1$, and the right side is the positive real line. However, when $p\ne 1$, then $\gamma$ is a complex number with positive imaginary part. Hence, we consider the following two cases $\bullet$ Case 1: $p=1$       $\bullet$ Case 2: $p\ne 1$.

**Proof in Case 1:** When $p=1$, then the integration in $v_r$ is over the positive real line, that is we have

2.10 $$\begin{aligned} v_{r}(x,t) = \int _0^\infty e^{i \xi x-i\xi ^mt}\xi ^{m-\ell -1}{\widehat{h}}_{\ell }(-\xi ^m)d\xi . \end{aligned}$$

Taking the Fourier transform with respect to *x*, we get

$$\begin{aligned} \widehat{v}_r^{x}(\xi ,t) = \chi _{\xi >0}(\xi ) \cdot e^{-i\xi ^mt}\xi ^{m-\ell -1}{\widehat{h}}_{\ell }(-\xi ^m). \end{aligned}$$

Hence, for $\psi _4(t)\doteq \psi (t/4)$, the full Fourier transform of $\psi _4 v_r$ is

$$\begin{aligned} \widehat{\psi _4v_r}(\xi ,\tau )&= \int _\mathbb {R}e^{-i\tau t} \psi _4(t) \cdot \chi _{\xi>0}(\xi ) \cdot e^{-i\xi ^mt}\xi ^{m-\ell -1}\widehat{h}_{\ell }(-\xi ^m) dt \\&= \chi _{\xi >0}(\xi ) \cdot \widehat{\psi }_4(\tau +\xi ^m) \xi ^{m-\ell -1}\widehat{h}_{\ell }(-\xi ^m). \end{aligned}$$

Now, by the definition of Bourgain norm, we get

2.11 $$\begin{aligned} \Vert \psi _4v_r\Vert _{X^{s,b}}^2 =&\int _{\mathbb {R}^2} (1+|\xi |)^{2s} (1+|\tau +\xi ^m|)^{2b} \Big | \chi _{\xi >0}(\xi )\nonumber \\&\cdot \widehat{\psi }_4(\tau +\xi ^m) \xi ^{m-\ell -1}{\widehat{h}}_{\ell }(-\xi ^m) \Big |^2 d\xi d\tau \nonumber \\ =&\, c_{\psi ,b} \int _{0}^\infty (1+|\xi |)^{2s} \xi ^{2m-2\ell -2} |{\widehat{h}}_{\ell }(-\xi ^m)|^2 d\xi , \end{aligned}$$

where $c_{\psi ,b} \doteq \int _{\mathbb {R}} (1+|\tau _1|)^{2b} |\widehat{\psi }_4(\tau _1)|^2 d\tau _1 = \Vert \psi _4\Vert _{H^b}^2.$ Next, making the change of variables $\tau =-\xi ^m$ or $\xi =(-\tau )^{\frac{1}{m}}$, from identity (2.11) we get

2.12 $$\begin{aligned} \Vert \psi _4v_r\Vert _{X^{s,b}}^2 \lesssim&\, \int _{-\infty }^0 (1+|\xi |)^{2s} \xi ^{2m-2\ell -2} |\widehat{h}_{\ell }(\tau )|^2 \cdot \frac{1}{|\partial _\xi \tau |} d\tau \nonumber \\ \lesssim&\, \int _{-\infty }^0 (1+|\tau |)^{\frac{2s}{m}} |\tau |^{\frac{m-2\ell -1}{m}} |{\widehat{h}}_{\ell }(\tau )|^2 d\tau . \end{aligned}$$

Furthermore, for $\ell \le j-1=\frac{1}{2}m-1$, we have $m-2\ell -1\ge m-2(\frac{1}{2}m-1)-1=1\ge 0$. This implies that $|\tau |^{\frac{m-2\ell -1}{m}} \le (1+|\tau |)^{\frac{m-2\ell -1}{m}}.$ Combining the above inequalities, we obtain

2.13 $$\begin{aligned} \Vert \psi _4v_r\Vert _{X^{s,b}}^2 \lesssim&\int _{-\infty }^0 (1+|\tau |)^{\frac{2s}{m}} (1+|\tau |)^{\frac{m-2\ell -1}{m}} |{\widehat{h}}_{\ell }(\tau )|^2 d\tau \le \Vert h_\ell \Vert _{H^{\frac{2s+m-1-2\ell }{2m}}}^2. \end{aligned}$$

This completes the proof of inequality (2.7) for $v_r(x,t)$ when $p=1$.

**Proof in Case 2:** When $p\ne 1$, i.e., the integration in $v_r$ is over a complex contour, then we split it for $\xi$ near 0 and for $\xi$ near $\infty$ and we write $v_r \simeq v_0 + v_1,$ where

2.14 $$\begin{aligned}&v_0(x,t) \doteq \int _0^1 e^{i\gamma \xi x-i\xi ^mt}\xi ^{m-\ell -1}{\widehat{h}}_{\ell }(-\xi ^m)d\xi , \quad x\in \mathbb {R}^+, \,\, t\in \mathbb {R}, \end{aligned}$$

2.15 $$\begin{aligned}&v_1(x,t) \doteq \int _1^\infty e^{i\gamma \xi x-i\xi ^mt}\xi ^{m-\ell -1}{\widehat{h}}_{\ell }(-\xi ^m)d\xi , \quad x\in \mathbb {R}^+, \,\, t\in \mathbb {R}. \end{aligned}$$

**Proof of inequality **(2.7) **for** $v_0$. Using a smooth version of |*x*|, like the function $\varphi _1(x)$ displayed

**Fig. 4:**
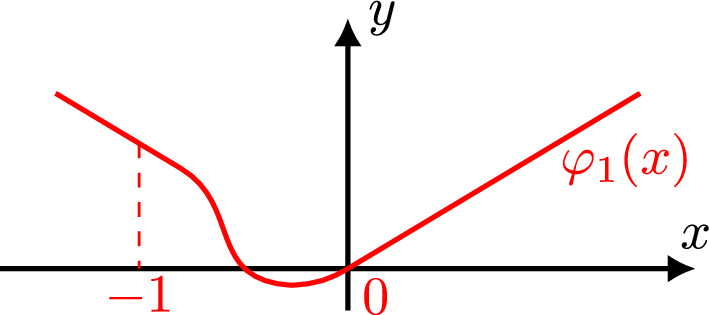
$$\varphi _1(x)$$

2.16 $$\begin{aligned} \varphi _1(x) = {\left\{ \begin{array}{ll} x, \quad \,\,\,\, x\ge 0, \\ -x, \quad x\le -1, \\ \text { smooth on } \mathbb {R}. \quad \end{array}\right. } \end{aligned}$$

in Fig. 4, we extend $v_0$ as an “almost” even function $V_0$ of *x* from $\mathbb {R}^+\times \mathbb {R}$ to $\mathbb {R}\times \mathbb {R}$ as follows

2.17 $$\begin{aligned} V_0(x,t) \doteq \int _0^1 e^{i\gamma \xi \varphi _1(x)-i\xi ^mt}\xi ^{m-\ell -1}{\widehat{h}}_{\ell }(-\xi ^m)d\xi \quad x\in \mathbb {R}, \,\, t\in \mathbb {R}. \end{aligned}$$

Then, using the bound $(1+|\xi |)^{2\,s} (1+|\tau +\xi ^m|)^{2b} \lesssim (|\xi |^0+|\xi |^{2mb+2|s|}) (|\tau |^0+|\tau |^{2b})$ for the Bourgain norm multipliers, we get the following $L^2$ estimate for $\psi _4(t)V_0$, with $\psi _4(t)=\psi (t/4)$,

2.18 $$\begin{aligned} \Vert \psi _4 V_0\Vert ^2_{X^{s,b}} \lesssim \Vert \psi _4 V_0\Vert ^2_{L^2_{x,t}} + \Vert \partial _x^{n_1}[\psi _4 V_0]\Vert ^2_{L^2_{x,t}} + \Vert \partial _t^{n_2}[\psi _4 V_0]\Vert ^2_{L^2_{x,t}} + \Vert \partial _x^{n_1}\partial _t^{n_2}[\psi _4 V_0]\Vert ^2_{L^2_{x,t}}, \end{aligned}$$

where $n_1 = n_1(s,b) \doteq 2\,m\lfloor b\rfloor +2\lfloor |s|\rfloor +2\,m+2$ and $n_2 = n_2(b) \doteq 2\lfloor b\rfloor +2.$ Furthermore, using appropriately the Laplace transform $L^2$ boundedness [24, 29], for any nonnegative integers $n_1,n_2$ we get

2.19 $$\begin{aligned} \Vert \partial _x^{n_1}\partial _t^{n_2}[\psi _4\,V_0]\Vert _{L^2_{x,t}}^2 \le C_{n_1, n_2} \int _{-1}^{0} | \widehat{h}_\ell (\tau ) |^2 d\tau . \end{aligned}$$

The proof of the above estimate is similar to that of estimate (2.15) in [35], and we omit it here. Combining (2.19) with inequality (2.18), we obtain the following bound for the extension $\psi _4 V_0$

$$\begin{aligned} \Vert \psi _4 V_0\Vert ^2_{X^{s,b}}&\lesssim \int _{-1}^{0} (1+|\tau |)^{-\frac{2s+m-1-2\ell }{m}} (1+|\tau |)^{\frac{2s+m-1-2\ell }{m}} | \widehat{h_\ell }(\tau ) |^2 d\tau \\&\lesssim \Vert h_\ell \Vert _{H^{\frac{2s+m-1-2\ell }{2m}}}^2, \,\, s\in \mathbb {R},\, b\ge 0, \end{aligned}$$

which gives the desired bound (2.7) for $v_0$.

**Fig. 5:**
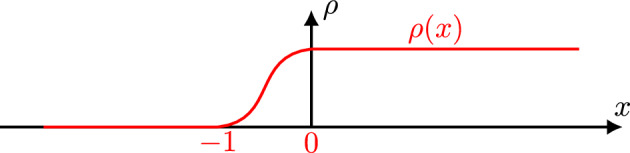
One sided cut-off $\rho (x)$

**Proof of inequality **(2.7) **for** $v_1$. By using $\gamma =e^{i(2p-2)\cdot \frac{\pi }{m}}=\gamma _R +i \gamma _I$, where $\gamma _I=\sin \left( \frac{2p-2}{m}\pi \right) >0$ ($p=2,3,\ldots ,j$), we rewrite $v_1$ as

2.20 $$\begin{aligned} v_1(x,t) = \int _1^\infty e^{-i\xi ^mt}e^{i\gamma _R \xi x}e^{-\gamma _I\xi x}\xi ^{m-\ell -1}{\widehat{h}}_{\ell }(-\xi ^m)d\xi , \quad x>0, \,\, t\in \mathbb {R}. \end{aligned}$$

Also, since $\frac{1}{2}m$ is a positive integer, using the familiar differentiation formula $\partial _x^{\frac{1}{2}m} [ e^{i\gamma \xi x} ] = (i\gamma \xi )^{\frac{1}{2}m} e^{i\gamma \xi x}$ and manipulating the exponentials, we obtain the elementary but very useful identity

$$\begin{aligned} \xi ^{\frac{1}{2}m} [ e^{i\gamma _R \xi x}e^{-\gamma _I\xi x} ] = \frac{\partial _x^{\frac{1}{2}m}}{(i\gamma )^{\frac{1}{2}m}} [ e^{i\gamma _R \xi x}e^{-\gamma _I\xi x} ], \end{aligned}$$

which combined with fact that $e^{-\gamma _I \xi x}$ decays exponentially in $\xi$ for $x>0$ (since $\gamma _I>0$), we move the $\partial _x^{\frac{1}{2}m}$ derivative outside the integral in (2.20) and write $v_1$ as follows

2.21 $$\begin{aligned} v_1(x,t)  &   = \frac{1}{(i\gamma )^{\frac{1}{2}m}} \partial _x^{\frac{1}{2}m} \int _1^\infty e^{-i\xi ^mt}e^{i\gamma _R \xi x}e^{-\gamma _I\xi x}\xi ^{\frac{1}{2}m-\ell -1}{\widehat{h}}_{\ell }(-\xi ^m)d\xi , \nonumber \\  &   \quad \ x>0, \,\, t\in \mathbb {R}. \end{aligned}$$

In order to estimate the $X^{s,b}_{\mathbb {R}^+\times (0,2)}$ norm of $v_1$, we need to extend it from $\mathbb {R}^+\times (0,2)$ to $\mathbb {R}^2$. To achieve this, we introduce the one-sided $C^\infty$ cut-off function $\rho (x)$ displayed in Fig. 5. It is supported in $(-1, \infty )$, is increasing in $(-1, 0)$, and $0\le \rho (x)\le 1$ for all $x\in \mathbb {R}$.

2.22 $$\begin{aligned} \rho (x) = {\left\{ \begin{array}{ll} 1, & \,\, x\ge 0, \\ 0, & \,\, x\le -1. \end{array}\right. } \end{aligned}$$

By using this cut-off function we extend $v_1$ from $\mathbb {R}^+\times \mathbb {R}$ to $V_1$ on $\mathbb {R}^2$ via the following formula

$$\begin{aligned} V_1(x,t) \doteq&\frac{1}{(i\gamma )^{\frac{1}{2}m}} \partial _x^{\frac{1}{2}m} \int _1^\infty \rho (\gamma _I\xi x) e^{-i\xi ^mt}e^{i\gamma _R \xi x}e^{-\gamma _I\xi x} \xi ^{\frac{1}{2}m-\ell -1}\widehat{h}_{\ell }(-\xi ^m)d\xi \\ \simeq&\partial _x^{\frac{1}{2}m} \int _1^\infty e^{-i\xi ^mt} \rho (\gamma _I\xi x) e^{i\gamma _R \xi x}e^{-\gamma _I\xi x} \xi ^{\frac{1}{2}m-\ell -1}{\widehat{h}}_{\ell }(-\xi ^m)d\xi , \quad x\in \mathbb {R}, \,\, t\in \mathbb {R}. \end{aligned}$$

Since $\Vert v_1\Vert _{X^{s,b}_{\mathbb {R}^+\times (0,2)}} \le \Vert V_1\Vert _{X^{s,b}},$ to establish the estimate (2.7) for $v_1$ it suffices to show that

2.23 $$\begin{aligned} \Vert V_1\Vert _{X^{s,b}} \lesssim \Vert h_\ell \Vert _{H_t^{\frac{2s+m-1-2\ell }{2m}}(\mathbb {R})}. \end{aligned}$$

For this we need to find the Fourier transform of $V_{1}$, which requires to express it in a convenient form. First, we make the change of variables $\tau =-\xi ^m$ or $\xi =|\tau |^{1/m}$ ($\tau <-1$) and write it as follows

2.24 $$\begin{aligned} V_1(x,t) \simeq \partial _x^{\frac{1}{2}m} \int _{-\infty }^{-1} e^{i\tau t} \rho (\gamma _I \xi x) e^{i\gamma _R \xi x}e^{-\gamma _I\xi x} |\xi |^{\frac{1}{2}m-\ell -1} \, {\widehat{h}}_\ell (\tau ) \frac{1}{|\partial _\xi \tau |} d\tau , \end{aligned}$$

where $|\partial _\xi \tau |\simeq |\xi |^{m-1}$. Then, we define the Schwartz function

2.25 $$\begin{aligned} \eta (x) \doteq \rho (x) e^{i\frac{\gamma _R}{\gamma _I}x} e^{-x}. \end{aligned}$$

Finally, using (2.25) and the fact that $\rho (\gamma _I\xi x)e^{i\gamma _R \xi x}e^{-\gamma _I\xi x} = \eta (\gamma _I|\tau |^{1/m}x),$ we can express $V_1$ as follows

2.26 $$\begin{aligned} V_1(x,t) \simeq \partial _x^{\frac{1}{2}m} \int _{-\infty }^{-1} e^{i\tau t} \eta (\gamma _I|\tau |^{1/m}x) \, |\tau |^{\frac{-m-2\ell }{2m}} \, {\widehat{h}}_\ell (\tau ) d\tau , \quad x\in \mathbb {R}, \,\, t\in \mathbb {R}. \end{aligned}$$

Now, using formula (2.26), we see that the Fourier transform of $V_1$ with respect to *t* is given by

$$\begin{aligned} \widehat{V}_{1}^t(x,\tau ) \simeq \partial _x^{\frac{1}{2}m} \big [ \chi _{(-\infty ,-1)}(\tau ) \cdot \eta (\gamma _I|\tau |^{1/m}x) \, |\tau |^{\frac{-m-2\ell }{2m}} \, {\widehat{h}}_\ell (\tau ) \big ], \end{aligned}$$

and therefore its full Fourier transform is

2.27 $$\begin{aligned} \widehat{V}_{1}(\xi ,\tau )&\simeq \xi ^{\frac{1}{2}m} \chi _{(-\infty ,-1)}(\tau ) \, F(\xi ,\tau ) \, |\tau |^{\frac{-m-2\ell }{2m}} \, {\widehat{h}}_\ell (\tau ), \,\,\,\, \text {with} \,\,\,\, F(\xi ,\tau ) \nonumber \\&\doteq \int _{\mathbb {R}} e^{-i\xi x}\eta (\gamma _I|\tau |^{1/m}x) dx. \end{aligned}$$

Using (2.27) we estimate $\Vert V_{1}\Vert _{X^{s,b}}^2$ as follows

2.28 $$\begin{aligned} \Vert V_{1}\Vert _{X^{s,b}}^2&\lesssim \int _{-\infty }^{-1} \Big [ \int _{-\infty }^{\infty } (1+|\xi |)^{2s} (1+|\tau +\xi ^m|)^{2b} \xi ^m | F(\xi ,\tau ) |^2 d\xi \Big ] \nonumber \\&\quad \ \cdot |\tau |^{\frac{-m-2\ell }{m}} \, | {\widehat{h}}_\ell (\tau ) |^2 d\tau . \end{aligned}$$

In addition, we can estimate the $d\xi$-integral by using the following result.

#### Lemma 2.1

Let $\varepsilon >0$ and $\tau <-1$. If $s> -\frac{m+1}{2}-\varepsilon$, $b\ge 0$, then we have

2.29 $$\begin{aligned} \int _{-\infty }^{\infty } (1+|\xi |)^{2s} (1+|\tau +\xi ^m|)^{2b} \xi ^m | F(\xi ,\tau ) |^2 d\xi \lesssim |\tau |^{\frac{2s+2mb+m-1+2\varepsilon }{m}}. \end{aligned}$$

Combining inequality (2.28) and Lemma 2.1, for $\varepsilon >0$, $s> -\frac{m+1}{2}-\varepsilon$ and $b\ge 0$ we obtain

2.30 $$\begin{aligned} \Vert V_{1}\Vert _{X^{s,b}}^2 \lesssim&\int _{-\infty }^{-1} |\tau |^{\frac{2s+2mb+m-1+2\varepsilon }{m}} |\tau |^{\frac{-m-2\ell }{m}} \, | {\widehat{h}}_\ell (\tau ) |^2 d\tau \lesssim \Vert h_\ell \Vert _{H^{\frac{2s+2mb-1-2\ell +2\varepsilon }{2m}}}^2. \end{aligned}$$

In (2.30), choosing $\varepsilon >0$ such that $\frac{2s+2mb-1-2\ell +2\varepsilon }{2m}\le \frac{2s+m-1-2\ell }{2m}$, or $2mb+2\varepsilon \le m$, or $\varepsilon \le \frac{m}{2}(1-2b)$, we obtain the desired inequality (2.23). Since $\varepsilon >0$, we must have $\frac{m}{2}(1-2b)>0$ or

2.31 $$\begin{aligned} \boxed { b<1/2. } \end{aligned}$$

This completes the proof of Theorem 2.1 once we prove Lemma 2.1. $\square$

#### Proof of Lemma 2.1

To prove this result, we need the following estimate for *F* defined in (2.27), which follows from the fact that $\eta$ is a Schwartz function. $\square$

#### Lemma 2.2

For any $n\ge 0$, $\tau <-1$, and $\xi \in \mathbb {R}$, we have

2.32 $$\begin{aligned} |F(\xi ,\tau )| \le c_{\rho ,\gamma , n} \, \frac{1}{|\tau |^{1/m}} \, \left( \frac{|\tau |^{1/m}}{|\xi |+|\tau |^{1/m}} \right) ^n, \end{aligned}$$

where $c_{\rho ,\gamma ,n}$ is a constant depending on $\rho$, $\gamma$ and *n*.

Using inequality (2.32) we bound the integrand $I\doteq (1+|\xi |)^{2\,s} (1+|\tau +\xi ^m|)^{2b} \xi ^m | F(\xi ,\tau ) |^2$ in (2.29) as follows

2.33 $$\begin{aligned} I(\xi ,\tau ) \lesssim (1+|\xi |)^{2s+m} (1+|\tau +\xi ^m|)^{2b} \frac{|\tau |^{\frac{2n-2}{m}}}{|\xi |^{2n}+|\tau |^{2n/m}}. \end{aligned}$$

Then, we consider the two cases: $|\xi |> |\tau |^{1/m}$ and $|\xi |\le |\tau |^{1/m}$. In the case $|\xi |> |\tau |^{1/m}$ ($\tau <-1$), from inequality (2.33) we have

2.34 $$\begin{aligned} I(\xi ,\tau ) \lesssim (1+|\xi |)^{2s+m}|\xi |^{2mb} \frac{|\tau |^{\frac{2n-2}{m}}}{|\xi |^{2n}}, \quad s\in \mathbb {R}, \,\, b\ge 0. \end{aligned}$$

Using inequality (2.34), choosing $n=s+mb+\frac{m+1}{2}+\varepsilon$ and integrating over $\xi$, we obtain the desired inequality (2.29). In the case $|\xi |\le |\tau |^{1/m}$ ($\tau <-1$), from inequality (2.33) we have

2.35 $$\begin{aligned} I(\xi ,\tau ) \lesssim (1+|\xi |)^{2s+m}|\tau |^{2b} \frac{|\tau |^{\frac{2n-2}{m}}}{|\xi |^{2n}+1}, \quad s\in \mathbb {R}, \,\, b\ge 0. \end{aligned}$$

Using the inequality (2.35), choosing $n=s+\frac{m+1}{2}+\varepsilon$ and integrating over $\xi$, we again obtain the desired inequality (2.29). This completes the proof of Lemma 2.1. $\square$

Now, we have completed the estimation of the solution to the reduced ibvp. This result will be utilized to prove the linear estimates for the forced ibvp, specifically Theorem 1.1. Next, we accomplish this by decomposing the ibvp (1.5) into four simpler linear problems and estimating each individual problem.

### Decomposition of linear problem

First, we decompose the forced ibvp (1.5) into the homogeneous ibvp

2.36a $$\begin{aligned}&iu_t+(-1)^{j+1}\partial _x^mu = 0,  &   x\in \mathbb {R}^+, \,\, t\in (0,T), \end{aligned}$$

2.36b $$\begin{aligned}&u(x,0) = u_0(x),  &   x\in \mathbb {R}^+, \end{aligned}$$

2.36c $$\begin{aligned}&u(0,t) = g_0(t), \ldots , \partial _x^{j-1}u(0,t) =g_{j-1}(t),  &   t\in (0,T). \end{aligned}$$

and the companion forced ibvp with zero data

2.37a $$\begin{aligned}&iu_t+(-1)^{j+1}\partial _x^mu = f(x,t),  &   x\in \mathbb {R}^+, \,\, t\in (0,T), \end{aligned}$$

2.37b $$\begin{aligned}&u(x,0) = 0,  &   x\in \mathbb {R}^+, \end{aligned}$$

2.37c $$\begin{aligned}&u(0,t) = 0, \ldots , \partial _x^{j-1}u(0,t) = 0,  &   t\in (0,T), \end{aligned}$$

obtaining the superposition relation $S\big [u_0, g_0,\ldots ,g_{j-1}; f\big ] = S\big [u_0, g_0,\ldots ,g_{j-1}; 0\big ]+ S\big [0, 0,\ldots ,0; f\big ].$ The homogeneous ibvp (2.36) has the following compatibility conditions

2.38 $$\begin{aligned} \partial _x^\ell u_0(0) = g_\ell (0), \quad \text {for } \ell \,\, \text {and} \,\, s \text { such that } \,\, \frac{2s+m-1-2\ell }{2m}> \frac{1}{2} \ \left( \text {or } \, s>\ell +\frac{1}{2} \right) .\nonumber \\ \end{aligned}$$

Next, we decompose homogeneous ibvp (2.36) into an ivp and a pure ibvp (with zero initial data). The homogeneous ivp is given by

2.39a $$\begin{aligned}&iU _t+ (-1)^{j+1}\partial _x^mU = 0,  &   x\in \mathbb R,\ t\in (0, T), \end{aligned}$$

2.39b $$\begin{aligned}&U (x,0) = U_0(x),  &   x\in \mathbb {R}, \end{aligned}$$

where $U_0\in H_x^{s}(\mathbb {R})$ is an extension of the initial datum $u_0\in H_x^{s}(0, \infty )$ such that $\Vert U_0\Vert _{H_x^{s}(\mathbb {R})} \le 2\Vert u_0\Vert _{H_x^{s}(0, \infty )},$ and its solution is given by the formula

2.40 $$\begin{aligned} U (x,t) = S\big [U_0; 0\big ] (x, t) = \frac{1}{2\pi } \int _{\mathbb {R}} e^{i\xi x-i\xi ^mt}\, \widehat{U}_0(\xi ) d\xi ,\quad (x,t)\in \mathbb {R}^2, \end{aligned}$$

where $\widehat{U}_0(\xi ) = \int _{\mathbb {R}} e^{-i\xi x}\, U_0(x) dx$. Also, for this ivp we have the following result.

#### Proposition 2.1

For $s, b\in \mathbb {R}$, the solution $S\big [U_0; 0\big ]$ in (2.40) satisfies the space estimate

2.41 $$\begin{aligned} \Vert \psi \cdot S\big [U_0; 0\big ]\Vert _{X^{{s},b}} \le c_{\psi ,b} \Vert U_0\Vert _{H_x^{s}(\mathbb {R})}, \end{aligned}$$

where $c_{\psi ,b}$ is a constant depending only on $\psi$ and *b*. Also, $S[U_0;0]$ satisfies the time estimate

2.42 $$\begin{aligned}&\sup _{x\in \mathbb {R}}\Vert \psi (\cdot ) \, \partial _x^\ell S\big [U_0; 0\big ](x)\Vert _{H_t^{\frac{2s+m-1-2\ell }{2m}}(\mathbb {R})} \lesssim \Vert U_0\Vert _{H_x^{s}(\mathbb {R})}, \quad \ell =0,1,\ldots ,j-1. \end{aligned}$$

#### Proof of Proposition 2.1

The space estimate (2.41) is derived by straightforward computation of Bourgain norm. The derivation of the time estimate (2.42), is similar to the corresponding ones in the case of KdVm equation [34] and the Schrödinger equation [24]. $\square$

The pure ibvp, which is companion of ivp (2.39), is given by

2.43a $$\begin{aligned}&iu_t+(-1)^{j+1}\partial _x^mu = 0, \quad x\in \mathbb {R}^+, \ t\in (0,T), \end{aligned}$$

2.43b $$\begin{aligned}&u(x,0) = 0, \quad x\in \mathbb {R}^+, \end{aligned}$$

2.43c $$\begin{aligned}&\partial _x^{\ell }u(0,t) = g_{\ell }(t)-\partial _x^{\ell }U(0,t) \doteq G_\ell (0,t), \quad \ell =0,1,\ldots ,j-1, \quad t\in (0,T). \end{aligned}$$

Its compatibility conditions that are derived from (2.38) are given by

2.44 $$\begin{aligned} G_{\ell }(0,t) = 0, \quad \text {for } \ell \,\, \text {and} \,\, s \text { such that } \,\, \frac{2s+m-1-2\ell }{2m}> \frac{1}{2} \quad (\text {or } \, s>\ell +\frac{1}{2} ).\qquad \end{aligned}$$

The solution of the pure ibvp (2.43) $S\big [0,G_0,\ldots ,G_{j-1};0\big ]$ is defined by UTM formula (1.6) and satisfies the estimates contained in the next result.

#### Proposition 2.2

If $-\frac{1}{2}m-\frac{1}{2}<s< \frac{1}{2}m+\frac{1}{2}$, $s\ne \frac{1}{2},\frac{3}{2},\ldots ,j-\frac{1}{2}$ and $0<b<1/2$, then the solution to pure ibvp (2.43), which is equipped with the compatibility conditions (2.44), satisfies the following estimate in Bourgain spaces

2.45 $$\begin{aligned} \Vert S\big [0,G_0,\ldots ,G_{j-1};0\big ]\Vert _{X^{s,b}_{\mathbb {R}^+\times [0,T]}} \lesssim \sum \limits _{\ell =0}^{j-1} \Vert G_\ell \Vert _{H^{\frac{2s+m-1-2\ell }{2m}}(0,T)}. \end{aligned}$$

#### Proof of Proposition 2.2

The proof of Proposition 2.2 relies on the theorem established for the reduced pure ibvp (2.1), that is Theorem 2.1. To apply this theorem, we need to use appropriate extensions. These extensions and all the estimations needed are similar to the ones used in the proof of Theorem 3.2 in [34] and we omit them here. $\square$

Finally, we describe the other two simpler problems. We obtain them, by decomposing the forced ibvp with zero data (2.37) to a forced ivp with zero initial data and a pure ibvp. The forced ivp is given by

2.46a $$\begin{aligned}&iW_t+ (-1)^{j+1}\partial _x^mW = w(x, t) ,  &   x\in \mathbb {R},\ t\in (0, T), \end{aligned}$$

2.46b $$\begin{aligned}&W(x,0) = 0,  &   x\in \mathbb R, \end{aligned}$$

where $w \in X^{s,-b}\cap Y^{s,-b}$ is an extension of the forcing $f \in X_{\mathbb {R}^+\times [0,T]}^{s,-b}\cap Y_{\mathbb {R}^+\times [0,T]}^{s,-b}$ such that $\Vert w\Vert _{X^{s,-b}} + \Vert w\Vert _{Y^{s,-b}} \leqslant 2 \Vert f\Vert _{X_{\mathbb {R}^+\times [0,T]}^{s,-b}} + 2 \Vert f\Vert _{Y_{\mathbb {R}^+\times [0,T]}^{s,-b}}.$ The solution of this problem is given by the Duhamel formula

2.47 $$\begin{aligned} W(x,t) \doteq S\big [0; w\big ](x, t)= &   \frac{-i}{2\pi } \int _{\mathbb {R}} \int _{t'=0}^t e^{i\xi x-i\xi ^m(t-t')} {\widehat{w}}(\xi , t') dt' d\xi \end{aligned}$$

2.48 $$\begin{aligned}= &   -i \int _{t'=0}^t S\big [w(\cdot , t'); 0\big ](x, t-t') dt', \end{aligned}$$

where $\widehat{w}$ is the Fourier transform of *w* with respect to *x*, and $S\big [w(\cdot , t'); 0\big ]$ in the Duhamel representation (2.48) denotes the solution (2.40) of ivp (2.39) with $w(x, t')$ in place of the initial datum $U_0(x)$. For this problem, we have the following result.

#### Proposition 2.3

For $0\le b<\frac{1}{2}$, the solution (2.47) satisfies the following space and time estimates

2.49 $$\begin{aligned}  &   \Vert \psi S\big [0; w\big ]\Vert _{X^{s,b}} \lesssim \Vert w\Vert _{X^{s,-b}}, \quad s\in \mathbb {R}, \end{aligned}$$

2.50 $$\begin{aligned}  &   \quad \sup \limits _{x\in \mathbb {R}} \Vert \psi (\cdot ) \cdot \partial _x^\ell S[0,w](x) \Vert _{H_t^{\frac{2s+m-1-2\ell }{2m}}(\mathbb {R})}\nonumber \\  &   \quad \lesssim {\left\{ \begin{array}{ll} \Vert w\Vert _{X^{s,-b}}, \quad -\frac{1}{2}\le s \le \frac{1}{2}, \\ \Vert w\Vert _{X^{s,-b}} + \Vert w\Vert _{Y^{s,-b}}, \quad s\in \mathbb {R}, \end{array}\right. } \,\, \ell =0,1,\ldots ,j-1.\nonumber \\ \end{aligned}$$

#### Proof of Proposition 2.3

We obtain both estimates by decomposing $\psi (t) \partial _{x}^\ell S\big [0; w\big ](x,t)$ as follows

2.51 $$\begin{aligned} \psi (t) \partial _{x}^\ell S\big [0; w\big ](x,t)&\simeq \psi (t) \int _{\mathbb {R}^2} e^{i(\xi x+\tau t)} \frac{1-\psi (\tau +\xi ^m)}{\tau +\xi ^m} \cdot (i\xi )^\ell \widehat{w}(\xi ,\tau ) d\tau d\xi \nonumber \\&\quad - \psi (t) \cdot \partial _{x}^\ell \int _{\mathbb {R}^2} e^{i(\xi x-\xi ^mt)} \frac{1-\psi (\tau +\xi ^m)}{\tau +\xi ^m} \widehat{w}(\xi ,\tau ) d\tau d\xi \nonumber \\&\quad + \psi (t) \cdot \partial _{x}^\ell \int _{\mathbb {R}^2} e^{i(\xi x-\xi ^m t)} \frac{\psi (\tau +\xi ^m)[e^{i(\tau +\xi ^m)t} - 1]}{\tau +\xi ^m} \widehat{w}(\xi ,\tau ) d\tau d\xi . \end{aligned}$$

The estimation of the above three terms is similar to the estimation of the corresponding terms for KdVm presented in Theorem 3.3 in [34]. $\square$

The companion to forced ivp (2.46) resulting from the splitting of ibvp (2.37) is the pure ibvp

2.52a $$\begin{aligned}&iu_t+u_{xx} = 0,  &   x\in \mathbb {R}^+, \ t\in (0,T), \end{aligned}$$

2.52b $$\begin{aligned}&u(x,0) = 0,  &   x\in \mathbb {R}^+, \end{aligned}$$

2.52c $$\begin{aligned}&\partial _x^{\ell }u(0,t) = -\partial _x^{\ell }W(0,t) \doteq W_\ell (0,t), \quad \ell =0,1,\ldots ,j-1,  &   t\in (0,T). \end{aligned}$$

The above ibvp is equipped with the compatibility conditions

2.53 $$\begin{aligned} W_{\ell }(0,t) = 0, \quad \text {for } \ell \,\, \text {and} \,\, s \text { such that } \,\, \frac{2s+m-1-2\ell }{2m}> \frac{1}{2} \quad \left( \text {or } \, s>\ell +\frac{1}{2} \right) .\nonumber \\ \end{aligned}$$

Its solution $S\big [0, W_0,\ldots ,W_{j-1}; 0\big ]$ is defined by UTM formula (1.6), and satisfies next result.

#### Proposition 2.4

If $-\frac{1}{2}m-\frac{1}{2}<s< \frac{1}{2}m+\frac{1}{2}$, $s\ne \frac{1}{2},\frac{3}{2},\ldots ,j-\frac{1}{2}$, and $0<b<1/2$, then the solution to the pure ibvp (2.52) that is equipped with compatibility conditions (2.53) satisfies the following estimate

2.54 $$\begin{aligned} \Vert S\big [0,W_0,\ldots ,W_{j-1};0\big ]\Vert _{X^{s,b}_{\mathbb {R}^+\times [0,T]}} \lesssim \sum \limits _{\ell =0}^{j-1} \Vert W_\ell \Vert _{H^{\frac{2s+m-1-2\ell }{2m}}(0,T)}. \end{aligned}$$

#### Proof of Proposition 2.4

By applying Proposition 2.2 with the boundary data $G_\ell =W_\ell$, we obtain the desired result. Therefore, the proof of Proposition 2.4 is completed. $\square$

#### Proof Linear estimates (Theorem 1.1)

The linear estimate (1.20) can be established by applying the superposition principle to the linear problem (1.5) and utilizing Propositions 2.1–2.4. Therefore, by combining the estimates satisfied by the components problems, we derive the main linear estimate (1.20) and complete the proof of Theorem 1.1. $\square$

## $\bar{f}\bar{g}$ bilinear estimates in spatial Bourgain spaces

In this section, first we prove the spatial bilinear estimates (1.21) for the symmetric nonlinearity $N_3(f, g)= \bar{f}\bar{g}$, and then we demonstrate their optimality. We note that the Bourgain quantity $d_3(\xi , \xi _1)$ of this nonlinearity is elliptic.

### $\bar{f}\bar{g}$ spatial bilinear estimates

Following [6], for a function *h* we let $c_h$ be the combination

3.1 $$\begin{aligned} c_h(\xi ,\tau ) \doteq (1+|\xi |)^s(1+|\tau +\xi ^m|)^{b'}|\widehat{h}(\xi ,\tau )|. \end{aligned}$$

Using it we see that the Bourgain norm of *h* is the $L^2$ norm of $c_h$, that is

3.2 $$\begin{aligned} \Vert h\Vert _{X^{s,b'}} = \Vert c_h(\xi ,\tau )\Vert _{L^2_{\xi ,\tau }}, \end{aligned}$$

and the bilinear estimate (1.21) for the nonlinearity $\bar{f}\bar{g}$ takes the form

3.3 $$\begin{aligned} \Vert \bar{f}\bar{g}\Vert _{X^{s,-b}} \le c_{s,b} \Vert c_{f} \Vert _{L^2_{\xi ,\tau }} \Vert c_{g} \Vert _{L^2_{\xi ,\tau }}. \end{aligned}$$

Furthermore, using the identity $\widehat{\bar{f}}(\xi ,\tau ) = \overline{\widehat{f}\,}(-\xi ,-\tau )$ and notation (3.1) we get

$$\begin{aligned}&|\widehat{\bar{f}\bar{g}}(\xi ,\tau )| \le \\&\quad \int _{\mathbb {R}^2} \frac{c_{f}(-(\xi -\xi _1),-(\tau -\tau _1))}{(1+|\xi -\xi _1|)^s (1+|\tau -\tau _1-(\xi -\xi _1)^m|)^{b'}} \frac{c_{g}(-\xi _1,-\tau _1)}{(1+|\xi _1|)^s(1+|\tau _1-\xi _1^{m}|)^{b'}} d\xi _1 d\tau _1, \end{aligned}$$

which implies that

$$\begin{aligned} \Vert \bar{f}\bar{g}\Vert _{X^{s,-b}}&= \big \Vert (1+|\xi |)^s(1+|\tau +\xi ^m|)^{-b}\widehat{\bar{f}\bar{g}}(\xi ,\tau )\big \Vert _{L^2_{\xi ,\tau }} \\&\lesssim \Big \Vert \frac{(1+|\xi |)^s}{(1+|\tau +\xi ^m|)^{b}} \int _{\mathbb {R}^2} \frac{ c_{f}(-(\xi -\xi _1),-(\tau -\tau _1)) }{(1+|\xi -\xi _1|)^s (1+|\tau -\tau _1-(\xi -\xi _1)^m|)^{b'}}\\&\quad \ \times \frac{ c_{g}(-\xi _1,-\tau _1) }{ (1+|\xi _1|)^s (1+|\tau _1-\xi _1^{m}|)^{b'} } d\xi _1 d\tau _1 \Big \Vert _{L^2_{\xi ,\tau }}. \end{aligned}$$

Then, moving $\frac{(1+|\xi |)^s}{(1+|\tau +\xi ^m|)^{b}}$ inside the integral, and the Sobolev multipliers $(1+|\xi -\xi _1|)^{s}$, $(1+|\xi _1|)^{s}$ from the denominator to the numerator, we see that to prove bilinear estimate (1.21) it suffices to show that

3.4 $$\begin{aligned}&\Big \Vert \int _{\mathbb {R}^2} \frac{(1+|\xi |)^s}{(1+|\tau +\xi ^m|)^{b}} \frac{(1+|\xi -\xi _1|)^{-s}c_{f}(-(\xi -\xi _1),-(\tau -\tau _1))}{ (1+|\tau -\tau _1-(\xi -\xi _1)^m|)^{b'} } \frac{(1+|\xi _1|)^{-s}c_{g}(-\xi _1,-\tau _1)}{(1+|\tau _1-\xi _1^{m}|)^{b'}} d\xi _1 d\tau _1 \Big \Vert _{L^2_{\xi ,\tau }} \nonumber \\&\lesssim \left\| c_{f}\right\| _{L^2_{\xi ,\tau }} \left\| c_{g}\right\| _{L^2_{\xi ,\tau }}. \end{aligned}$$

Next, using the fact that $\Vert c_{f}(\cdot ,\cdot )\Vert _{L^2_{\xi ,\tau }} = \Vert c_{f}(-\cdot ,-\cdot )\Vert _{L^2_{\xi ,\tau }}$ and $\Vert c_{g}(\cdot ,\cdot )\Vert _{L^2_{\xi ,\tau }} = \Vert c_{g}(-\cdot ,-\cdot )\Vert _{L^2_{\xi ,\tau }},$ we see that in order to prove $L^2$ bilinear estimate (3.4), it suffices to show the following reduced $L^2$ bilinear estimate

3.5 $$\begin{aligned}&\Big \Vert \int _{\mathbb {R}^2} \frac{(1+|\xi |)^s}{(1+|\tau +\xi ^m|)^{b}} \frac{(1+|\xi -\xi _1|)^{-s}c_{f}(\xi -\xi _1,\tau -\tau _1)}{ (1+|\tau -\tau _1-(\xi -\xi _1)^m|)^{b'} } \frac{(1+|\xi _1|)^{-s}c_{g}(\xi _1,\tau _1)}{(1+|\tau _1-\xi _1^{m}|)^{b'}} d\xi _1 d\tau _1 \Big \Vert _{L^2_{\xi ,\tau }} \nonumber \\ \lesssim&\Vert c_{f}\Vert _{L^2_{\xi ,\tau }} \Vert c_{g}\Vert _{L^2_{\xi ,\tau }}. \end{aligned}$$

Finally, collecting all multipliers together to form the quantity

3.6 $$\begin{aligned}&Q \doteq \frac{ (1+|\xi |)^s }{ (1+|\xi _1|)^{s} (1+|\xi -\xi _1|)^{s} } \cdot \frac{1}{(1+|\tau +\xi ^m|)^{b}} \nonumber \\&\cdot \frac{1}{ (1+|\tau _1-\xi _{1}^{m}|)^{b'} (1+|\tau -\tau _1-(\xi -\xi _1)^m|)^{b'} }, \end{aligned}$$

we see that inequality (3.5) reads as follows

3.7 $$\begin{aligned} \Big \Vert \int _{\mathbb {R}^2} Q(\xi _1,\tau _1,\xi ,\tau ) \cdot c_{f}(\xi -\xi _1,\tau -\tau _1) c_{g}(\xi _1,\tau _1) d\xi _1 d\tau _1 \Big \Vert _{L^2_{\xi ,\tau }} \lesssim \Vert c_{f}\Vert _{L^2_{\xi ,\tau }} \Vert c_{g}\Vert _{L^2_{\xi ,\tau }}. \end{aligned}$$

At this point we utilize the symmetry of nonlinearity $\bar{u}\bar{u}$, which combined with the symmetry in the definition of convolution, allows us to assume that $|\tau -\tau _1-(\xi -\xi _1)^m| \le |\tau _1-\xi _1^m|,$ and therefore to replace the quantity *Q* with $\chi _A\cdot Q$, where

3.8 $$\begin{aligned} A \doteq \{ (\xi _1,\tau _1,\xi ,\tau )\in \mathbb {R}^4: |\tau -\tau _1-(\xi -\xi _1)^m| \le |\tau _1-\xi _1^m| \}. \end{aligned}$$

Moreover, since $b' \le b$ if in the multiplier *Q* defined by (3.6) we replace *b* by $b'$, then the resulting new multiplier becomes bigger than *Q*.

Therefore, we see that in order to prove the $L^2$ bilinear estimate (3.7), it suffices to show the following reduced $L^2$ bilinear estimate

3.9 $$\begin{aligned} \Big \Vert \int _{\mathbb {R}^2} Q' \cdot \chi _{A} \cdot c_{f}(\xi -\xi _1,\tau -\tau _1) c_{g}(\xi _1,\tau _1) d\xi _1 d\tau _1 \Big \Vert _{L^2_{\xi ,\tau }} \lesssim \Vert c_{f}\Vert _{L^2_{\xi ,\tau }} \Vert c_{g}\Vert _{L^2_{\xi ,\tau }},\qquad \end{aligned}$$

where the new (more symmetric) multiplier $Q'$ involving only $b'$ (not *b*) is given by

3.10 $$\begin{aligned}&Q' \doteq \frac{ (1+|\xi |)^s }{ (1+|\xi _1|)^{s} (1+|\xi -\xi _1|)^{s} } \cdot \frac{1}{(1+|\tau +\xi ^m|)^{b'}} \nonumber \\&\cdot \frac{1}{ (1+|\tau _1-\xi _{1}^{m}|)^{b'} (1+|\tau -\tau _1-(\xi -\xi _1)^m|)^{b'} }. \end{aligned}$$

Next, in the multiplier $Q'$ (or *Q*) we recognize the Bourgain quantity for the nonlinearity $\bar{f}\bar{g}$

3.11 $$\begin{aligned} d_{3,m}(\xi ,\xi _1) \doteq \xi ^m+\xi _1^m+(\xi -\xi _1)^m  &   = (\tau +\xi ^m)-(\tau _1-\xi _1^m)\nonumber \\  &   \quad -[(\tau -\tau _1)-(\xi -\xi _1)^m]. \end{aligned}$$

Below we list some useful and elementary properties of $d_{3,m}(\xi ,\xi _1)$.

#### Proposition 3.1

If $m=2j\ge 2$ is an even number, then $d_{3,m}(\xi ,\xi _1)$ is a symmetric homogeneous polynomial of $\xi$ and $\xi _1$ of degree *m*, and has the following properties:

3.12 $$\begin{aligned}  &   \bullet \text { Comparison Property:} \qquad \max \{ |\tau +\xi ^m|, |\tau _1-\xi _1^m|, |\tau -\tau _1-(\xi -\xi _1)^m| \} \nonumber \\  &   \qquad \ge \frac{1}{3} d_{3,m}(\xi ,\xi _1). \end{aligned}$$

3.13 $$\begin{aligned}  &   \bullet \text { Lower bound for }d_{3,m}(\xi ,\xi _1): \quad d_{3,m}(\xi ,\xi _1) \ge \max \{\xi ^m,\xi _1^m,(\xi -\xi _1)^m\}. \end{aligned}$$

3.14 $$\begin{aligned}  &   \bullet \,\, \partial _\xi d_{3,m}(\xi ,\xi _1) = m\xi ^{m-1}+m(\xi -\xi _1)^{m-1} \quad \text {and} \quad \partial _{\xi _1} d_{3,m}(\xi ,\xi _1) \nonumber \\  &   \qquad = m\xi _1^{m-1}+m(\xi _1-\xi )^{m-1}. \end{aligned}$$

3.15 $$\begin{aligned}  &   \bullet \text { Lower bound for }\partial _\xi d_{3,m}(\xi ,\xi _1): \quad \,\, |\partial _\xi d_{3,m}(\xi ,\xi _1)| \nonumber \\  &   \qquad \gtrsim \max \big \{ |\xi -\frac{1}{2}\xi _1|^{m-1}, |\xi -\frac{1}{2}\xi _1|\xi _1^{m-2} \big \}. \end{aligned}$$

3.16 $$\begin{aligned}  &   \bullet \text { Lower bound for }\partial _{\xi _1} d_{3,m}(\xi ,\xi _1): \quad |\partial _{\xi _1} d_{3,m}(\xi ,\xi _1)|\nonumber \\  &   \qquad \gtrsim \max \big \{ |\xi _1-\frac{1}{2}\xi |^{m-1}, |\xi _1-\frac{1}{2}\xi |\xi ^{m-2} \big \}. \end{aligned}$$

$\bullet$ Critical points: When considering $d_{3,m}(\xi ,\xi _1)$ as a function of $\xi$, we can determine that its critical point occurs at $\xi =\frac{1}{2}\xi _1$. When considering $d_{3,m}(\xi ,\xi _1)$ as a function of $\xi _1$, we can determine that its critical point occurs at $\xi _1=\frac{1}{2}\xi$.

**Fig. 6:**
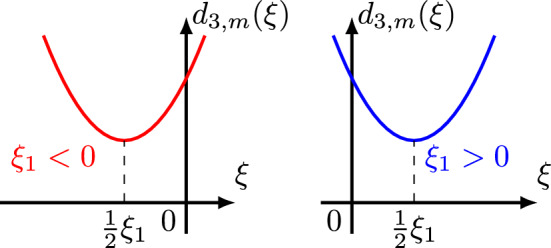
$d_{3,m}$ is a function of $\xi$

**Fig. 7:**
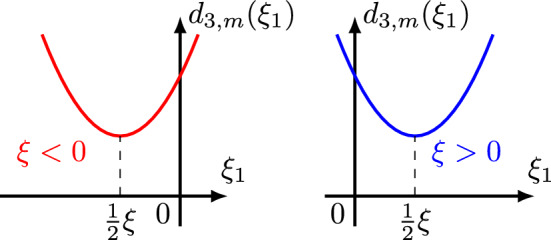
$d_{3,m}$ is a function of $\xi _1$

$\bullet$ Graphs: Figs. 6 and 7 display the graph of $d_{3,m}(\xi ,\xi _1)\ge 0$ as a function of $\xi$ and $\xi _1$, respectively, noticing that $d_{3,m}(\xi ,\frac{1}{2}\xi )=\frac{3}{2}\xi ^m$ and $d_{3,m}(\frac{1}{2}\xi _1,\xi _1)=\frac{3}{2}\xi _1^m$ are minimum values.

#### Proof of Proposition 3.1

The proof of $d_{3,m}$ properties is elementary. We give only the proof of the lower bound (3.15). For this, we define $\eta \doteq \xi - \frac{1}{2}\xi _1$ and rewrite $\partial _\xi d_{3,m}(\xi ,\xi _1) = m\xi ^{m-1} + m(\xi -\xi _1)^{m-1}$ using the symmetric formula

$$\begin{aligned} \partial _\xi d_{3,m}(\xi ,\xi _1)&= m(\eta +\frac{1}{2}\xi _1)^{m-1}+m \left( \eta -\frac{1}{2}\xi _1\right) ^{m-1} \\&= 2m \eta \cdot \Bigg [ \eta ^{m-2}+\left( {\begin{array}{c}m-1\\ 2\end{array}}\right) \eta ^{m-4}\left( \frac{1}{2}\xi _1\right) ^2+ \left( {\begin{array}{c}m-1\\ 4\end{array}}\right) \eta ^{m-6}\left( \frac{1}{2}\xi _1\right) ^4 \\&\quad + \cdots + \left( {\begin{array}{c}m-1\\ m-2\end{array}}\right) (\frac{1}{2}\xi _1)^{m-2} \Bigg ], \end{aligned}$$

where $$ \left( {\begin{array}{c}\alpha \\ \beta \end{array}}\right) = \frac{\alpha !}{\beta ! (\alpha - \beta )!}. $$ Since *m* is an even number, all the terms inside the bracket of the above identity are non-negative. Therefore, by retaining only the first and last terms and taking their absolute values, we obtain the desired inequality (3.15). This completes the proof of Proposition 3.1. $\square$

Next, we provide the proof of $L^2$ bilinear estimate (3.9) for the following two cases:

$$\bullet $$ Case: $$s\ge 0$$       $$\bullet $$ Case: $$s< 0$$.

**Bilinear estimate for** $s\ge 0$. We begin with the first case $s\ge 0$, which is the most important since parts of the other cases reduce to this one. For $s\ge 0$, we have $(1+|\xi |)^s \le (1+|\xi -\xi _1|)^s (1+|\xi _1|)^s$, which implies that $\frac{ (1+|\xi |)^s }{ (1+|\xi -\xi _1|)^s (1+|\xi _1|)^s } \lesssim 1.$ Thus, we have $Q'\le Q_0$, where

3.17 $$\begin{aligned} Q_0(\xi _1,\tau _1,\xi ,\tau ) \doteq \frac{1}{(1+|\tau +\xi ^m|)^{b'}} \frac{1}{ (1+|\tau _1-\xi _{1}^{m}|)^{b'} (1+|\tau -\tau _1-(\xi -\xi _1)^m|)^{b'} }. \end{aligned}$$

Therefore, when $s\ge 0$ to prove (3.9), it suffices to show the following reduced $L^2$ bilinear estimate

3.18 $$\begin{aligned} \Big \Vert \int _{\mathbb {R}^2} \chi _{A} Q_0 \cdot c_{f}(\xi -\xi _1,\tau -\tau _1) c_{g}(\xi _1,\tau _1) d\xi _1 d\tau _1 \Big \Vert _{L^2_{\xi ,\tau }} \lesssim \Vert c_{f}\Vert _{L^2_{\xi ,\tau }} \Vert c_{g}\Vert _{L^2_{\xi ,\tau }}, \end{aligned}$$

which corresponds to proving the bilinear estimate when $s=0$. Next, comparing $|\tau _1-\xi _1^m|$ and $|\tau +\xi ^{m}|$ leads us to considering the following familiar microlocalizations:

$\bullet$**Microlocalization I**. In this microlocalization the region $A_{I}$ is defined as

3.19 $$\begin{aligned} A_{I} \doteq \big \{ (\xi _1,\tau _1,\xi ,\tau ) \in \mathbb {R}^4: |\tau -\tau _1-(\xi -\xi _1)^m| \le |\tau _1-\xi _1^m| \le |\tau +\xi ^{m}| \big \}. \end{aligned}$$

$\bullet$**Microlocalization II**. In this microlocalization, the region $A_{II}$ is defined as

3.20 $$\begin{aligned} A_{II} \doteq \big \{ (\xi _1,\tau _1,\xi ,\tau ) \in \mathbb {R}^4: |\tau -\tau _1-(\xi -\xi _1)^m| \le |\tau _1-\xi _1^m| \,\, \text {and} \,\, |\tau +\xi ^{m}|< |\tau _1-\xi _1^m| \big \}. \end{aligned}$$

**Proof in Microlocalization I.** In this microlocalization, we have $|\tau +\xi ^m|=\max \big \{ |\tau +\xi ^m|, |\tau _1-\xi _1^m|, \big | \tau -\tau _1-(\xi -\xi _1)^m \big | \big \}$. Also, the $L^2$ bilinear estimate (3.18) becomes

3.21 $$\begin{aligned} \Big \Vert \int _{\mathbb {R}^2} (\chi _{A_{I}}Q_0) (\xi _1,\tau _1,\xi ,\tau ) \cdot c_{f}(\xi -\xi _1,\tau -\tau _1) c_{g}(\xi _1,\tau _1) d\xi _1 d\tau _1 \Big \Vert _{L^2_{\xi ,\tau }} \lesssim \Vert c_{f}\Vert _{L^2_{\xi ,\tau }} \Vert c_{g}\Vert _{L^2_{\xi ,\tau }}. \end{aligned}$$

By utilizing the Cauchy–Schwarz inequality with respect to the variables $\xi _1$ and $\tau _1$, we have

$$\begin{aligned} \text {LHS of }{(3.2.1)}&\lesssim \Big \Vert \int _{\mathbb {R}^2} (\chi _{A_I} Q_0^2)(\xi _1,\tau _1,\xi ,\tau ) d\xi _1 d\tau _1 \Big \Vert _{L^\infty _{\xi ,\tau }}^{1/2} \Big \Vert \\&\quad \left( \int _{\mathbb {R}^2} c^2_{f}(\xi -\xi _1,\tau -\tau _1) c^2_{g}(\xi _1,\tau _1)d\xi _1 d\tau _1 \right) ^{1/2}\Big \Vert _{L^2_{\xi ,\tau }}. \end{aligned}$$

And, since $\big \Vert \left( \int _{\mathbb {R}^2}c^2_{f}(\xi -\xi _1,\tau -\tau _1) c^2_{g}(\xi _1,\tau _1)d\xi _1 d\tau _1 \right) ^{1/2} \big \Vert _{L^2_{\xi ,\tau }} = \Vert c_{f}\Vert _{L^2_{\xi } L^2_{\tau }} \Vert c_{g}\Vert _{L^2_{\xi } L^2_{\tau }},$ we obtain

3.22 $$\begin{aligned} \text {LHS of }{(3.2.1)} \lesssim \Big \Vert \int _{\mathbb {R}^2} (\chi _{A_{I}} Q_0^2) (\xi _1,\tau _1,\xi ,\tau ) d\xi _1 d\tau _1 \Big \Vert _{L^{\infty }_{\xi ,\tau } }^{1/2} \cdot \Vert c_{f}\Vert _{L^2_{\xi ,\tau }} \Vert c_{g}\Vert _{L^2_{\xi ,\tau }}. \end{aligned}$$

Thus, to prove $L^2$ bilinear estimate (3.21), it suffices to show the following result.

#### Lemma 3.1

Let $m=2j\ge 2$ be an even number. If $\frac{1}{3}\le b'<\frac{1}{2}$, then for $\xi ,\tau \in \mathbb {R}$, we have

3.23 $$\begin{aligned} \Theta _{1} (\xi , \tau )&\doteq \frac{1}{ (1+|\tau +\xi ^{m}|)^{2b'} } \int _{\mathbb {R}^2} \nonumber \\&\quad \frac{ \chi _{A_{I}} (\xi _1,\tau _1,\xi ,\tau ) }{ (1+|\tau -\tau _1-(\xi -\xi _1)^m|)^{2b'} (1+|\tau _1-\xi _1^m|)^{2b'} } d\tau _1 d\xi _1 \lesssim 1. \end{aligned}$$

For proving it, we need the following calculus estimate, whose proof can be found in [39].

#### Lemma 3.2

If $$1/4<\ell ', \ell <1/2$$, then

3.24 $$\begin{aligned} \int _{\mathbb {R}}\frac{dx}{(1+|x-a|)^{2\ell }(1+|x-c|)^{2\ell '}} \lesssim \frac{1}{(1+|a-c|)^{2\ell +2\ell '-1}}. \end{aligned}$$

#### Proof of Lemma 3.1

For the $\tau _1$ integral in (3.23), applying calculus estimate (3.24) with $\ell =\ell '=b'>\frac{1}{4}$, $x=\tau _1$, $\alpha =\tau -(\xi -\xi _1)^m$, $\beta =\xi _1^m$, we get

3.25 $$\begin{aligned} \Theta _{1}(\xi , \tau )&\lesssim \frac{1}{ (1+|\tau +\xi ^{m}|)^{2b'} } \int _{\mathbb {R}} \frac{ \chi _{A_{I}} (\xi _1,\tau _1,\xi ,\tau ) }{ (1+|\tau -(\xi -\xi _1)^m-\xi _1^m|)^{4b'-1} } d\xi _1 \nonumber \\&= \frac{1}{ (1+|\tau +\xi ^{m}|)^{2b'} } I_1(\xi ,\tau ), \end{aligned}$$

where the integral $I_1$ is defined as follows

3.26 $$\begin{aligned} I_1(\xi ,\tau )&\doteq \int _{\mathbb {R}} \frac{ \chi _{A_{I}} (\xi _1,\tau _1,\xi ,\tau ) }{ (1+|\tau -(\xi -\xi _1)^m-\xi _1^m|)^{4b'-1} } d\xi _1 \nonumber \\&= \int _{\mathbb {R}} \frac{ \chi _{A_{I}} (\xi _1,\tau _1,\xi ,\tau ) }{ (1+|\tau +\xi ^m-d_{3,m}(\xi ,\xi _1)|)^{4b'-1} } d\xi _1. \end{aligned}$$

Here, $d_{3,m}(\xi ,\xi _1)=\xi ^m+\xi _1^m+(\xi -\xi _1)^m$ is the Bourgain quantity for nonlinearity $\bar{f}\bar{g}$, which is defined by (3.11). We claim that the integral $I_1$ can be bounded as follows

3.27 $$\begin{aligned} I_1(\xi ,\tau ) \lesssim (1+|\tau +\xi ^m|)^{2-4b'}, \quad \frac{1}{4}\le b'<\frac{1}{2}. \end{aligned}$$

We will prove inequality (3.27) below. Now, combining inequality (3.27) with inequality (3.25), we obtain

3.28 $$\begin{aligned} \Theta _{1}(\xi , \tau ) \lesssim \frac{1}{(1+|\tau +\xi ^m|)^{2b'}} (1+|\tau +\xi ^m|)^{2-4b'} = \frac{1}{(1+|\tau +\xi ^m|)^{6b'-2}}, \end{aligned}$$

which is bounded if $6b'-2\ge 0$ or $b'\ge \frac{1}{3}$. This completes the proof of Lemma 3.1. $\square$

#### Proof of inequality (3.27)

In integral (3.26), we would like to make the change of variables $\mu =\mu (\xi _1)=\tau +\xi ^m-d_{3,m}(\xi ,\xi _1)$. But for it to be a good change we need to split $\mathbb {R}$ appropriately so that $\mu (\xi _1)$ is a monotone function. Using Proposition 3.1, we can determine that the critical point of the function $d_{3,m}(\xi ,\xi _1)$ occurs at $\xi _1=\frac{1}{2}\xi$. Therefore, splitting the integral of $d\xi _1$ at $\xi _1=\frac{1}{2}\xi$, we obtain

3.29 $$\begin{aligned} I_{1}(\xi , \tau ) = I_1^+(\xi , \tau ) + I_1^-(\xi , \tau ), \end{aligned}$$

where the integrals $I_1^+$ and $I_1^-$ are defined as follows

3.30 $$\begin{aligned}&I_1^+(\xi ,\tau ) \doteq \int _{\frac{1}{2}\xi }^\infty \frac{ \chi _{A_{I}} (\xi _1,\tau _1,\xi ,\tau ) }{ (1+|\tau +\xi ^m-d_{3,m}(\xi ,\xi _1)|)^{4b'-1} } d\xi _1, \end{aligned}$$

3.31 $$\begin{aligned}&I_1^-(\xi ,\tau ) \doteq \int _{-\infty }^{\frac{1}{2}\xi } \frac{ \chi _{A_{I}} (\xi _1,\tau _1,\xi ,\tau ) }{ (1+|\tau +\xi ^m-d_{3,m}(\xi ,\xi _1)|)^{4b'-1} } d\xi _1. \end{aligned}$$

Since the estimation for $I_1^-$ is similar to that of $I_1^+$, here we provide only the estimation of $I_1^+$. Furthermore, we consider the two cases: $\xi _1$ is near the critical point, and $\xi _1$ is away from the critical point. More precisely, we rewrite $I_1^+=I_{1,1}+I_{1,2}$, where

3.32 $$\begin{aligned} I_{1,1}(\xi ,\tau )&\doteq \int _{\frac{1}{2}\xi }^{\frac{1}{2}\xi +1} \frac{ \chi _{A_{I}} (\xi _1,\tau _1,\xi ,\tau ) }{ (1+|\tau +\xi ^m-d_{3,m}(\xi ,\xi _1)|)^{4b'-1} } d\xi _1, \end{aligned}$$

3.33 $$\begin{aligned} I_{1,2}(\xi ,\tau )&\doteq \int _{\frac{1}{2}\xi +1}^\infty \frac{ \chi _{A_{I}} (\xi _1,\tau _1,\xi ,\tau ) }{ (1+|\tau +\xi ^m-d_{3,m}(\xi ,\xi _1)|)^{4b'-1} } d\xi _1. \end{aligned}$$

$\underline{\hbox {Estimate for }I_{1,1}.}$ Since the integral is over the finite interval $(\frac{1}{2}\xi ,\frac{1}{2}\xi +1)$ we have that $I_{1,1}\le 1$, if $4b'-1\ge 0$ or $b'\ge \frac{1}{4}$.

$\underline{\hbox {Estimate for }I_{1,2}.}$ Making the change of variables $\mu =\mu (\xi _1)=\tau +\xi ^m-d_{3,m}(\xi ,\xi _1)$, we get

3.34 $$\begin{aligned} I_{1,2}(\xi , \tau ) \lesssim \int _{\xi _1(\mu )=\frac{1}{2}\xi +1}^\infty \frac{ \chi _{A_{I}} (\xi _1,\tau _1,\xi ,\tau ) }{ (1+|\mu |)^{4b'-1} } \frac{ 1 }{|\partial _{\xi _1}\mu |} d\mu . \end{aligned}$$

Since $\partial _{\xi _1}\mu =-\partial _{\xi _1}d_{3,m}(\xi ,\xi _1)$, using the lower bound for $\partial _{\xi _1}d_{3,m}$, i.e., inequality (3.16), we get $|\partial _{\xi _1}\mu |\gtrsim |\xi _1-\frac{1}{2}\xi |^{m-1}\gtrsim 1$ for $\xi _1(\mu )>\frac{1}{2}\xi +1$. Also, using comparison inequality (3.12), we get

3.35 $$\begin{aligned} |\tau +\xi ^m|  &   = \max \big \{ |\tau +\xi ^m|, |\tau _1-\xi _1^m|, \big | \tau -\tau _1-(\xi -\xi _1)^m \big | \big \} \nonumber \\  &   \ge \frac{1}{3} d_{3,m}(\xi ,\xi _1), \quad (\xi _1,\tau _1,\xi ,\tau )\in A_I. \end{aligned}$$

Thus, $|\mu |\le |\tau +\xi ^m|+|d_{3,m}|\lesssim |\tau +\xi ^m|$ for $(\xi _1,\tau _1,\xi ,\tau ) \in A_I$. Using these inequalities, we get

3.36 $$\begin{aligned} I_{1,2}(\xi , \tau )&\lesssim \int _{\mathbb {R}} \frac{ \chi _{A_{I}} (\xi _1,\tau _1,\xi ,\tau ) }{ (1+|\mu |)^{4b'-1} } d\mu \lesssim \int _{|\mu |\lesssim |\tau +\xi ^m| } \frac{ 1 }{ (1+|\mu |)^{4b'-1} } d\mu \nonumber \\&\lesssim (1+|\tau +\xi ^m|)^{2-4b'}, \quad b'<\frac{1}{2}, \end{aligned}$$

which completes the proof of inequality (3.27). $\square$

#### Proof in Microlocalization II.

Here, we have $|\tau _1-\xi _1^m|=\max \big \{ |\tau +\xi ^m|, |\tau _1-\xi _1^m|, \big | \tau -\tau _1-(\xi -\xi _1)^m \big | \big \}$. Also, the $L^2$ bilinear estimate (3.18) reads as follows

3.37 $$\begin{aligned} \Big \Vert \int _{\mathbb {R}^2} (\chi _{A_{II}}Q_0) (\xi _1,\tau _1,\xi ,\tau ) \cdot c_{f}(\xi -\xi _1,\tau -\tau _1) c_{g}(\xi _1,\tau _1) d\xi _1 d\tau _1 \Big \Vert _{L^2_{\xi ,\tau }} \lesssim \Vert c_{f}\Vert _{L^2_{\xi ,\tau }} \Vert c_{g}\Vert _{L^2_{\xi ,\tau }}. \end{aligned}$$

By duality we have

3.38 $$\begin{aligned} \text {LHS of }{(3.37)} = \sup _{\Vert d\Vert _{L^2_{\xi ,\tau }}=1} \int _{\mathbb {R}^2} d(\xi ,\tau ) \int _{\mathbb {R}^2} \chi _{A_{II}} Q_0 \cdot c_f(\xi -\xi _1,\tau -\tau _1)c_g(\xi _1,\tau _1) d\xi _1 d\tau _1 d\xi d\tau . \nonumber \\ \end{aligned}$$

Next, by applying the Cauchy–Schwarz inequality twice, first in the variables $(\xi _1, \tau _1)$ and then in the variables $(\xi , \tau )$, we obtain the following inequality

3.39 $$\begin{aligned} \text {LHS of }{(3.37)} \le \Vert c_f\Vert _{L^2_{\xi ,\tau }} \Vert c_g\Vert _{L^2_{\xi ,\tau }} \cdot \Big \Vert \int _{\mathbb {R}^2} \chi _{A_{II}} (\xi _1,\tau _1,\xi ,\tau ) \cdot Q_0^2(\xi _1,\tau _1,\xi ,\tau ) d\xi d\tau \Big \Vert ^{\frac{1}{2}}_{L_{\xi _1,\tau _1}^\infty }. \end{aligned}$$

Thus, to prove $L^2$ bilinear estimate (3.37), it suffices to show the following result.

#### Lemma 3.3

Let $m=2j\ge 2$ be an even number. If $\frac{1}{3}\le b'<\frac{1}{2}$, then for $\xi _1,\tau _1\in \mathbb {R}$, we have

3.40 $$\begin{aligned} \Theta _{2} (\xi _1, \tau _1)&\doteq \frac{1}{ (1+|\tau _1-\xi _1^{m}|)^{2b'} }\nonumber \\&\int _{\mathbb {R}^2} \frac{ \chi _{A_{II}} (\xi _1,\tau _1,\xi ,\tau ) }{ (1+|\tau -\tau _1-(\xi -\xi _1)^m|)^{2b'} (1+|\tau +\xi ^m|)^{2b'} } \, d\tau d\xi \lesssim 1. \end{aligned}$$

#### Proof of Lemma 3.3

For the $\tau$ integral in (3.40), applying calculus estimate (3.24) with $\ell =\ell '=b'>\frac{1}{4}$, $x=\tau _1$, $\alpha =\tau _1+(\xi -\xi _1)^m$, $\beta =-\xi ^m$, we get

3.41 $$\begin{aligned} \Theta _{2}&\lesssim \frac{1}{ (1+|\tau _1-\xi _1^{m}|)^{2b'} } \int _{\mathbb {R}}\nonumber \\&\quad \frac{ \chi _{A_{II}} (\xi _1,\tau _1,\xi ,\tau ) }{ (1+|\tau _1+(\xi -\xi _1)^m+\xi ^m|)^{4b'-1} } d\xi \nonumber \\&= \frac{1}{ (1+|\tau _1-\xi _1^{m}|)^{2b'} } I_2(\xi _1,\tau _1), \end{aligned}$$

where the integral $I_2(\xi _1,\tau _1)$ is defined as follows

3.42 $$\begin{aligned} I_2(\xi _1,\tau _1)&\doteq \int _{\mathbb {R}} \frac{ \chi _{A_{II}} (\xi _1,\tau _1,\xi ,\tau ) }{ (1+|\tau _1+(\xi -\xi _1)^m+\xi ^m|)^{4b'-1} } d\xi \nonumber \\&= \int _{\mathbb {R}} \frac{ \chi _{A_{II}} (\xi _1,\tau _1,\xi ,\tau ) }{ (1+|\tau _1-\xi _1^m+d_{3,m}(\xi ,\xi _1)|)^{4b'-1} } d\xi , \end{aligned}$$

where $d_{3,m}(\xi ,\xi _1)=\xi ^m+\xi _1^m+(\xi -\xi _1)^m$ is our familiar Bourgain quantity. Also, we can bound the integral $I_2$ as follows

3.43 $$\begin{aligned} I_2(\xi _1,\tau _1) \lesssim (1+|\tau _1-\xi _1^m|)^{2-4b'}, \quad \frac{1}{4}\le b'<\frac{1}{2}. \end{aligned}$$

In fact, due to the symmetry of $d_{3,m}(\xi ,\xi _1)$ with respect to the variables $\xi$ and $\xi _1$, the proof of inequality (3.43) is similar to the proof of inequality (3.27). Next, combining (3.43) with inequality (3.41) we get

3.44 $$\begin{aligned} \Theta _{2}(\xi _1, \tau _1) \lesssim \frac{1}{(1+|\tau _1-\xi _1^m|)^{2b'}} (1+|\tau _1-\xi _1^m|)^{2-4b'} = \frac{1}{(1+|\tau _1-\xi _1^m|)^{6b'-2}}, \end{aligned}$$

which is bounded if $6b'-2\ge 0$ or $b'\ge \frac{1}{3}$. This completes the proof of Lemma 3.3. $\square$

**Bilinear estimate for** $s< 0$. First, we note that if $|\xi _1|\le 10$ or $|\xi -\xi _1|\le 10$, then we have

3.45 $$\begin{aligned} \frac{ (1+|\xi |)^s }{ (1+|\xi -\xi _1|)^s (1+|\xi _1|)^s } \lesssim 1, \quad \text { if }\, s<0, \end{aligned}$$

which gives us that $Q'\lesssim Q_0$. Thus, for $s<0$ it suffice to prove our bilinear estimate over the region *E* is defined by

3.46 $$\begin{aligned} E \doteq \{ (\xi _1,\tau _1,\xi ,\tau )\in A: |\xi _1|>10 \,\, \text {and} \,\, |\xi -\xi _1|>10 \}. \end{aligned}$$

More precisely, we need to show the following inequality

3.47 $$\begin{aligned} \Big \Vert \int _{\mathbb {R}^2} \chi _{E} Q_1 (\xi _1,\tau _1,\xi ,\tau ) \cdot c_{f}(\xi -\xi _1,\tau -\tau _1) c_{g}(\xi _1,\tau _1) d\xi _1 d\tau _1 \Big \Vert _{L^2_{\xi ,\tau }} \lesssim \Vert c_{f}\Vert _{L^2_{\xi ,\tau }} \Vert c_{g}\Vert _{L^2_{\xi ,\tau }}, \end{aligned}$$

where the multiplier $Q_1$ is defined by

3.48 $$\begin{aligned} Q_1 \doteq \frac{ (1+|\xi |)^s }{ |\xi _1(\xi -\xi _1)|^{s} } \cdot \frac{1}{(1+|\tau +\xi ^m|)^{b'}} \cdot \frac{1}{ (1+|\tau _1-\xi _{1}^{m}|)^{b'} (1+|\tau -\tau _1-(\xi -\xi _1)^m|)^{b'} }.\nonumber \\ \end{aligned}$$

To prove $L^2$ bilinear estimate (3.47), we consider the following two possible microlocalizations:

$\bullet$**Microlocalization I.** It is defined by the region

3.49 $$\begin{aligned} E_{1} \doteq \big \{ (\xi _1,\tau _1,\xi ,\tau ) \in E: |\tau -\tau _1-(\xi -\xi _1)^m| \le |\tau _1-\xi _1^m| \le |\tau +\xi ^m| \big \}. \end{aligned}$$

$\bullet$**Microlocalization II**. It is defined by the region

3.50 $$\begin{aligned} E_{2} \doteq \big \{ (\xi _1,\tau _1,\xi ,\tau ) \in E: |\tau +\xi ^{m}|< |\tau _1-\xi _1^m| \quad \text {and} \quad |\tau -\tau _1-(\xi -\xi _1)^m| \le |\tau _1-\xi _1^m| \big \}. \end{aligned}$$

**Proof in Microlocalization I.** Here, we have $|\tau +\xi ^m|=\max \big \{ |\tau +\xi ^m|, |\tau _1-\xi _1^m|, \big | \tau -\tau _1-(\xi -\xi _1)^m \big | \big \}$. The $L^2$ bilinear estimate (3.47) in this microlocalization reads

3.51 $$\begin{aligned} \Big \Vert \int _{\mathbb {R}^2} \chi _{E_{1}} (\xi _1,\tau _1,\xi ,\tau )&\cdot Q_1 (\xi _1,\tau _1,\xi ,\tau ) \cdot c_{f}(\xi -\xi _1,\tau -\tau _1) c_{g}(\xi _1,\tau _1) d\xi _1 d\tau _1 \Big \Vert _{L^2_{\xi ,\tau }}\nonumber \\&\lesssim \Vert c_{f}\Vert _{L^2_{\xi ,\tau }} \Vert c_{g}\Vert _{L^2_{\xi ,\tau }}. \end{aligned}$$

As in the case of estimate (3.22), using the Cauchy–Schwarz inequality with respect to $\xi _1,\tau _1$, we see that the left-hand side of above estimate is bounded by

3.52 $$\begin{aligned} \Big \Vert \int _{\mathbb {R}^2} \chi _{E_{1}}(\xi _1,\tau _1,\xi ,\tau ) \cdot Q_1^2(\xi _1,\tau _1,\xi ,\tau ) d\xi _1 d\tau _1 \Big \Vert _{L^{\infty }_{\xi ,\tau } }^{1/2} \cdot \Vert c_{f}\Vert _{L^2_{\xi ,\tau }} \Vert c_{g}\Vert _{L^2_{\xi ,\tau }}. \end{aligned}$$

Thus, to prove $L^2$ bilinear estimate (3.51), it suffices to show the following result.

#### Lemma 3.4

Let $m=2j\ge 2$ be an even number. If $-j+\frac{1}{4}<s<0$ and $\max \{\frac{1}{3}-\frac{s}{3\,m},\frac{1}{6}-\frac{4\,s-1}{6\,m}\}\le b'<\frac{1}{2}$, then for $\xi ,\tau \in \mathbb {R}$, we have

3.53 $$\begin{aligned} \Theta _{3}&\doteq \frac{1}{ (1+|\tau +\xi ^{m}|)^{2b'} } \int _{\mathbb {R}^2} \nonumber \\  &\quad \frac{ (1+|\xi |)^{2s} }{ |\xi _1(\xi -\xi _1)|^{2s} } \frac{ \chi _{E_{1}} (\xi _1,\tau _1,\xi ,\tau ) d\tau _1 d\xi _1 }{ (1+|\tau -\tau _1-(\xi -\xi _1)^m|)^{2b'} (1+|\tau _1-\xi _1^m|)^{2b'} } \, \lesssim 1. \end{aligned}$$

#### Proof of Lemma 3.4

For the $\tau _1$ integral in (3.53), applying calculus estimate (3.24) with $\ell =\ell '=b'>\frac{1}{4}$, $x=\tau _1$, $\alpha =\tau -(\xi -\xi _1)^m$, $\beta =\xi _1^m$, we get

3.54 $$\begin{aligned} \Theta _{3}&\lesssim \frac{1}{ (1+|\tau +\xi ^{m}|)^{2b'} } \int _{\mathbb {R}} \nonumber \\  &\quad \frac{ (1+|\xi |)^{2s} }{ |\xi _1(\xi -\xi _1)|^{2s} } \frac{ \chi _{E_{1}} (\xi _1,\tau _1,\xi ,\tau ) \, d\xi _1 }{ (1+|\tau -(\xi -\xi _1)^m-\xi _1^m|)^{4b'-1} } \nonumber \\  &\quad = \frac{1}{ (1+|\tau +\xi ^{m}|)^{2b'} }I_3, \end{aligned}$$

where the integral $I_3$ is defined by

3.55 $$\begin{aligned} I_3(\xi ,\tau ) \doteq&\int _{\mathbb {R}} \frac{ (1+|\xi |)^{2s} }{ |\xi _1(\xi -\xi _1)|^{2s} } \frac{ \chi _{E_{1}} (\xi _1,\tau _1,\xi ,\tau ) }{ (1+|\tau +\xi ^m-d_{3,m}(\xi ,\xi _1)|)^{4b'-1} } d\xi _1, \end{aligned}$$

where $d_{3,m}(\xi ,\xi _1) = \xi ^m+\xi _1^m+(\xi -\xi _1)^m$. Now, we claim the following $I_3$ bound

3.56 $$\begin{aligned}&I_3(\xi ,\tau ) \lesssim \frac{1}{ (1+|\tau +\xi ^{m}|)^{4b'+2s/m-2} } + \frac{1}{ (1+|\tau +\xi ^{m}|)^{4b'+(4s+m-1)/m-2} } , \nonumber \\&\quad s<0 \,\, \text {and} \,\, \frac{1}{4}\le b'<\frac{1}{2}, \end{aligned}$$

which we will prove later. Next, combining (3.56) with inequality (3.54) we obtain

3.57 $$\begin{aligned} \Theta _{3} \lesssim \frac{1}{ (1+|\tau +\xi ^{m}|)^{6b'+2s/m-2} } + \frac{1}{ (1+|\tau +\xi ^{m}|)^{6b'+(4s+m-1)/m-2} }. \end{aligned}$$

The first fraction $\frac{1}{ (1+|\tau +\xi ^{m}|)^{6b'+2s/m-2} }$ is bounded if $6b'+2\,s/m-2\ge 0$ or $b' \ge \frac{1}{3}-\frac{s}{3\,m}.$ Since $b'<\frac{1}{2}$, it suffices to have $-\frac{s}{3m}<1/6$ or $s>-\frac{1}{2}m=-j$. And, the second fraction $\frac{1}{ (1+|\tau +\xi ^{m}|)^{6b'+(4\,s+m-1)/m-2} }$ is bounded if $6b'+(4s+m-1)/m-2\ge 0$ or $b' \ge \frac{1}{3}-\frac{4\,s+m-1}{6\,m} = \frac{1}{6}-\frac{4\,s-1}{6\,m}.$ Since $b'<1/2$, it suffices to have $\frac{1}{6}-\frac{4s-1}{6m}<\frac{1}{2}$ or $4s-1>-2m$, or $s>-j+\frac{1}{4},$ which is the critical exponent $s_3$ for the nonlinearity $N_3$. This completes the proof of Lemma 3.4 once we prove inequality (3.56). $\square$

#### Proof of inequality (3.56)

In integral (3.55) we make the change of variables $$\mu =\mu (\xi _1)=\tau +\xi ^m-d_{3,m}(\xi ,\xi _1)$$. Using Proposition 3.1, we have that $$d_{3,m}(\xi ,\xi _1)$$ as a function of $$\xi _1$$ has the critical point $$\xi _1=\frac{1}{2}\xi $$. Thus, splitting the integral $$I_3$$ at $$\xi _1=\frac{1}{2}\xi $$, we get $$ I_{3} = I_3^+ + I_3^-, $$ where

3.58 $$\begin{aligned}&I_3^+(\xi ,\tau ) \doteq \int _{\frac{1}{2}\xi }^\infty \frac{ (1+|\xi |)^{2s} }{ |\xi _1(\xi -\xi _1)|^{2s} } \frac{ \chi _{E_{1}} (\xi _1,\tau _1,\xi ,\tau ) }{ (1+|\tau +\xi ^m-d_{3,m}(\xi ,\xi _1)|)^{4b'-1} } d\xi _1, \end{aligned}$$

3.59 $$\begin{aligned}&I_3^-(\xi ,\tau ) \doteq \int _{-\infty }^{\frac{1}{2}\xi } \frac{ (1+|\xi |)^{2s} }{ |\xi _1(\xi -\xi _1)|^{2s} } \frac{ \chi _{E_{1}} (\xi _1,\tau _1,\xi ,\tau ) }{ (1+|\tau +\xi ^m-d_{3,m}(\xi ,\xi _1)|)^{4b'-1} } d\xi _1. \end{aligned}$$

Since the estimation of $I_3^-$ is similar to that of $I_3^+$, here we only estimate $I_3^+$. To do this, we will need the following comparison property, which follows from inequalities (3.12) and (3.13)

3.60 $$\begin{aligned} |\tau +\xi ^m| \gtrsim d_{3,m}(\xi ,\xi _1) \ge \max \{\xi ^m,\xi _1^m,(\xi -\xi _1)^m\} > 1, \quad (\xi _1,\tau _1,\xi ,\tau )\in E_1. \nonumber \\ \end{aligned}$$

Furthermore, we consider the two cases: $\xi _1$ is near the critical point, and $\xi _1$ is away from the critical point. In the second case, we also compare the sizes of $|\xi |$ and $|\xi _1|$. More precisely, using this splitting we rewrite $I_3^+=I_{3,1}+I_{3,2}+I_{3,3}$, where

3.61 $$\begin{aligned} I_{3,1}(\xi ,\tau ) \doteq&\int _{\frac{1}{2}\xi }^{\frac{1}{2}\xi +1} \frac{ (1+|\xi |)^{2s} }{ |\xi _1(\xi -\xi _1)|^{2s} } \frac{ \chi _{E_{1}} (\xi _1,\tau _1,\xi ,\tau ) }{ (1+|\tau +\xi ^m-d_{3,m}(\xi ,\xi _1)|)^{4b'-1} } d\xi _1, \end{aligned}$$

3.62 $$\begin{aligned} I_{3,2}(\xi ,\tau ) \doteq&\int _{\frac{1}{2}\xi +1}^{10|\xi |+1} \frac{ (1+|\xi |)^{2s} }{ |\xi _1(\xi -\xi _1)|^{2s} } \frac{ \chi _{E_{1}} (\xi _1,\tau _1,\xi ,\tau ) }{ (1+|\tau +\xi ^m-d_{3,m}(\xi ,\xi _1)|)^{4b'-1} } d\xi _1, \end{aligned}$$

3.63 $$\begin{aligned} I_{3,3}(\xi ,\tau ) \doteq&\int _{10|\xi |+1}^\infty \frac{ (1+|\xi |)^{2s} }{ |\xi _1(\xi -\xi _1)|^{2s} } \frac{ \chi _{E_{1}} (\xi _1,\tau _1,\xi ,\tau ) }{ (1+|\tau +\xi ^m-d_{3,m}(\xi ,\xi _1)|)^{4b'-1} } d\xi _1. \end{aligned}$$

$\underline{\hbox {Estimate for }I_{3,1}.}$ Recall that for $(\xi _1,\tau _1,\xi ,\tau )\in E_1$, we have $|\xi _1|>10 \,\, \text {and} \,\, |\xi -\xi _1|>10$. Thus, if $\frac{1}{2}\xi <\xi _1\le \frac{1}{2}\xi +1$, then $|\xi _1|\simeq |\xi |$. This implies that $\chi _{\frac{1}{2}\xi <\xi _1\le \frac{1}{2}\xi +1}(\xi _1) \frac{ \chi _{E_{1}} (1+|\xi |)^{2\,s} }{ |\xi _1(\xi -\xi _1)|^{2\,s} } \lesssim \frac{ 1 }{ |\xi -\xi _1|^{2\,s} }.$ Furthermore, using inequality (3.60) we obtain

3.64 $$\begin{aligned} 1< |\xi -\xi _1| \lesssim |\tau +\xi ^m|^{1/m}, \quad (\xi _1,\tau _1,\xi ,\tau )\in E_1. \end{aligned}$$

Next, combining the above inequalities with definition (3.61), for $b'\ge \frac{1}{4}$ and $s<0$ we get

$$\begin{aligned} I_{3,1}(\xi ,\tau )&\lesssim \frac{1}{ (1+|\tau +\xi ^{m}|)^{2s/m} } \int _{\frac{1}{2}\xi }^{\frac{1}{2}\xi +1} \frac{ 1 }{ (1+|\tau +\xi ^m-d_{3,m}(\xi ,\xi _1)|)^{4b'-1} } d\xi _1 \\&\lesssim \frac{1}{ (1+|\tau +\xi ^{m}|)^{2s/m} }. \end{aligned}$$

This is the desired estimate (3.56) for $I_{3,1}$.

$\underline{\hbox {Estimate for }I_{3,2}.}$ Here, we make the change of variables $\mu =\mu (\xi _1)=\tau +\xi ^m-d_{3,m}(\xi ,\xi _1)$ or $\xi _1=\xi _1(\mu )$

3.65 $$\begin{aligned} I_{3,2}(\xi , \tau ) \lesssim \int ^{\mu (\frac{1}{2}\xi +1)}_{\mu (10|\xi |+1)} \frac{ (1+|\xi |)^{2s} }{ |\xi _1(\mu )(\xi -\xi _1(\mu ))|^{2s} } \frac{ \chi _{E_{1}} (\xi _1,\tau _1,\xi ,\tau ) }{ (1+|\mu |)^{4b'-1} } \frac{1}{|\partial _{\xi _1}\mu |} d\mu . \end{aligned}$$

Since $\partial _{\xi _1}\mu =-\partial _{\xi _1}d_{3,m}(\xi ,\xi _1)$, using the lower bound for $\partial _{\xi _1}d_{3,m}$, i.e., inequality (3.16), we get $|\partial _{\xi _1}\mu |\gtrsim |\xi _1-\frac{1}{2}\xi |^{m-1}\gtrsim 1$ for $\xi _1>\frac{1}{2}\xi +1$. Furthermore, for $|\xi _1|>10$, if $\frac{1}{2}\xi +1< \xi _1\le 10|\xi |+1$, then we have $|\xi _1|\lesssim |\xi |$, which gives us $\chi _{\frac{1}{2}\xi +1< \xi _1\le 10|\xi |+1}(\xi _1) \cdot \frac{ (1+|\xi |)^{2\,s} }{ |\xi _1(\xi -\xi _1)|^{2\,s} } \lesssim \frac{ 1 }{ |\xi -\xi _1|^{2\,s} }$ for $s<0$. Now, combining these inequalities with (3.64) and integrating over the whole $\mathbb {R}$, from (3.65) we obtain

3.66 $$\begin{aligned} I_{3,2}&\lesssim \int _{\mathbb {R}} \frac{ 1 }{ |\tau +\xi ^m|^{2s/m} } \frac{ \chi _{E_{1}} (\xi _1,\tau _1,\xi ,\tau ) }{ (1+|\mu |)^{4b'-1} } d\mu = \frac{ 1 }{ (1+|\tau +\xi ^m|)^{2s/m} } \int _{\mathbb {R}} \nonumber \\&\quad \frac{ \chi _{E_{1}} (\xi _1,\tau _1,\xi ,\tau ) }{ (1+|\mu |)^{4b'-1} } d\mu , \,\, s<0. \end{aligned}$$

Next, using triangle inequality and using inequality (3.60), we get $|\mu |\le |\tau +\xi ^m|+|d_{3,m}|\lesssim |\tau +\xi ^m|$ for $(\xi _1,\tau _1,\xi ,\tau ) \in E_1$. Therefore, for $4b'-1<1$ or $b'<\frac{1}{2}$ we have

3.67 $$\begin{aligned}  &   \int _{\mathbb {R}} \frac{ \chi _{E_{1}} (\xi _1,\tau _1,\xi ,\tau ) }{ (1+|\mu |)^{4b'-1} } d\mu \lesssim \int _{|\mu |\lesssim |\tau +\xi ^m|} \frac{ 1 }{ (1+|\mu |)^{4b'-1} } d\mu \lesssim (1+|\tau +\xi ^m|)^{2-4b'},\nonumber \\  &   \quad b'<\frac{1}{2}, \end{aligned}$$

which combined with inequality (3.66) implies that

3.68 $$\begin{aligned}&I_{3,2} \lesssim \frac{1}{(1+|\tau +\xi ^m|)^{2s/m}} (1+|\tau +\xi ^m|)^{2-4b'} = \frac{1}{(1+|\tau +\xi ^m|)^{4b'-2+2s/m}}, \nonumber \\&\quad s<0 \,\, \text {and} \,\, b'< \frac{1}{2}. \end{aligned}$$

This is the desired estimate (3.56) for $I_{3,2}$.

$\underline{\hbox {Estimate for }I_{3,3}.}$ Making the change of variables $\mu =\mu (\xi _1)=\tau +\xi ^m-d_{3,m}(\xi ,\xi _1)$ or $\xi _1=\xi _1(\mu )$, we get

3.69 $$\begin{aligned} I_{3,3}(\xi , \tau ) \lesssim \int ^{\mu (10|\xi |+1)}_{-\infty } \frac{ (1+|\xi |)^{2s} }{ |\xi _1(\mu )(\xi -\xi _1(\mu ))|^{2s} } \frac{ \chi _{E_{1}} (\xi _1,\tau _1,\xi ,\tau ) }{ (1+|\mu |)^{4b'-1} } \frac{1}{|\partial _{\xi _1}\mu |} d\mu . \end{aligned}$$

Since $\partial _{\xi _1}\mu =-\partial _{\xi _1}d_{3,m}(\xi ,\xi _1)$, using the lower bound for $\partial _{\xi _1}d_{3,m}$, i.e., inequality (3.16), we get $|\partial _{\xi _1}\mu |\gtrsim |\xi _1-\frac{1}{2}\xi |^{m-1}\gtrsim |\xi _1|^{m-1}$ for $\xi _1>10|\xi |+1$. In fact, for $\xi _1>10|\xi |+1>10|\xi |$, by the triangle inequality, we have $|\xi _1-\frac{1}{2}\xi |\ge |\xi _1|-\frac{1}{2}|\xi |\ge |\xi _1|-\frac{1}{20}|\xi _1|=\frac{19}{20}|\xi _1|$.

Furthermore, if

$\xi _1>10|\xi |$, then we have $|\xi -\xi _1| \le |\xi _1|+|\xi | \le |\xi _1|+\frac{1}{10}|\xi _1| \le \frac{11}{10}|\xi _1|,$ which gives us $\chi _{\xi _1>10|\xi |+1}(\xi _1) \cdot \frac{ (1+|\xi |)^{2\,s} }{ |\xi _1(\xi -\xi _1)|^{2\,s} } \lesssim \frac{ 1 }{ |\xi _1|^{4\,s} },$ for $s<0$. Combining these inequalities and integrating over the whole $\mathbb {R}$, from (3.69) we get

3.70 $$\begin{aligned} I_{3,3}(\xi ,\tau ) \lesssim \int ^{\mu (10|\xi |+1)}_{-\infty } \frac{ 1 }{ |\xi _1(\mu )|^{4s+m-1} } \frac{ \chi _{E_{1}} (\xi _1,\tau _1,\xi ,\tau ) }{ (1+|\mu |)^{4b'-1} } d\mu . \end{aligned}$$

Since $|\xi _1|>10$, we notice that if *s* is smaller, then the integral $I_{3,3}$ becomes bigger. Thus, here we assume that $4s+m-1\le 0$. Again, using inequality (3.60), we obtain $1< |\xi _1| \lesssim |\tau +\xi ^m|^{1/m},$ for $(\xi _1,\tau _1,\xi ,\tau ) \in E_{1}.$ Next, using these inequalities, from (3.70) we get

3.71 $$\begin{aligned}&I_{3,3} \lesssim \int _{\mathbb {R}} \frac{ \chi _{E_{1}} (\xi _1,\tau _1,\xi ,\tau ) }{ |\tau +\xi ^m|^{(4s+m-1)/m} } \frac{ d\mu }{ (1+|\mu |)^{4b'-1} } = \frac{1}{ (1+|\tau +\xi ^{m}|)^{(4s+m-1)/m} } \int _{\mathbb {R}}\nonumber \\&\quad \frac{ \chi _{E_{1}} (\xi _1,\tau _1,\xi ,\tau ) \, d\mu }{ (1+|\mu |)^{4b'-1} }, \end{aligned}$$

which combined with inequality (3.67) implies the desired estimate (3.56) for $I_{3,3}$. This completes the proof of inequality (3.56). $\square$

**Proof in Microlocalization II** Here, we have $|\tau _1-\xi _1^m|=\max \big \{ |\tau +\xi ^m|, |\tau _1-\xi _1^m|, \big | \tau -\tau _1-(\xi -\xi _1)^m \big | \big \}$. In this situation, the $L^2$ bilinear estimate (3.47) reads

3.72 $$\begin{aligned}&\Big \Vert \int _{\mathbb {R}^2} \chi _{E_2}(\xi _1,\tau _1,\xi ,\tau )\cdot Q_1(\xi _1,\tau _1,\xi ,\tau ) \cdot c_{f}(\xi -\xi _1,\tau -\tau _1) c_{g}(\xi _1,\tau _1) d\xi _1 d\tau _1 \Big \Vert _{L^2_{\xi ,\tau }}\nonumber \\&\quad \lesssim \Vert c_{f}\Vert _{L^2_{\xi ,\tau }} \Vert c_{g}\Vert _{L^2_{\xi ,\tau }}. \end{aligned}$$

Similar to inequality (3.39), utilizing duality and applying the Cauchy–Schwarz inequality twice, we have

3.73 $$\begin{aligned} \text {LHS of }{(3.72)} \lesssim \Big \Vert \int _{\mathbb {R}^2} \chi _{E_2}(\xi _1,\tau _1,\xi ,\tau )\cdot Q_1(\xi _1,\tau _1,\xi ,\tau ) d\xi d\tau \Big \Vert _{L^{\infty }_{\xi _1,\tau _1} }^{1/2} \cdot \Vert c_{f}\Vert _{L^2_{\xi ,\tau }} \Vert c_{g}\Vert _{L^2_{\xi ,\tau }}. \end{aligned}$$

Thus, to prove $L^2$ bilinear estimate (3.72), it suffices to show the following result.

#### Lemma 3.5

Let $m=2j\ge 2$ be an even number. If $-j+\frac{1}{4}<s<0$ and $\max \{\frac{1}{3}-\frac{s}{3\,m},\frac{1}{6}-\frac{4\,s-1}{6\,m}\}\le b'<\frac{1}{2}$, then for $\xi _1,\tau _1\in \mathbb {R}$, we have

3.74 $$\begin{aligned}&\Theta _{4} \doteq \frac{1}{ (1+|\tau _1-\xi _1^{m}|)^{2b'} } \int _{\mathbb {R}^2} \frac{ (1+|\xi |)^{2s} }{ |\xi _1(\xi -\xi _1)|^{2s} }\nonumber \\&\quad \frac{ \chi _{E_{2}} (\xi _1,\tau _1,\xi ,\tau ) }{ (1+|\tau -\tau _1-(\xi -\xi _1)^m|)^{2b'} (1+|\tau +\xi ^m|)^{2b'} } \, d\tau d\xi \lesssim 1. \end{aligned}$$

#### Proof of Lemma 3.5

For the $\tau$ integral in (3.74), applying calculus estimate (3.24) with $\ell =\ell '=b'>\frac{1}{4}$, $x=\tau _1$, $\alpha =\tau _1+(\xi -\xi _1)^m$, $\beta =-\xi ^m$, we get

3.75 $$\begin{aligned}&\Theta _{4} \lesssim \frac{1}{ (1+|\tau _1-\xi _1^{m}|)^{2b'} } \int _{\mathbb {R}} \frac{ (1+|\xi |)^{2s} }{ |\xi _1(\xi -\xi _1)|^{2s} } \nonumber \\&\quad \frac{ \chi _{E_{2}} (\xi _1,\tau _1,\xi ,\tau ) d\xi }{ (1+|\tau _1+(\xi -\xi _1)^m+\xi ^m|)^{4b'-1} } = \frac{1}{ (1+|\tau _1-\xi _1^{m}|)^{2b'} } I_4, \end{aligned}$$

where the integral $I_4$ is defined by

3.76 $$\begin{aligned} I_4(\xi _1, \tau _1) \doteq&\int _{\mathbb {R}} \frac{ (1+|\xi |)^{2s} }{ |\xi _1(\xi -\xi _1)|^{2s} } \frac{ \chi _{E_{2}} (\xi _1,\tau _1,\xi ,\tau ) }{ (1+|\tau _1-\xi _1^m+d_{3,m}(\xi ,\xi _1)|)^{4b'-1} } d\xi . \end{aligned}$$

Here, $d_{3,m}(\xi ,\xi _1)=\xi ^m+\xi _1^m+(\xi -\xi _1)^m$. Also, we can show the following bound for $I_4$

3.77 $$\begin{aligned}&I_4 \lesssim \frac{1}{ (1+|\tau _1-\xi ^{m}|)^{4b'+2s/m-2} } + \frac{1}{ (1+|\tau _1-\xi _1^{m}|)^{4b'+(4s+m-1)/m-2} } ,\nonumber \\&\quad s<0 \,\, \text {and} \,\, \frac{1}{4}\le b'<\frac{1}{2}. \end{aligned}$$

In fact, due to the symmetry of $d_{3,m}(\xi ,\xi _1)$ with respect to the variables $\xi$ and $\xi _1$, the proof of inequality (3.77) is similar to the proof of inequality (3.56) for the integral $I_3$. Finally, by combining inequality (3.77) with (3.75), we obtain

3.78 $$\begin{aligned} \Theta _{4} \lesssim \frac{1}{ (1+|\tau _1-\xi _1^{m}|)^{6b'+2s/m-2} } + \frac{1}{ (1+|\tau _1-\xi _1^{m}|)^{6b'+(4s+m-1)/m-2} }, \end{aligned}$$

which is similar to inequality (3.57) for $\Theta _3$. Therefore, following a similar argument used to derive the bound for $\Theta _3$, we can conclude that $\Theta _4$ is bounded when $-j+\frac{1}{4}<s<0$ and $\max \{\frac{1}{3}-\frac{s}{3\,m},\frac{1}{6}-\frac{4\,s-1}{6\,m}\}\le b'<\frac{1}{2}$. This completes the proof of Lemma 3.5. $\square$

### Optimality of $\bar{f}\bar{g}$ spatial bilinear estimate

We will prove the following result.

#### Lemma 3.6

If $s\le -j+\frac{1}{4}$, then bilinear estimate $\Vert \bar{f}\bar{g}\Vert _{X^{s,-b}} \lesssim \Vert f\Vert _{X^{s,b}} \Vert g\Vert _{X^{s,b}}$ fails for any $b<\frac{1}{2}$.

#### Proof of Lemma 3.6

It suffices to prove that if $s\le -j+\frac{1}{4}$, then $L^2$ bilinear estimate (3.7) fails for any $b=b'<\frac{1}{2}$. To prove this, for fix $N\in \mathbb {Z}^+$, we define the $L^2$ functions $c_{f}, c_{g}$ as follows

3.79 $$\begin{aligned} c_{f}(\xi _2,\tau _2) = \chi _{A}(\xi _2,\tau _2) \in L^2_{\xi _2,\tau _2}, \qquad c_{g}(\xi _1,\tau _1) = \chi _{\tilde{A}^+}(\xi _1,\tau _1) \in L^2_{\xi _1,\tau _1}, \end{aligned}$$

where $\chi _{A}(\cdot )$ is the characteristic function of the region *A*, which is defined by

3.80 $$\begin{aligned} A \doteq \Big \{ (\xi _2,\tau _2) \in \mathbb {R}^2 : -N-\frac{10}{N^{\frac{m-2}{2}}} \le \xi _2 \le -N+\frac{10}{N^{\frac{m-2}{2}}}, \,\, |\tau _2+\xi _2^m| \le 10^m(m+1)! \Big \}, \end{aligned}$$

and $\chi _{\tilde{A}^+}(\cdot )$ is the characteristic function of the region $\tilde{A}^+$, which is defined by

3.81 $$\begin{aligned} \tilde{A}^+ \doteq \Big \{ (\xi _1,\tau _1) \in \mathbb {R}^2 : N-\frac{1}{N^{\frac{m-2}{2}}} \le \xi _1 \le N+\frac{1}{N^{\frac{m-2}{2}}}, \quad |\tau _1-\xi _1^m| \le (m+1)! \Big \}. \end{aligned}$$

Also, it is useful to define the region $\tilde{A}^-\doteq \Big \{ (\xi _1,\tau _1) \in \mathbb {R}^2: (-\xi _1,-\tau _1) \in \tilde{A}^+ \Big \}$. By definition of *A* and $\tilde{A}^-$, we get $\tilde{A}^-\subset A$. Furthermore, by a straightforward computation we get

3.82 $$\begin{aligned} \Vert c_{f}\Vert _{L^2}^2 = \int _{\mathbb {R}^2} \chi _{A}(\xi _2,\tau _2) d\xi _2d\tau _2 \simeq \frac{1}{N^{\frac{m-2}{2}}}, \quad \text {and} \quad \Vert c_{g}\Vert _{L^2}^2 \simeq \frac{1}{N^{\frac{m-2}{2}}}. \end{aligned}$$

Next, we define the integrand function $\Theta (\xi ,\tau )$ in $L^2$ bilinear estimate (3.7)

3.83 $$\begin{aligned}&\Theta \doteq \frac{(1+|\xi |)^s}{(1+|\tau +\xi ^m|)^{b}} \int _{\mathbb {R}^2}\nonumber \\&\quad \frac{c_{f}(\xi -\xi _1,\tau -\tau _1) c_{g}(\xi _1,\tau _1) \,d\xi _1 d\tau _1 }{(1+|\xi -\xi _1|)^s(1+|\tau -\tau _1-(\xi -\xi _1)^m|)^{b}(1+|\xi _1|)^s(1+|\tau _1-\xi _1^m|)^{b}}, \end{aligned}$$

and we estimate its $L^2$ norm. Now, we claim the following key estimate for $\Theta$

3.84 $$\begin{aligned} \Vert \Theta \Vert _{L^2_{\xi ,\tau }} \gtrsim \frac{1}{N^{2s+\frac{3}{2}mb+\frac{3}{4}(m-2)}}, \end{aligned}$$

which we will prove later. Next, using inequalities (3.84) and (3.82) we see that if $L^2$ bilinear estimate (3.7) holds, then we must have $\frac{1}{N^{2\,s+\frac{3}{2}mb+\frac{3}{4}(m-2)}} \lesssim \frac{1}{N^{\frac{m-2}{2}}}$ or $N^{-2\,s} \lesssim N^{\frac{3}{2}mb+\frac{1}{4}(m-2)}.$ Since $N\gg 1$, for this to be true we need $-2\,s\le \frac{3}{2}mb+\frac{1}{4}(m-2)$ or $b \ge -\frac{4}{3\,m}s-\frac{1}{6}+\frac{1}{3\,m}.$ Also, since $b<\frac{1}{2}$, we must have $-\frac{4}{3m}s-\frac{1}{6}+\frac{1}{3m}<\frac{1}{2}$ or

3.85 $$\begin{aligned} s > -\frac{1}{2}m+\frac{1}{4} = -j+\frac{1}{4}. \end{aligned}$$

This completes the proof of Lemma 3.6 once we prove inequality (3.84). $\square$

**Proof of inequality** (3.84). Following [19, 45], we can show that there is a rectangle with dimension $c\frac{1}{N^{m-1}}\times N^{\frac{m}{2}}$, where *c* is a constant depending on *m*, in the domain $\tilde{A}^+$. Also, there is a similar rectangle centered at the origin that we denote it by $\Delta$. Furthermore, we can prove that if $(\xi ,\tau )\in \Delta$ and $(\xi _1,\tau _1)\in \tilde{A}^+$, then $(\xi -\xi _1,\tau -\tau _1)\in \tilde{A}^-\subset A$. Thus, if $(\xi ,\tau )\in \Delta$ and $(\xi _1,\tau _1)\in \tilde{A}^+$, then

3.86 $$\begin{aligned}&|\tau +\xi ^m| \ge |\tau |-\xi ^m \gtrsim N^{\frac{1}{2}m}, \end{aligned}$$

3.87 $$\begin{aligned}&|\tau -\tau _1-(\xi -\xi _1)^m| = |\tau -\tau _1+(\xi -\xi _1)^m-2(\xi -\xi _1)^m| \simeq 2(\xi -\xi _1)^m \simeq N^m. \end{aligned}$$

Now, we can estimate the $L^2$ norm of the integrand $\Theta$, which is defined by (3.83). In fact, restricting to the regions $(\xi ,\tau )\in \Delta$ and $(\xi _1,\tau _1)\in \tilde{A}^+$, we obtain the following estimate

3.88 $$\begin{aligned} \Vert \Theta \Vert _{L^2_{\xi ,\tau }}&\ge \Big \Vert \frac{\chi _{\Delta }(\xi ,\tau )(1+|\xi |)^s}{(1+|\tau +\xi ^m|)^{b}} \int _{\mathbb {R}^2}\nonumber \\&\quad \frac{\chi _{\tilde{A}^+}(\xi _1,\tau _1) \cdot c_{f}(\xi -\xi _1,\tau -\tau _1) c_{g}(\xi _1,\tau _1)\,d\xi _1 d\tau _1}{(1+|\xi -\xi _1|)^s(1+|\tau -\tau _1+(\xi -\xi _1)^m|)^{b}(1+|\xi _1|)^s(1+|\tau _1-\xi _1^m|)^{b}} \Big \Vert _{L^2_{\xi ,\tau }} \nonumber \\&\ge \Big \Vert \frac{\chi _{\Delta }(\xi ,\tau )}{N^{\frac{1}{2}mb}} \cdot \int _{\mathbb {R}^2} \frac{\chi _{\tilde{A}^+}(\xi _1,\tau _1)}{N^{2s}N^{mb}} d\xi _1 d\tau _1 \Big \Vert _{L^2_{\xi ,\tau }} \nonumber \\&\quad = \frac{1}{N^{2s+\frac{3}{2}mb}} \cdot \int _{\mathbb {R}^2} \chi _{\tilde{A}^+}(\xi _1,\tau _1) d\xi _1 d\tau _1 \cdot \big \Vert \chi _{\Delta }(\cdot ,\cdot ) \big \Vert _{L^2_{\xi ,\tau }} \nonumber \\&\simeq \frac{1}{N^{2s+\frac{3}{2}mb}} \cdot \frac{1}{N^{\frac{1}{2}(m-2)}} \cdot \frac{1}{N^{\frac{1}{4}(m-2)}} = \frac{1}{N^{2s+\frac{1}{2}mb+\frac{3}{4}(m-2)}}. \end{aligned}$$

This completes the proof of inequality (3.84). $\square$

## *fg* bilinear estimates in spatial Bourgain spaces

In this section, we prove the spatial bilinear estimates for *fg* and demonstrate its optimality.

### *fg* spatial bilinear estimates

Following the notation used in $\bar{f}\bar{g}$ spatial bilinear estimates (inequality (3.3)), the proof of *fg* spatial bilinear estimate is reduced to the proof of its $L^2$ formulation

4.1 $$\begin{aligned} \Big \Vert \int _{\mathbb {R}^2} Q(\xi _1,\tau _1,\xi ,\tau ) \cdot c_{f}(\xi -\xi _1,\tau -\tau _1) c_{g}(\xi _1,\tau _1) d\xi _1 d\tau _1 \Big \Vert _{L^2_{\xi ,\tau }} \lesssim \Vert c_{f}\Vert _{L^2_{\xi ,\tau }} \Vert c_{g}\Vert _{L^2_{\xi ,\tau }}, \end{aligned}$$

where the multiplier *Q* is defined as follows

4.2 $$\begin{aligned} Q&\doteq \frac{ (1+|\xi |)^s }{ (1+|\xi _1|)^{s} (1+|\xi -\xi _1|)^{s} } \cdot \frac{1}{(1+|\tau +\xi ^m|)^{b}} \nonumber \\&\quad \cdot \frac{1}{ (1+|\tau _1+\xi _{1}^{m}|)^{b'} (1+|\tau -\tau _1+(\xi -\xi _1)^m|)^{b'} }. \end{aligned}$$

Also, using the symmetry of convolution writing for $\widehat{f} * \widehat{g}$ we can assume that $|\tau -\tau _1+(\xi -\xi _1)^m| \le |\tau _1+\xi _1^m|.$ Furthermore, utilizing $b' \le b$, the $L^2$ inequality (4.1) is reduced to the following $L^2$ inequality

4.3 $$\begin{aligned} \Big \Vert \int _{\mathbb {R}^2} (\chi _{A} Q') (\xi _1,\tau _1,\xi ,\tau ) \cdot c_{f}(\xi -\xi _1,\tau -\tau _1) c_{g}(\xi _1,\tau _1) d\xi _1 d\tau _1 \Big \Vert _{L^2_{\xi ,\tau }} \lesssim \Vert c_{f}\Vert _{L^2_{\xi ,\tau }} \Vert c_{g}\Vert _{L^2_{\xi ,\tau }}, \end{aligned}$$

where the region $A\subset \mathbb {R}^4$ is defined as follows

4.4 $$\begin{aligned} A \doteq \{ (\xi _1,\tau _1,\xi ,\tau )\in \mathbb {R}^4: |\tau -\tau _1+(\xi -\xi _1)^m| \le |\tau _1+\xi _1^m| \}, \end{aligned}$$

and the multiplier $Q'$ is defined by

4.5 $$\begin{aligned}&Q' \doteq \frac{ (1+|\xi |)^s }{ (1+|\xi _1|)^{s} (1+|\xi -\xi _1|)^{s} } \cdot \frac{1}{(1+|\tau +\xi ^m|)^{b'}} \nonumber \\&\quad \cdot \frac{1}{ (1+|\tau _1+\xi _{1}^{m}|)^{b'} (1+|\tau -\tau _1+(\xi -\xi _1)^m|)^{b'} }. \end{aligned}$$

From the multiplier $Q'$, we recognize the familiar Bourgain quantity for the nonlinearity *fg*

4.6 $$\begin{aligned} d_{1,m}(\xi ,\xi _1) \doteq \xi ^m-\xi _1^m-(\xi -\xi _1)^m = (\tau +\xi ^m)-(\tau _1+\xi _1^m)-[(\tau -\tau _1)+(\xi -\xi _1)^m].\nonumber \\ \end{aligned}$$

We list the basic properties of $d_{1,m}$ in the following result, whose proof is similar to the one for $d_{3,m}$.

#### Proposition 4.1

If $m=2j\ge 2$ is an even number, then $d_{1,m}(\xi ,\xi _1)$ a homogeneous polynomial of $\xi$ and $\xi _1$ of degree *m*, and has the following properties:

4.7 $$\begin{aligned}  &   \bullet \text { Comparison Property:} \qquad \max \{ |\tau +\xi ^m|, |\tau _1+\xi _1^m|, |\tau -\tau _1+(\xi -\xi _1)^m| \} \nonumber \\  &   \ge \frac{1}{3} |d_{1,m}(\xi ,\xi _1)|. \end{aligned}$$

4.8 $$\begin{aligned}  &   \bullet \,\, |d_{1,m}(\xi ,\xi _1)| \gtrsim |\xi -\xi _1||\xi _1| (\xi ^{m-2}+\xi _1^{m-2}) \quad \text {and} \quad |d_{1,m}(\xi ,\xi _1)|\nonumber \\  &   \gtrsim |\xi _1(\xi -\xi _1)|^{\frac{1}{2}m}. \end{aligned}$$

4.9 $$\begin{aligned}  &   \bullet \,\, \partial _\xi d_{1,m}(\xi ,\xi _1) = m\xi ^{m-1}-m(\xi -\xi _1)^{m-1} \quad \text {and} \quad \partial _{\xi _1} d_{1,m}(\xi ,\xi _1)\nonumber \\  &   = -[m\xi _1^{m-1}+m(\xi _1-\xi )^{m-1}]. \end{aligned}$$

4.10 $$\begin{aligned}  &   \bullet \text { Lower bound for }\partial _{\xi } d_{1,m}(\xi ,\xi _1): \quad \,\, |\partial _\xi d_{1,m}(\xi ,\xi _1)| \nonumber \\  &   \gtrsim \max \big \{ |\xi _1| (\xi -\frac{1}{2}\xi _1)^{m-2}, |\xi _1|^{m-1} \big \}. \end{aligned}$$

4.11 $$\begin{aligned}  &   \bullet \text { Lower bound for }\partial _{\xi _1} d_{1,m}(\xi ,\xi _1): \quad |\partial _{\xi _1} d_{1,m}(\xi ,\xi _1)|\nonumber \\  &   \gtrsim \max \big \{ |\xi _1-\frac{1}{2}\xi |^{m-1}, |\xi _1-\frac{1}{2}\xi |\xi ^{m-2} \big \}. \end{aligned}$$

$\bullet$ Monotonicity: When considering $d_{1,m}(\xi ,\xi _1)$ as a function of $\xi$, it exhibits an increasing monotonicity when $\xi _1>0$ and a decreasing monotonicity when $\xi _1<0$.

$\bullet$ Critical points: When considering $d_{1,m}(\xi ,\xi _1)$ as a function of $\xi _1$, we can determine that its critical point occurs at $\xi _1=\frac{1}{2}\xi$.

$\bullet$ Zeros of $d_{1,m}(\xi ,\xi _1)$: When considering $d_{1,m}(\xi ,\xi _1)$ as a function of $\xi$, it has zeros at $\xi =\xi _1$. When considering $d_{1,m}(\xi ,\xi _1)$ as a function of $\xi _1$, it has zeros at $\xi _1=0$ and $\xi _1=\xi$.

**Fig. 8:**
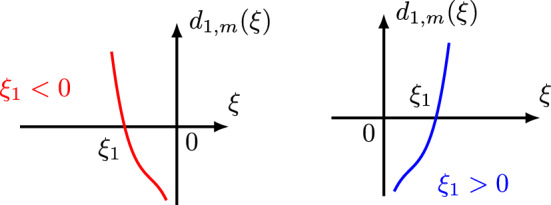
$d_{1,m}$ is a function of $\xi$

**Fig. 9:**
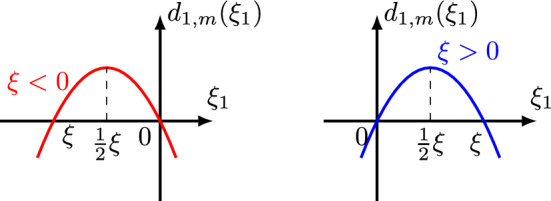
$d_{1,m}$ is a function of $\xi _1$

$\bullet$ Graphs: Figs. 8 and 9 display the graph of $d_{1,m}(\xi ,\xi _1)$ as a function of $\xi$ and $\xi _1$, respectively. $\square$

Next, we provide the proof of $L^2$ bilinear estimate (3.9) for the following two cases:

$$\bullet $$ Case: $$s\ge 0$$       $$\bullet $$ Case: $$s< 0$$.

**Bilinear estimate for** $s\ge 0$ Then, $(1+|\xi |)^s \le (1+|\xi -\xi _1|)^s (1+|\xi _1|)^s$, which implies $Q'\le Q_0$, where

4.12 $$\begin{aligned} Q_0(\xi _1,\tau _1,\xi ,\tau ) \doteq \frac{1}{(1+|\tau +\xi ^m|)^{b'}} \frac{1}{ (1+|\tau _1+\xi _{1}^{m}|)^{b'} (1+|\tau -\tau _1+(\xi -\xi _1)^m|)^{b'} }, \end{aligned}$$

Therefore, when $s\ge 0$, to prove $L^2$ bilinear estimate (4.3), it suffices to show that

4.13 $$\begin{aligned} \Big \Vert \int _{\mathbb {R}^2} (\chi _{A} Q_0)(\xi _1,\tau _1,\xi ,\tau ) \cdot c_{f}(\xi -\xi _1,\tau -\tau _1) c_{g}(\xi _1,\tau _1) d\xi _1 d\tau _1 \Big \Vert _{L^2_{\xi ,\tau }} \lesssim \Vert c_{f}\Vert _{L^2_{\xi ,\tau }} \Vert c_{g}\Vert _{L^2_{\xi ,\tau }}. \end{aligned}$$

To establish this, we consider the following familiar microlocalizations:

$\bullet$ **Microlocalization I**. Here, the region $A_{I}$ is defined as follows

4.14 $$\begin{aligned} A_{I} \doteq \big \{ (\xi _1,\tau _1,\xi ,\tau ) \in \mathbb {R}^4: |\xi _1| \le 1 \big \}. \end{aligned}$$

$\bullet$ **Microlocalization II**. It is defined by the region

4.15 $$\begin{aligned} A_{II} \doteq \big \{ (\xi _1,\tau _1,\xi ,\tau ) \in \mathbb {R}^4: |\xi _1|>1 \big \}. \end{aligned}$$

**Proof in Microlocalization I** ($|\xi _1|\le 1$). the $L^2$ bilinear estimate (4.13) reads

4.16 $$\begin{aligned} \Big \Vert \int _{\mathbb {R}^2} (\chi _{A_{I}}Q_0)(\xi _1,\tau _1,\xi ,\tau ) \cdot c_{f}(\xi -\xi _1,\tau -\tau _1) c_{g}(\xi _1,\tau _1) d\xi _1 d\tau _1 \Big \Vert _{L^2_{\xi ,\tau }} \lesssim \Vert c_{f}\Vert _{L^2_{\xi ,\tau }} \Vert c_{g}\Vert _{L^2_{\xi ,\tau }}. \end{aligned}$$

Working similarly to (3.22), applying Cauchy–Schwarz inequality with respect to $\xi _1$ and $\tau _1$, we get

4.17 $$\begin{aligned} \text {LHS of }{(4.16)} \lesssim \Big \Vert \int _{\mathbb {R}^2} (\chi _{A_{I}} Q_0^2) (\xi _1,\tau _1,\xi ,\tau ) \, d\xi _1 d\tau _1 \Big \Vert _{L^{\infty }_{\xi ,\tau } }^{1/2} \cdot \Vert c_{f}\Vert _{L^2_{\xi ,\tau }} \Vert c_{g}\Vert _{L^2_{\xi ,\tau }}. \end{aligned}$$

Thus, to prove $L^2$ bilinear estimate (4.16), it suffices to show the following result.

#### Lemma 4.1

Let $m=2j\ge 2$ be an even number. If $\frac{1}{4}<b'<\frac{1}{2}$, then for $\xi ,\tau \in \mathbb {R}$, we have

4.18 $$\begin{aligned}&\Theta _{0} (\xi , \tau ) \doteq \frac{1}{ (1+|\tau +\xi ^{m}|)^{2b'} } \int _{\mathbb {R}^2} \nonumber \\  &\quad \frac{ \chi _{A_{I}} (\xi _1,\tau _1,\xi ,\tau ) }{ (1+|\tau -\tau _1+(\xi -\xi _1)^m|)^{2b'} (1+|\tau _1+\xi _1^m|)^{2b'} } \, d\tau _1 d\xi _1 \lesssim 1. \end{aligned}$$

#### Proof of Lemma 4.1

For the $\tau _1$ integral in (4.18), applying calculus estimate (3.24) with $\ell =\ell '=b'>\frac{1}{4}$, $x=\tau _1$, $\alpha =\tau +(\xi -\xi _1)^m$, $\beta =-\xi _1^m$, we get

4.19 $$\begin{aligned} \Theta _{0} \lesssim&\frac{1}{ (1+|\tau +\xi ^{m}|)^{2b'} } \int _{|\xi _1|\le 1} \frac{ d\xi _1 }{ (1+|\tau +(\xi -\xi _1)^m+\xi _1^m|)^{4b'-1} } \nonumber \\\lesssim&\frac{1}{ (1+|\tau +\xi ^{m}|)^{2b'} } \lesssim 1, \quad b'> \frac{1}{4}. \end{aligned}$$

This completes the proof of Lemma 4.1. $\square$

#### Proof in Microlocalization II

($|\xi _1|>1$). Here, we consider the following two sub-microlocalizations:

$\bullet$ **Microlocalization II-1**. The region $A_{1}$ is defined as follows

4.20 $$\begin{aligned} A_{1} \doteq \big \{ (\xi _1,\tau _1,\xi ,\tau ) \in \mathbb {R}^4: |\tau -\tau _1+(\xi -\xi _1)^m| \le |\tau _1+\xi _1^m| \le |\tau +\xi ^{m}| \,\, \text {and} \,\, |\xi _1|>1 \big \}. \end{aligned}$$

$\bullet$ **Microlocalization II-2**. It is defined by the region

4.21 $$\begin{aligned} A_{2} \doteq \big \{ (\xi _1,\tau _1,\xi ,\tau ) \in \mathbb {R}^4: |\tau -\tau _1+(\xi -\xi _1)^m| \le |\tau _1+\xi _1^m|, \,\, |\tau +\xi ^{m}|< |\tau _1+\xi _1^m| \,\, \text {and} \,\, |\xi _1|>1 \big \}. \end{aligned}$$

**Proof in Microlocalization II-1.** Here, we have

$|\tau +\xi ^m|=\max \big \{ |\tau +\xi ^m|, |\tau _1+\xi _1^m|, \big | \tau -\tau _1+(\xi -\xi _1)^m \big | \big \}$. The $L^2$ bilinear estimate (4.13) in this situation reads

4.22 $$\begin{aligned} \Big \Vert \int _{\mathbb {R}^2} (\chi _{A_{1}}Q_0)(\xi _1,\tau _1,\xi ,\tau ) \cdot c_{f}(\xi -\xi _1,\tau -\tau _1) c_{g}(\xi _1,\tau _1) d\xi _1 d\tau _1 \Big \Vert _{L^2_{\xi ,\tau }} \lesssim \Vert c_{f}\Vert _{L^2_{\xi ,\tau }} \Vert c_{g}\Vert _{L^2_{\xi ,\tau }}. \end{aligned}$$

Again, applying Cauchy–Schwarz inequality in $\xi _1$ and $\tau _1$, we obtain

4.23 $$\begin{aligned} \text {LHS of }{(4.22)} \lesssim \Big \Vert \int _{\mathbb {R}^2} (\chi _{A_{1}} Q_0^2) (\xi _1,\tau _1,\xi ,\tau ) d\xi _1 d\tau _1 \Big \Vert _{L^{\infty }_{\xi ,\tau } }^{1/2} \cdot \Vert c_{f}\Vert _{L^2_{\xi ,\tau }} \Vert c_{g}\Vert _{L^2_{\xi ,\tau }}. \end{aligned}$$

Thus, to prove $L^2$ bilinear estimate (4.22), it suffices to show the following result. $\square$

#### Lemma 4.2

Let $m=2j\ge 2$ be an even number. If $\frac{1}{3}\le b'<\frac{1}{2}$, then for $\xi ,\tau \in \mathbb {R}$, we have

4.24 $$\begin{aligned}&\Theta _{1} (\xi , \tau ) \doteq \frac{1}{ (1+|\tau +\xi ^{m}|)^{2b'} } \int _{\mathbb {R}^2}\nonumber \\&\quad \frac{ \chi _{A_{1}} (\xi _1,\tau _1,\xi ,\tau ) }{ (1+|\tau -\tau _1+(\xi -\xi _1)^m|)^{2b'} (1+|\tau _1+\xi _1^m|)^{2b'} } \, d\tau _1 d\xi _1 \lesssim 1. \end{aligned}$$

#### Proof of Lemma 4.2

For the $\tau _1$ integral in (4.24), applying calculus estimate (3.24) with $\ell =\ell '=b'>\frac{1}{4}$, $x=\tau _1$, $\alpha =\tau +(\xi -\xi _1)^m$, $\beta =-\xi _1^m$, we get

4.25 $$\begin{aligned} \Theta _{1}(\xi , \tau ) \lesssim&\frac{1}{ (1+|\tau +\xi ^{m}|)^{2b'} } \int _{\mathbb {R}} \frac{ \chi _{A_{1}} (\xi _1,\tau _1,\xi ,\tau ) }{ (1+|\tau +(\xi -\xi _1)^m+\xi _1^m|)^{4b'-1} } d\xi _1 \nonumber \\ =&\frac{1}{ (1+|\tau +\xi ^{m}|)^{2b'} } I_1(\xi ,\tau ), \end{aligned}$$

where the integral $I_1$ is defined by

4.26 $$\begin{aligned} I_1(\xi ,\tau )&\doteq \int _{\mathbb {R}} \frac{ \chi _{A_{1}} (\xi _1,\tau _1,\xi ,\tau ) }{ (1+|\tau +(\xi -\xi _1)^m+\xi _1^m|)^{4b'-1} } d\xi _1 \nonumber \\&= \int _{\mathbb {R}} \frac{ \chi _{A_{1}} (\xi _1,\tau _1,\xi ,\tau ) }{ (1+|\tau +\xi ^m-d_{1,m}(\xi ,\xi _1)|)^{4b'-1} } d\xi _1. \end{aligned}$$

Here, $d_{1,m}(\xi ,\xi _1)=\xi ^m-\xi _1^m-(\xi -\xi _1)^m$ is the Bourgain quantity for nonlinearity *fg* and it is defined by (4.6). Also, we can bound the integral $I_1$ as follows

4.27 $$\begin{aligned} I_1(\xi ,\tau ) \lesssim (1+|\tau +\xi ^m|)^{2-4b'}, \quad \frac{1}{4}\le b'<\frac{1}{2}. \end{aligned}$$

In fact, since $d_{1,m}(\xi ,\xi _1)=-d_{3,m}(\xi ,\xi _1)+2\xi ^m$, when considering $d_{1,m}(\xi ,\xi _1)$ as a function of $\xi _1$, its behavior is similar to the behavior of $d_{3,m}(\xi ,\xi _1)$. Hence, the proof of inequality (4.27) is similar to the proof of inequality (3.27). Next, combining inequality (4.27) with (4.25), we obtain

4.28 $$\begin{aligned} \Theta _{1}(\xi , \tau ) \lesssim \frac{1}{(1+|\tau +\xi ^m|)^{2b'}} (1+|\tau +\xi ^m|)^{2-4b'} = \frac{1}{(1+|\tau +\xi ^m|)^{6b'-2}}, \end{aligned}$$

which is bounded if $6b'-2\ge 0$ or $b'\ge \frac{1}{3}$. This completes the proof of Lemma 4.2. $\square$

**Proof in Microlocalization II-2** Here, we have $|\tau _1+\xi _1^m|=\max \big \{ |\tau +\xi ^m|, |\tau _1+\xi _1^m|, \big | \tau -\tau _1+(\xi -\xi _1)^m \big | \big \}$. The $L^2$ bilinear estimate (4.13) reads

4.29 $$\begin{aligned}&\Big \Vert \int _{\mathbb {R}^2} \chi _{E_{1}} (\xi _1,\tau _1,\xi ,\tau ) \cdot Q_1 (\xi _1,\tau _1,\xi ,\tau ) \cdot c_{f}(\xi -\xi _1,\tau -\tau _1) c_{g}(\xi _1,\tau _1) d\xi _1 d\tau _1 \Big \Vert _{L^2_{\xi ,\tau }} \nonumber \\  &\quad \lesssim \Vert c_{f}\Vert _{L^2_{\xi ,\tau }} \Vert c_{g}\Vert _{L^2_{\xi ,\tau }}. \end{aligned}$$

Working similarly to inequality (3.39), by applying duality and employing the Cauchy–Schwarz inequality twice, the left-hand side of inequality (4.29) is bounded as follows

4.30 $$\begin{aligned} \text {LHS of }{(4.29)} \le \Vert c_f\Vert _{L^2_{\xi ,\tau }} \Vert c_g\Vert _{L^2_{\xi ,\tau }} \cdot \Big \Vert \int _{\mathbb {R}^2} (\chi _{A_2} Q_0^2)(\xi _1,\tau _1,\xi ,\tau ) d\xi d\tau \Big \Vert ^{\frac{1}{2}}_{L_{\xi _1,\tau _1}^\infty }. \end{aligned}$$

Thus, to prove $L^2$ bilinear estimate (4.29), it suffices to show the following result.

#### Lemma 4.3

Let $m=2j\ge 2$ be an even number. If $\frac{1}{3}\le b'<\frac{1}{2}$, then for $\xi _1,\tau _1\in \mathbb {R}$, we have

4.31 $$\begin{aligned}&\Theta _{2} (\xi _1, \tau _1) \doteq \frac{1}{ (1+|\tau _1+\xi _1^{m}|)^{2b'} } \nonumber \\&\quad \int _{\mathbb {R}^2} \frac{ \chi _{A_{2}} (\xi _1,\tau _1,\xi ,\tau ) }{ (1+|\tau -\tau _1+(\xi -\xi _1)^m|)^{2b'} (1+|\tau +\xi ^m|)^{2b'} } \, d\tau d\xi \lesssim 1. \end{aligned}$$

#### Proof of Lemma 4.3

For the $\tau$ integral in (4.31), applying calculus estimate (3.24) with $\ell =\ell '=b'>\frac{1}{4}$, $x=\tau _1$, $\alpha =\tau _1-(\xi -\xi _1)^m$, $\beta =-\xi ^m$, we get

4.32 $$\begin{aligned}&\Theta _{3} \lesssim \frac{1}{ (1+|\tau +\xi ^{m}|)^{2b'} } \int _{\mathbb {R}} \frac{ (1+|\xi |)^{2s} }{ |\xi _1(\xi -\xi _1)|^{2s} } \frac{ \chi _{E_{1}} (\xi _1,\tau _1,\xi ,\tau ) \, d\xi _1 }{ (1+|\tau +(\xi -\xi _1)^m+\xi _1^m|)^{4b'-1} } \nonumber \\  &\quad = \frac{1}{ (1+|\tau +\xi ^{m}|)^{2b'} } I_3, \end{aligned}$$

where the integral $I_2$ is defined by

4.33 $$\begin{aligned} I_2(\xi _1,\tau _1)&\doteq \int _{\mathbb {R}} \frac{ \chi _{A_{2}} (\xi _1,\tau _1,\xi ,\tau ) }{ (1+|\tau _1-(\xi -\xi _1)^m+\xi ^m|)^{4b'-1} } d\xi \nonumber \\&= \int _{\mathbb {R}} \frac{ \chi _{A_{2}} (\xi _1,\tau _1,\xi ,\tau ) }{ (1+|\tau _1+\xi _1^m+d_{1,m}(\xi ,\xi _1)|)^{4b'-1} } d\xi . \end{aligned}$$

Here, $d_{1,m}(\xi ,\xi _1)=\xi ^m-\xi _1^m-(\xi -\xi _1)^m$. Also, we will prove the following estimate for $I_2$ later

4.34 $$\begin{aligned} I_2(\xi _1,\tau _1) \lesssim (1+|\tau _1+\xi _1^m|)^{2-4b'}, \quad \frac{1}{4}\le b'<\frac{1}{2}. \end{aligned}$$

Next, combining inequality (4.34) with (4.32), we obtain

4.35 $$\begin{aligned} \Theta _{2}(\xi _1, \tau _1) \lesssim \frac{1}{(1+|\tau _1+\xi _1^m|)^{2b'}} (1+|\tau _1+\xi _1^m|)^{2-4b'} = \frac{1}{(1+|\tau _1+\xi _1^m|)^{6b'-2}}, \end{aligned}$$

which is bounded if $6b'-2\ge 0$ or $b'\ge \frac{1}{3}$. This completes the proof of Lemma 4.3. $\square$

**Proof of inequality **(4.34) We note that by Proposition 4.1, $d_{1,m}(\xi ,\xi _1)$ as a function of $\xi$ is increasing when $\xi _1>0$, and is decreasing when $\xi _1<0$. Therefore, we can make the change of variables $\mu =\mu (\xi )=\tau _1+\xi _1^m+d_{1,m}(\xi ,\xi _1)$ on $\mathbb {R}$ and we get

4.36 $$\begin{aligned} I_2(\xi _1,\tau _1) \lesssim \int _{\mathbb {R}} \frac{ \chi _{A_{2}} (\xi _1,\tau _1,\xi ,\tau ) }{ (1+|\mu |)^{4b'-1} } \frac{1}{|\partial _{\xi }\mu |} d\mu . \end{aligned}$$

Since $\partial _{\xi }\mu =\partial _{\xi }d_{1,m}(\xi ,\xi _1)$, using inequality (4.10), for $|\xi _1|>1$ we get $|\partial _{\xi }\mu |\gtrsim |\xi _1|^{m-1}\gtrsim 1$. Also, using comparison inequality (4.7), we get

4.37 $$\begin{aligned}  &   |\tau _1+\xi _1^m| = \max \big \{ |\tau +\xi ^m|, |\tau _1+\xi _1^m|, \big | \tau -\tau _1+(\xi -\xi _1)^m \big | \big \} \ge \frac{1}{3} d_{1,m}(\xi ,\xi _1),\nonumber \\  &   \quad (\xi _1,\tau _1,\xi ,\tau )\in A_2, \end{aligned}$$

which gives $|\mu |\le |\tau _1+\xi _1^m|+|d_{1,m}|\lesssim |\tau _1+\xi _1^m|$ for $(\xi _1,\tau _1,\xi ,\tau ) \in A_2$. Therefore, from (4.36) we get

4.38 $$\begin{aligned} I_2(\xi _1, \tau _1) \lesssim \int _{|\mu |\lesssim |\tau _1+\xi _1^m|} \frac{ 1 }{ (1+|\mu |)^{4b'-1} } d\mu \lesssim (1+|\tau _1+\xi _1^m|)^{2-4b'}, \quad b'<\frac{1}{2}. \end{aligned}$$

This completes the proof of inequality (4.34). $\square$

**Bilinear estimate for** $$s< 0$$ If $$|\xi _1|\le 10$$ or $$|\xi -\xi _1|\le 10$$, then $$ \frac{ (1+|\xi |)^s }{ (1+|\xi -\xi _1|)^s (1+|\xi _1|)^s } \lesssim 1$$ for $$ s<0, $$ which gives us that $$Q'\lesssim Q_0$$. Thus, for $$s<0$$, in order to prove $$L^2$$ bilinear estimate (4.3), it suffices to show

4.39 $$\begin{aligned} \Big \Vert \int _{\mathbb {R}^2} (\chi _{E} Q_1)(\xi _1,\tau _1,\xi ,\tau ) \cdot c_{f}(\xi -\xi _1,\tau -\tau _1) c_{g}(\xi _1,\tau _1) d\xi _1 d\tau _1 \Big \Vert _{L^2_{\xi ,\tau }} \lesssim \Vert c_{f}\Vert _{L^2_{\xi ,\tau }} \Vert c_{g}\Vert _{L^2_{\xi ,\tau }}, \end{aligned}$$

where the region *E* is defined by

4.40 $$\begin{aligned} E \doteq \{ (\xi _1,\tau _1,\xi ,\tau )\in A: |\xi _1|>10 \,\, \text {and} \,\, |\xi -\xi _1|>10 \}, \end{aligned}$$

and the multiplier $Q_1$ is defined as follows

4.41 $$\begin{aligned}  &   Q_1(\xi _1,\tau _1,\xi ,\tau ) \doteq \frac{ (1+|\xi |)^s }{ |\xi _1(\xi -\xi _1)|^{s} } \cdot \frac{1}{(1+|\tau +\xi ^m|)^{b'}} \nonumber \\  &   \quad \cdot \frac{1}{ (1+|\tau _1+\xi _{1}^{m}|)^{b'} (1+|\tau -\tau _1+(\xi -\xi _1)^m|)^{b'} }. \end{aligned}$$

Furthermore, we will consider the following two possible microlocalizations:

$\bullet$ **Microlocalization I.** It is defined by the region

4.42 $$\begin{aligned} E_{1} \doteq \big \{ (\xi _1,\tau _1,\xi ,\tau ) \in E: |\tau -\tau _1+(\xi -\xi _1)^m| \le |\tau _1+\xi _1^m| \le |\tau +\xi ^m| \big \}. \end{aligned}$$

$\bullet$ **Microlocalization II**. It is defined by the region

4.43 $$\begin{aligned} E_{2} \doteq \big \{ (\xi _1,\tau _1,\xi ,\tau ) \in E: |\tau +\xi ^{m}|< |\tau _1+\xi _1^m| \quad \text {and} \quad |\tau -\tau _1+(\xi -\xi _1)^m| \le |\tau _1+\xi _1^m| \big \}. \end{aligned}$$

**Proof in Microlocalization I.** Here, we have $|\tau +\xi ^m|=\max \big \{ |\tau +\xi ^m|, |\tau _1+\xi _1^m|, \big | \tau -\tau _1+(\xi -\xi _1)^m \big | \big \}$. In this situation, the $L^2$ bilinear estimate (4.39) reads

4.44 $$\begin{aligned}&\Big \Vert \int _{\mathbb {R}^2} \chi _{E_{1}} (\xi _1,\tau _1,\xi ,\tau ) \cdot Q_1 (\xi _1,\tau _1,\xi ,\tau ) \cdot c_{f}(\xi -\xi _1,\tau -\tau _1) c_{g}(\xi _1,\tau _1) d\xi _1 d\tau _1 \Big \Vert _{L^2_{\xi ,\tau }} \nonumber \\&\quad \lesssim \Vert c_{f}\Vert _{L^2_{\xi ,\tau }} \Vert c_{g}\Vert _{L^2_{\xi ,\tau }}. \end{aligned}$$

Working similarly to inequality (4.23), using the Cauchy–Schwarz inequality with respect to $\xi _1,\tau _1$, we see that the left-hand side of $L^2$ bilinear estimate (4.44) is bounded by

4.45 $$\begin{aligned} \Big \Vert \int _{\mathbb {R}^2} \chi _{E_{1}}(\xi _1,\tau _1,\xi ,\tau ) \cdot Q_1^2(\xi _1,\tau _1,\xi ,\tau ) d\xi _1 d\tau _1 \Big \Vert _{L^{\infty }_{\xi ,\tau } }^{1/2} \cdot \Vert c_{f}\Vert _{L^2_{\xi ,\tau }} \Vert c_{g}\Vert _{L^2_{\xi ,\tau }}. \end{aligned}$$

Thus, to prove $L^2$ bilinear estimate (4.44), it suffices to show the following result.

#### Lemma 4.4

Let $m=2j\ge 2$ be an even number. If $-j+\frac{1}{4}<s<0$ and $\max \{\frac{1}{3}-\frac{s}{3\,m},\frac{1}{6}-\frac{4\,s-1}{6\,m}\}\le b'<\frac{1}{2}$, then for $\xi ,\tau \in \mathbb {R}$, we have

4.46 $$\begin{aligned}&\Theta _{3} \doteq \frac{1}{ (1+|\tau +\xi ^{m}|)^{2b'} } \int _{\mathbb {R}^2} \nonumber \\&\quad \frac{ (1+|\xi |)^{2s} }{ |\xi _1(\xi -\xi _1)|^{2s} } \frac{ \chi _{E_{1}} (\xi _1,\tau _1,\xi ,\tau ) d\tau _1 d\xi _1 }{ (1+|\tau -\tau _1+(\xi -\xi _1)^m|)^{2b'} (1+|\tau _1+\xi _1^m|)^{2b'} } \, \lesssim 1. \end{aligned}$$

#### Proof of Lemma 4.4

For the $\tau _1$ integral in (4.46), applying calculus estimate (3.24) with $\ell =\ell '=b'>\frac{1}{4}$, $x=\tau _1$, $\alpha =\tau +(\xi -\xi _1)^m$, $\beta =-\xi _1^m$, we get

4.47 $$\begin{aligned}&\Theta _{3} \lesssim \frac{1}{ (1+|\tau +\xi ^{m}|)^{2b'} } \int _{\mathbb {R}} \frac{ (1+|\xi |)^{2s} }{ |\xi _1(\xi -\xi _1)|^{2s} } \frac{ \chi _{E_{1}} (\xi _1,\tau _1,\xi ,\tau ) \, d\xi _1 }{ (1+|\tau +(\xi -\xi _1)^m+\xi _1^m|)^{4b'-1} } \nonumber \\&\quad = \frac{1}{ (1+|\tau +\xi ^{m}|)^{2b'} } I_3, \end{aligned}$$

where the integral $I_3$ is defined by

4.48 $$\begin{aligned} I_3(\xi ,\tau ) \doteq \int _{\mathbb {R}} \frac{ (1+|\xi |)^{2s} }{ |\xi _1(\xi -\xi _1)|^{2s} } \frac{ \chi _{E_{1}} (\xi _1,\tau _1,\xi ,\tau ) }{ (1+|\tau +\xi ^m-d_{1,m}(\xi ,\xi _1)|)^{4b'-1} } d\xi _1, \end{aligned}$$

where $d_{1,m}(\xi ,\xi _1)=\xi ^m-\xi _1^m-(\xi -\xi _1)^m$. Furthermore, similar to the proof of inequality (3.56), we can prove the following estimate for the integral $I_3$

4.49 $$\begin{aligned}&I_3(\xi ,\tau ) \lesssim \frac{1}{ (1+|\tau +\xi ^{m}|)^{4b'+2s/m-2} } + \frac{1}{ (1+|\tau +\xi ^{m}|)^{4b'+(4s+m-1)/m-2} } , \nonumber \\&\quad \quad s<0 \,\, \text {and} \,\, \frac{1}{4}\le b'<\frac{1}{2}. \end{aligned}$$

Next, combining inequality (4.49) with (4.47), we obtain

4.50 $$\begin{aligned} \Theta _{3} \lesssim \frac{1}{ (1+|\tau +\xi ^{m}|)^{6b'+2s/m-2} } + \frac{1}{ (1+|\tau +\xi ^{m}|)^{6b'+(4s+m-1)/m-2} }, \end{aligned}$$

which is same to the inequality (3.57). Thus, we can conclude that $\Theta _3\lesssim 1$ when $-j+\frac{1}{4}<s<0$ and $\max \{\frac{1}{3}-\frac{s}{3\,m},\frac{1}{6}-\frac{4\,s-1}{6\,m}\}\le b'<\frac{1}{2}$. This completes the proof of Lemma 4.4. $\square$

**Proof in microlocalization II** In this situation, we have $|\tau _1+\xi _1^m|=\max \big \{ |\tau +\xi ^m|, |\tau _1+\xi _1^m|, \big | \tau -\tau _1+(\xi -\xi _1)^m \big | \big \}$. The $L^2$ bilinear estimate (4.39) reads

4.51 $$\begin{aligned}&\Big \Vert \int _{\mathbb {R}^2} \chi _{E_2}(\xi _1,\tau _1,\xi ,\tau )\cdot Q_1(\xi _1,\tau _1,\xi ,\tau ) \cdot c_{f}(\xi -\xi _1,\tau -\tau _1) c_{g}(\xi _1,\tau _1) d\xi _1 d\tau _1 \Big \Vert _{L^2_{\xi ,\tau }}\nonumber \\  &\quad \lesssim \Vert c_{f}\Vert _{L^2_{\xi ,\tau }} \Vert c_{g}\Vert _{L^2_{\xi ,\tau }}. \end{aligned}$$

Like inequality (4.30), by utilizing duality and applying the Cauchy–Schwarz inequality twice, we get

4.52 $$\begin{aligned} \text {LHS of }{(4.51)} \lesssim \Big \Vert \int _{\mathbb {R}^2} \chi _{E_2}(\xi _1,\tau _1,\xi ,\tau )\cdot Q_1(\xi _1,\tau _1,\xi ,\tau ) d\xi d\tau \Big \Vert _{L^{\infty }_{\xi _1,\tau _1} }^{1/2} \cdot \Vert c_{f}\Vert _{L^2_{\xi ,\tau }} \Vert c_{g}\Vert _{L^2_{\xi ,\tau }}. \end{aligned}$$

Thus, to prove $L^2$ bilinear estimate (4.51), it suffices to show the following result.

#### Lemma 4.5

Let $m=2j\ge 2$ be an even number. If $-j+\frac{1}{4}<s<0$ and $\max \{\frac{1}{3}-\frac{s}{3\,m},\frac{1}{6}-\frac{4\,s-1}{6\,m}\}\le b'<\frac{1}{2}$, then for $\xi _1,\tau _1\in \mathbb {R}$, we have

4.53 $$\begin{aligned}&\Theta _{4} \doteq \frac{1}{ (1+|\tau _1+\xi _1^{m}|)^{2b'} } \int _{\mathbb {R}^2} \nonumber \\&\quad \frac{ (1+|\xi |)^{2s} }{ |\xi _1(\xi -\xi _1)|^{2s} } \frac{ \chi _{E_{2}} (\xi _1,\tau _1,\xi ,\tau ) \, d\tau d\xi }{ (1+|\tau -\tau _1+(\xi -\xi _1)^m|)^{2b'} (1+|\tau +\xi ^m|)^{2b'} } \lesssim 1. \end{aligned}$$

#### Proof of Lemma 4.5

For the $\tau$ integral in (4.53), applying calculus estimate (3.24) with $\ell =\ell '=b'>\frac{1}{4}$, $x=\tau _1$, $\alpha =\tau _1-(\xi -\xi _1)^m$, $\beta =-\xi ^m$, we get

4.54 $$\begin{aligned}&\Theta _{4} \lesssim \frac{1}{ (1+|\tau _1+\xi _1^{m}|)^{2b'} } \int _{\mathbb {R}} \frac{ (1+|\xi |)^{2s} }{ |\xi _1(\xi -\xi _1)|^{2s} } \frac{ \chi _{E_{2}} (\xi _1,\tau _1,\xi ,\tau ) \,d\xi }{ (1+|\tau _1-(\xi -\xi _1)^m+\xi ^m|)^{4b'-1} } \nonumber \\&\quad = \frac{1}{ (1+|\tau _1+\xi _1^{m}|)^{2b'} } I_4, \end{aligned}$$

where the integral $I_4$ is defined by

4.55 $$\begin{aligned} I_4(\xi _1,\tau _1) \doteq \int _{\mathbb {R}} \frac{ (1+|\xi |)^{2s} }{ |\xi _1(\xi -\xi _1)|^{2s} } \frac{ \chi _{E_{2}} (\xi _1,\tau _1,\xi ,\tau ) }{ (1+|\tau _1+\xi _1^m+d_{1,m}(\xi ,\xi _1)|)^{4b'-1} } d\xi , \end{aligned}$$

with $d_{1,m}(\xi ,\xi _1)=\xi ^m-\xi _1^m-(\xi -\xi _1)^m$. Furthermore, later we will prove the following bound for $I_4$

4.56 $$\begin{aligned}&I_4 \lesssim \frac{1}{ (1+|\tau _1-\xi ^{m}|)^{4b'+2s/m-2} } + \frac{1}{ (1+|\tau _1-\xi _1^{m}|)^{4b'+(4s+m-1)/m-2} } , \nonumber \\&\quad \quad s<0 \,\, \text {and} \,\, \frac{1}{4}\le b'<\frac{1}{2}. \end{aligned}$$

Next, combining inequality (4.56) with (4.54), we obtain

4.57 $$\begin{aligned} \Theta _{4} \lesssim \frac{1}{ (1+|\tau _1-\xi _1^{m}|)^{6b'+2s/m-2} } + \frac{1}{ (1+|\tau _1-\xi _1^{m}|)^{6b'+(4s+m-1)/m-2} }, \end{aligned}$$

which is similar to inequality (3.57). Therefore, we have $\Theta _4\lesssim 1$ for $-j+\frac{1}{4}<s<0$ and $\max \{\frac{1}{3}-\frac{s}{3\,m},\frac{1}{6}-\frac{4\,s-1}{6\,m}\}\le b'<\frac{1}{2}$. This completes the proof of Lemma 4.5 once we prove inequality (4.56). $\square$

**Proof of inequality **(4.56) Using comparison property (4.7) and inequality (4.8), we get

4.58 $$\begin{aligned}  &   |\tau _1+\xi _1^m| \gtrsim |d_{1,m}(\xi ,\xi _1)| \gtrsim \max \big \{ |\xi -\xi _1||\xi _1| (\xi ^{m-2}+\xi _1^{m-2}), |\xi _1(\xi -\xi _1)|^{\frac{1}{2}m} \big \} > 1, \nonumber \\  &   \quad (\xi _1,\tau _1,\xi ,\tau )\in E_2. \end{aligned}$$

Also, without loss of generality we assume that $\xi _1>0$. Furthermore, we will consider three cases: $|\xi |\lesssim \xi _1$ and $|\xi |\gtrsim \xi _1$. More precisely, we rewrite $I_4=I_{4,1}+I_{4,2}+I_{4,3}$, where

4.59 $$\begin{aligned} I_{4,1}(\xi _1,\tau _1) \doteq&\int _{ -\frac{1}{10}\xi _1}^{ \frac{1}{10}\xi _1} \frac{ (1+|\xi |)^{2s} }{ |\xi _1(\xi -\xi _1)|^{2s} } \frac{ \chi _{E_{2}} (\xi _1,\tau _1,\xi ,\tau ) }{ (1+|\tau _1+\xi _1^m+d_{3,m}(\xi ,\xi _1)|)^{4b'-1} } d\xi , \end{aligned}$$

4.60 $$\begin{aligned} I_{4,2}(\xi _1,\tau _1) \doteq&\int _{\frac{1}{10}\xi _1}^\infty \frac{ (1+|\xi |)^{2s} }{ |\xi _1(\xi -\xi _1)|^{2s} } \frac{ \chi _{E_{2}} (\xi _1,\tau _1,\xi ,\tau ) }{ (1+|\tau _1+\xi _1^m+d_{3,m}(\xi ,\xi _1)|)^{4b'-1} } d\xi , \end{aligned}$$

4.61 $$\begin{aligned} I_{4,3}(\xi _1,\tau _1) \doteq&\int _{-\infty }^{-\frac{1}{10}\xi _1} \frac{ (1+|\xi |)^{2s} }{ |\xi _1(\xi -\xi _1)|^{2s} } \frac{ \chi _{E_{2}} (\xi _1,\tau _1,\xi ,\tau ) }{ (1+|\tau _1+\xi _1^m+d_{3,m}(\xi ,\xi _1)|)^{4b'-1} } d\xi . \end{aligned}$$

Since the estimation for $I_{4,3}$ is similar to that of $I_{4,2}$, here we only estimate $I_{4,1}$ and $I_{4,2}$.

$\underline{\hbox {Estimate for }I_{4,1}.}$ Making the change of variables $\mu =\mu (\xi )=\tau _1+\xi _1^m+d_{1,m}(\xi ,\xi _1)$ or $\xi =\xi (\mu )$, we get

4.62 $$\begin{aligned} I_{4,1}(\xi _1, \tau _1) \lesssim \int _{ \mu (-\frac{1}{10}\xi _1)} ^{ \mu (\frac{1}{10}\xi _1)} \frac{ (1+|\xi (\mu )|)^{2s} }{ |\xi _1(\xi (\mu )-\xi _1)|^{2s} } \frac{ \chi _{E_{2}} (\xi _1,\tau _1,\xi ,\tau ) }{ (1+|\mu |)^{4b'-1} } \frac{1}{|\partial _{\xi }\mu |} d\mu . \end{aligned}$$

Since $\partial _{\xi }\mu =\partial _{\xi }d_{1,m}(\xi ,\xi _1)$, using the lower bound for $\partial _{\xi }d_{1,m}$, i.e., inequality (4.10), we get $|\partial _{\xi _1}\mu |\gtrsim |\xi _1|^{m-1}$.

Also, if $|\xi |< \frac{1}{10}|\xi _1|$, then we have $|\xi -\xi _1| \le |\xi _1|+|\xi | \le |\xi _1|+\frac{1}{10}|\xi _1| \le \frac{11}{10}|\xi _1|,$ and $|\xi -\xi _1| \ge |\xi _1|-|\xi | \ge |\xi _1|-\frac{1}{10}|\xi _1| = \frac{9}{10}|\xi _1|,$ which gives us

4.63 $$\begin{aligned} |\xi -\xi _1|\simeq |\xi _1|, \quad \text {if} \,\, |\xi |\le \frac{1}{10}\xi _1. \end{aligned}$$

This implies that $\chi _{|\xi |< \frac{1}{10}|\xi _1|}(\xi ) \cdot \frac{ (1+|\xi |)^{2\,s} }{ |\xi _1(\xi -\xi _1)|^{2\,s} } \lesssim \frac{ 1 }{ |\xi _1|^{4\,s} }$, for $s<0.$ Combining the above inequalities, we get

4.64 $$\begin{aligned} I_{4,1}(\xi _1,\tau _1) \lesssim&\int _{ \mu (-\frac{1}{10}\xi _1)} ^{ \mu (\frac{1}{10}\xi _1)} \frac{ 1 }{ |\xi _1|^{4s+m-1} } \frac{ \chi _{E_{2}} (\xi _1,\tau _1,\xi ,\tau ) }{ (1+|\mu |)^{4b'-1} } d\mu . \end{aligned}$$

Since $|\xi _1|>1$, we notice that if *s* is smaller, then the integral $I_{4,1}$ becomes bigger. Thus, we assume that $4\,s+m-1\le 0$. Furthermore, using inequality (4.58) and inequality (4.63), we obtain $1 < |\xi _1| \le |\tau _1+\xi _1^m|^{1/m}$, when $|\xi |< \frac{1}{10}|\xi _1|$ and $(\xi _1,\tau _1,\xi ,\tau )\in E_2.$ Next, using these inequalities and integrating over whole $\mathbb {R}$, from (4.64) we get

4.65 $$\begin{aligned} I_{4,1}(\xi _1,\tau _1) \lesssim \frac{1}{ (1+|\tau _1+\xi _1^m|)^{(4s+m-1)/m} } \int _{\mathbb {R}} \frac{ \chi _{E_{2}} (\xi _1,\tau _1,\xi ,\tau ) }{ (1+|\mu |)^{4b'-1} } d\mu . \end{aligned}$$

Finally, using triangle inequality and using inequality (4.58), we get $|\mu |\le |\tau _1+\xi _1^m|+|d_{1,m}|\lesssim |\tau _1+\xi _1^m|$ for $(\xi _1,\tau _1,\xi ,\tau ) \in E_2$. This gives us that

4.66 $$\begin{aligned}  &   \int _{\mathbb {R}} \frac{ \chi _{E_{2}} (\xi _1,\tau _1,\xi ,\tau ) }{ (1+|\mu |)^{4b'-1} } d\mu \lesssim \int _{|\mu |\lesssim |\tau _1+\xi _1^m|} \frac{ 1 }{ (1+|\mu |)^{4b'-1} } d\mu \lesssim (1+|\tau _1+\xi _1^m|)^{2-4b'}, \nonumber \\  &   \quad \quad b'<\frac{1}{2}. \end{aligned}$$

Therefore, from inequality (4.65), for $4b'-1<1$ or $b'<\frac{1}{2}$ we get the desired inequality (4.56) for $I_{4,1}$.

$\underline{\hbox {Estimate for }I_{4,2}.}$ Making the change of variables $\mu =\mu (\xi )=\tau _1+\xi _1^m+d_{1,m}(\xi ,\xi _1)$ or $\xi =\xi (\mu )$, we get

4.67 $$\begin{aligned} I_{4,2}(\xi _1, \tau _1) \lesssim \int _{\mu ( \frac{1}{10}\xi _1) }^\infty \frac{ (1+|\xi |)^{2s} }{ |\xi _1(\xi -\xi _1)|^{2s} } \frac{ \chi _{E_{2}} (\xi _1,\tau _1,\xi ,\tau ) }{ (1+|\mu |)^{4b'-1} } \frac{1}{|\partial _{\xi }\mu |} d\mu . \end{aligned}$$

Since $\partial _{\xi }\mu =\partial _{\xi }d_{1,m}(\xi ,\xi _1)$, using the lower bound for $\partial _{\xi }d_{1,m}$, i.e., inequality (4.10), we get $|\partial _{\xi }\mu |\gtrsim |\xi _1|^{m-1}\gtrsim 1$ for $|\xi _1|>1$. Furthermore, if $\xi \ge \frac{1}{10}\xi _1$ (with $\xi _1>0$), then we have $|\xi _1|\lesssim |\xi |$, which gives us $|\xi -\xi _1|\lesssim |\xi |+|\xi _1|\lesssim |\xi |$. This implies that $\chi _{\xi \ge \frac{1}{10}\xi _1}(\xi ) \cdot \frac{ (1+|\xi |)^{2\,s} }{ |\xi _1(\xi -\xi _1)|^{2\,s} } \lesssim \frac{ 1 }{ |\xi _1(\xi -\xi _1)|^{s} },$ for $s<0.$ Furthermore, using inequality (4.58), we obtain $1 < |\xi _1(\xi -\xi _1)| \lesssim |\tau _1+\xi _1^m|^{2/m},$ when $(\xi _1,\tau _1,\xi ,\tau )\in E_2.$ Combining these inequalities and integrating over whole $\mathbb {R}$, we get

4.68 $$\begin{aligned} I_{4,2}(\xi _1,\tau _1) \lesssim&\int _{\mathbb {R}} \frac{ 1 }{ |\tau _1+\xi _1^m|^{2s/m} } \frac{ \chi _{E_{2}} (\xi _1,\tau _1,\xi ,\tau ) }{ (1+|\mu |)^{4b'-1} } d\mu \nonumber \\ \lesssim&\frac{ 1 }{ (1+|\tau _1+\xi _1^m|)^{2s/m} } \int _{\mathbb {R}} \frac{ \chi _{E_{2}} (\xi _1,\tau _1,\xi ,\tau ) }{ (1+|\mu |)^{4b'-1} } d\mu . \end{aligned}$$

Combining inequality (4.66) with (4.68), we get the desired inequality (4.56) for $I_{4,2}$. This completes the proof of inequality (4.56).    $\square$

### Optimality of *fg* spatial bilinear estimates

Working in a way similar to Lemma 3.6, we can establish the following result

#### Lemma 4.6

If $s\le -j+\frac{1}{4}$, then bilinear estimate $\Vert fg\Vert _{X^{s,-b}} \lesssim \Vert f\Vert _{X^{s,b}} \Vert g\Vert _{X^{s,b}}$ fails for any $b<\frac{1}{2}$.

Hence, Lemma 4.6 demonstrates the optimality of *fg* spatial bilinear estimates.

## $f\bar{g}$ bilinear estimate in spatial Bourgain spaces

Here, we prove the spatial bilinear estimate for the asymmetric nonlinearity $f\bar{g}$ and show its optimality.

### $f\bar{g}$ spatial bilinear estimate

Like in the case of $\bar{f}\bar{g}$ spatial bilinear estimate (inequality (3.3)), we reduce the proof of $f\bar{g}$ spatial bilinear estimate to the proof of the following $L^2$ estimate

5.1 $$\begin{aligned} \Big \Vert \int _{\mathbb {R}^2} Q(\xi _1,\tau _1,\xi ,\tau ) \cdot c_{f}(\xi -\xi _1,\tau -\tau _1) c_{g}(\xi _1,\tau _1) d\xi _1 d\tau _1 \Big \Vert _{L^2_{\xi ,\tau }} \lesssim \Vert c_{f}\Vert _{L^2_{\xi ,\tau }} \Vert c_{g}\Vert _{L^2_{\xi ,\tau }}. \end{aligned}$$

where the multiplier $Q=Q(\xi _1,\tau _1,\xi ,\tau )$ is defined as

5.2 $$\begin{aligned} Q&\doteq \frac{ (1+|\xi |)^s }{ (1+|\xi _1|)^{s} (1+|\xi -\xi _1|)^{s} } \cdot \frac{1}{(1+|\tau +\xi ^m|)^{b}} \nonumber \\&\quad \cdot \frac{1}{ (1+|\tau _1-\xi _{1}^{m}|)^{b'} (1+|\tau -\tau _1+(\xi -\xi _1)^m|)^{b'} }. \end{aligned}$$

Furthermore, using $b'\le b$ in order to show $L^2$ bilinear estimate (5.1), it suffices to show that

5.3 $$\begin{aligned} \Big \Vert \int _{\mathbb {R}^2} Q' (\xi _1,\tau _1,\xi ,\tau ) \cdot c_{f}(\xi -\xi _1,\tau -\tau _1) c_{g}(\xi _1,\tau _1) d\xi _1 d\tau _1 \Big \Vert _{L^2_{\xi ,\tau }} \lesssim \Vert c_{f}\Vert _{L^2_{\xi ,\tau }} \Vert c_{g}\Vert _{L^2_{\xi ,\tau }}, \end{aligned}$$

where the multiplier $Q'$ is defined as

5.4 $$\begin{aligned}&Q' \doteq \frac{ (1+|\xi |)^s }{ (1+|\xi _1|)^{s} (1+|\xi -\xi _1|)^{s} } \cdot \frac{1}{(1+|\tau +\xi ^m|)^{b'}}\nonumber \\&\quad \cdot \frac{1}{ (1+|\tau _1-\xi _{1}^{m}|)^{b'} (1+|\tau -\tau _1+(\xi -\xi _1)^m|)^{b'} }. \end{aligned}$$

From the multiplier $Q'$, we recognize the familiar Bourgain quantity for nonlinearity $f\bar{g}$

5.5 $$\begin{aligned} d_{2,m}(\xi ,\xi _1) \doteq \xi ^m+\xi _1^m-(\xi -\xi _1)^m = (\tau +\xi ^m)-(\tau _1-\xi _1^m)-[(\tau -\tau _1)+(\xi -\xi _1)^m].\nonumber \\ \end{aligned}$$

Here are some useful elementary properties of $d_{2,m}(\xi ,\xi _1)$, whose proof is similar to the one for $d_{3,m}$.

#### Proposition 5.1

If $m=2j\ge 2$ is an even number, then $d_{2,m}(\xi ,\xi _1)$ is a symmetric homogeneous polynomial of $\xi$ and $\xi _1$ of degree *m*, and has the following properties:

5.6 $$\begin{aligned}  &   \bullet \hbox { Upper bound for} d_{2,m}(\xi ,\xi _1): \qquad |d_{2,m}(\xi ,\xi _1)| \lesssim \max \{ \xi ^m, \xi _1^m \}. \end{aligned}$$

5.7 $$\begin{aligned}  &   \bullet \,\, \partial _\xi d_{2,m}(\xi ,\xi _1) = m\xi ^{m-1}-m(\xi -\xi _1)^{m-1} \quad \nonumber \\  &   \qquad \text {and} \quad \partial _{\xi _1} d_{2,m}(\xi ,\xi _1) = m\xi _1^{m-1}-m(\xi _1-\xi )^{m-1}. \end{aligned}$$

5.8 $$\begin{aligned}  &   \bullet \hbox { Lower bound for partial derivatives:} \quad |\partial _\xi d_{2,m}(\xi ,\xi _1)| \gtrsim |\xi _1|^{m-1} \,\, \nonumber \\  &   \qquad \text {and} \,\, |\partial _{\xi _1} d_{2,m}(\xi ,\xi _1)| \gtrsim |\xi |^{m-1}. \end{aligned}$$

$\bullet$ Monotonicity: When considering $d_{2,m}(\xi ,\xi _1)$ as a function of $\xi$, it exhibits an increasing monotonicity when $\xi _1>0$ and a decreasing monotonicity when $\xi _1<0$. When considering $d_{2,m}(\xi ,\xi _1)$ as a function of $\xi _1$, it exhibits an increasing monotonicity when $\xi >0$ and a decreasing monotonicity when $\xi <0$.

$\bullet$ Zeros of $d_{2,m}(\xi ,\xi _1)$: When considering $d_{2,m}(\xi ,\xi _1)$ as a function of $\xi$, it has zeros at $\xi =0$. When considering $d_{2,m}(\xi ,\xi _1)$ as a function of $\xi _1$, it has zeros at $\xi _1=0$.

**Fig. 10:**
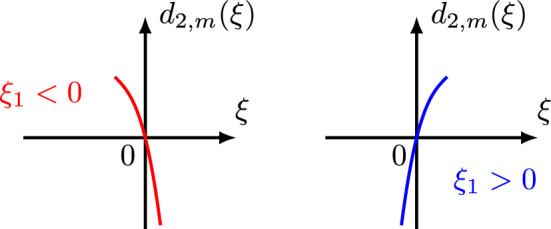
$d_{2,m}$ is a function of $\xi$

**Fig. 11:**
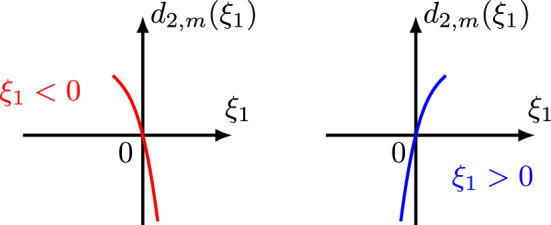
$d_{2,m}$ is a function of $\xi _1$

$\bullet$ Graphs: Figs. 10 and 11 display the graph of $d_{2,m}(\xi ,\xi _1)$ as a function of $\xi$ and $\xi _1$, respectively.

Next, we provide the proof of $L^2$ bilinear estimate (5.3) for the following two cases:

$$\bullet $$ Case: $$s\ge 0$$       $$\bullet $$ Case: $$s< 0$$.

**Bilinear estimate for** $s\ge 0$ Then, $(1+|\xi |)^s \le (1+|\xi -\xi _1|)^s (1+|\xi _1|)^s$, which gives $Q'\le Q_0$, where

5.9 $$\begin{aligned} Q_0 \doteq \frac{1}{(1+|\tau +\xi ^m|)^{b'}} \frac{1}{ (1+|\tau _1-\xi _{1}^{m}|)^{b'} (1+|\tau -\tau _1+(\xi -\xi _1)^m|)^{b'} }. \end{aligned}$$

Therefore, when $s\ge 0$ in order to prove $L^2$ inequality (5.1), it suffices to show that

5.10 $$\begin{aligned} \Big \Vert \int _{\mathbb {R}^2} Q_0(\xi _1,\tau _1,\xi ,\tau ) \cdot c_{f}(\xi -\xi _1,\tau -\tau _1) c_{g}(\xi _1,\tau _1) d\xi _1 d\tau _1 \Big \Vert _{L^2_{\xi ,\tau }} \lesssim \Vert c_{f}\Vert _{L^2_{\xi ,\tau }} \Vert c_{g}\Vert _{L^2_{\xi ,\tau }}. \end{aligned}$$

We do this by considering the following three microlocalizations:

$$\bullet $$ **Microlocalization I:** $$(\xi _1,\tau _1,\xi ,\tau )\in A_1$$, with $$ A_1 \doteq \{ (\xi _1,\tau _1,\xi ,\tau )\in \mathbb {R}^4: |\xi _1|\le 1 \}. $$

$\bullet$ **Microlocalization II:** $(\xi _1,\tau _1,\xi ,\tau )\in A_2$, with $A_2 \doteq \{ (\xi _1,\tau _1,\xi ,\tau )\in \mathbb {R}^4: |\xi |\le 1 \}.$

$\bullet$ **Microlocalization III:** $(\xi _1,\tau _1,\xi ,\tau )\in A_3$, with $A_3 \doteq \{ (\xi _1,\tau _1,\xi ,\tau )\in \mathbb {R}^4: |\xi _1|> 1 \,\, \text {and} \,\, |\xi |>1 \}.$

**Proof in Microlocalization I** ($|\xi _1|\le 1$). Working similarly to inequality (3.22), we get

$$\begin{aligned}&\Big \Vert \int _{\mathbb {R}^2} (\chi _{A_1} Q_0) \cdot c_{f}(\xi -\xi _1,\tau -\tau _1) c_{g}(\xi _1,\tau _1) d\xi _1 d\tau _1 \Big \Vert _{L^2_{\xi ,\tau }}\\&\quad \lesssim \Vert c_{f}\Vert _{L^2_{\xi ,\tau }} \Vert c_{g}\Vert _{L^2_{\xi ,\tau }} \Big \Vert \int _{\mathbb {R}^2} (\chi _{A_1} Q_0^2) d\xi _1 d\tau _1 \Big \Vert _{L^{\infty }_{\xi ,\tau } }^{1/2}. \end{aligned}$$

Thus, to prove $L^2$ bilinear estimate (5.10) in this situation, it suffices to show the following result.

#### Lemma 5.1

Let $m=2j\ge 2$ be an even number. If $\frac{1}{4}<b'<\frac{1}{2}$, then for $\xi ,\tau \in \mathbb {R}$, we have

5.11 $$\begin{aligned}&\Theta _1 (\xi , \tau ) \doteq \frac{1}{ (1+|\tau +\xi ^{m}|)^{2b'} } \int _{\mathbb {R}^2} \nonumber \\&\qquad \frac{ \chi _{A_1}(\xi _1,\tau _1,\xi ,\tau ) }{ (1+|\tau -\tau _1+(\xi -\xi _1)^m|)^{2b'} (1+|\tau _1-\xi _1^m|)^{2b'} } \, d\tau _1 d\xi _1 \lesssim 1. \end{aligned}$$

#### Proof of Lemma 5.1

The integral of $\xi _1$ is over the finite interval $[-1,1]$, making the proof of this lemma analogous to the proof of Lemma 4.1. Hence, we omit the detailed proof of this result. $\square$

**Proof in Microlocalization II** ($|\xi |\le 1$). Working similarly to $L^2$ bilinear estimate (3.39), using duality and Cauchy–Schwarz inequality twice we get

$$\begin{aligned}&\Big \Vert \int _{\mathbb {R}^2} (\chi _{A_2} Q_0) \cdot c_{f}(\xi -\xi _1,\tau -\tau _1) c_{g}(\xi _1,\tau _1) d\xi _1 d\tau _1 \Big \Vert _{L^2_{\xi ,\tau }} \\&\qquad \lesssim \Vert c_{f}\Vert _{L^2_{\xi ,\tau }} \Vert c_{g}\Vert _{L^2_{\xi ,\tau }} \Big \Vert \int _{\mathbb {R}^2} (\chi _{A_1} Q_0^2) d\xi _1 d\tau _1 \Big \Vert _{L^{\infty }_{\xi ,\tau } }^{1/2}. \end{aligned}$$

Thus, to prove $L^2$ bilinear estimate (5.10) in this situation, it suffices to show the following result.

#### Lemma 5.2

Let $m=2j\ge 2$ be an even number. If $\frac{1}{4}<b'<\frac{1}{2}$, then for $\xi _1,\tau _1\in \mathbb {R}$, we have

5.12 $$\begin{aligned}&\Theta _2 (\xi _1, \tau _1) \doteq \frac{1}{ (1+|\tau _1-\xi _{1}^{2}|)^{2b'} } \int _{\mathbb {R}^2}\nonumber \\&\qquad \frac{ \chi _{A_2} (\xi _1,\tau _1,\xi ,\tau ) }{ (1+|\tau -\tau _1+(\xi -\xi _1)^2|)^{2b'} (1+|\tau +\xi ^2|)^{2b'} } \, d\tau d\xi \lesssim 1. \end{aligned}$$

#### Proof of Lemma 5.2

Since the integral of $\xi$ is taken over the finite interval $[-1,1]$, the proof of this lemma follows a similar approach as that of Lemma 4.1. Hence, we omit the proof of this result. $\square$

**Proof in Microlocalization III** ($|\xi _1|>1$ and $|\xi |>1$). Here, by comparing the size of $|\xi |$ and the size of $|\xi _1|$, we consider the following two possible microlocalizations:

$\bullet$ **Microlocalization III-1.** ($|\xi _1|\le |\xi |$). It is defined by the region

$$\begin{aligned} A_{3,1} \doteq \big \{ (\xi _1,\tau _1,\xi ,\tau ) \in \mathbb {R}^4: |\xi _1|>1, \,\, |\xi |>1 \,\, \text {and} \,\, |\xi _1|\le |\xi | \big \}. \end{aligned}$$

$\bullet$ **Microlocalization III-2. **($|\xi _1|> |\xi |$). It is defined by the region

$$\begin{aligned} A_{3,2} \doteq \big \{ (\xi _1,\tau _1,\xi ,\tau ) \in \mathbb {R}^4: |\xi _1|>1, \,\, |\xi |>1 \,\, \text {and} \,\, |\xi _1|> |\xi | \big \}. \end{aligned}$$

**Proof in Microlocalization III-1.** ($1<|\xi _1|\le |\xi |$). The $L^2$ bilinear estimate (5.10) reads

5.13 $$\begin{aligned} \Big \Vert \int _{\mathbb {R}^2} (\chi _{A_{3,1}}Q_0)(\xi _1,\tau _1,\xi ,\tau ) \cdot c_f(\xi -\xi _1,\tau -\tau _1) c_{g}(\xi _1,\tau _1) d\xi _1 d\tau _1 \Big \Vert _{L^2_{\xi ,\tau }} \lesssim \Vert c_f\Vert _{L^2_{\xi ,\tau }} \Vert c_{g}\Vert _{L^2_{\xi ,\tau }}. \end{aligned}$$

Similar to the proof in microlocalization I, using the Cauchy–Schwarz inequality with respect to $\xi _1,\tau _1$, we see that the left-hand side of above estimate is bounded by

5.14 $$\begin{aligned} \Big \Vert \int _{\mathbb {R}^2} (\chi _{A_{3,1}} Q_0^2)(\xi _1,\tau _1,\xi ,\tau ) d\xi _1 d\tau _1 \Big \Vert _{L^{\infty }_{\xi ,\tau } }^{1/2} \cdot \Vert c_{f}\Vert _{L^2_{\xi ,\tau }} \Vert c_{g}\Vert _{L^2_{\xi ,\tau }}. \end{aligned}$$

In this situation, to prove $L^2$ bilinear estimate (5.10), it suffices to show the following result.

#### Lemma 5.3

Let $m=2j\ge 2$ be an even number. If $\frac{3}{8}\le b'<\frac{1}{2}$, then for $\xi ,\tau \in \mathbb {R}$, we have

5.15 $$\begin{aligned}&\Theta _{3} (\xi , \tau ) \doteq \frac{1}{ (1+|\tau +\xi ^m|)^{2b'} } \int _{\mathbb {R}^2} \nonumber \\&\qquad \frac{ \chi _{A_{3,1}} (\xi _1,\tau _1,\xi ,\tau ) }{ (1+|\tau -\tau _1+(\xi -\xi _1)^m|)^{2b'} (1+|\tau _1-\xi _1^m|)^{2b'} } \, d\tau _1 d\xi _1 \lesssim 1. \end{aligned}$$

#### Proof of Lemma 5.3

For the $\tau _1$ integral in (5.15), applying calculus estimate (3.24) with $\ell =\ell '=b'>\frac{1}{4}$, $x=\tau _1$, $\alpha =\tau +(\xi -\xi _1)^m$, $\beta =\xi _1^m$, we get

5.16 $$\begin{aligned} \Theta _{3}(\xi , \tau )&\lesssim \frac{1}{ (1+|\tau +\xi ^m|)^{2b'} } \int _{\mathbb {R}} \frac{ \chi _{A_{3,1}} (\xi _1,\tau _1,\xi ,\tau ) }{ (1+|\tau +(\xi -\xi _1)^m-\xi _1^m|)^{4b'-1} } d\xi _1 \nonumber \\&= \frac{\chi _{|\xi |>1}(\xi )}{ (1+|\tau +\xi ^m|)^{2b'} } I_3(\xi ,\tau ), \end{aligned}$$

where the integral $I_3$ is defined by

5.17 $$\begin{aligned} I_3(\xi ,\tau )  &   \doteq \int _{|\xi _1|\le |\xi |} \frac{ d\xi _1 }{ (1+|\tau +(\xi -\xi _1)^m-\xi _1^m|)^{4b'-1} }\nonumber \\  &   = \int _{|\xi _1|\le |\xi |} \frac{ d\xi _1 }{ (1+|\tau +\xi ^m-d_{2,m}(\xi ,\xi _1)|)^{4b'-1} }. \end{aligned}$$

Here, $d_{2,m}(\xi ,\xi _1)=\xi ^m+\xi _1^m-(\xi -\xi _1)^m$ is the Bourgain quantity for nonlinearity $f\bar{g}$. Now, we make the change of variables $\mu =\mu (\xi _1)=\tau +\xi ^m-d_{2,m}(\xi ,\xi _1)$, which is good since by Proposition 5.1$\mu (\xi _1)$ is a monotone function. Utilizing inequality (5.6) for $|\xi _1|\le |\xi |$, we obtain $|d_{2,m}|\lesssim \xi ^m$. Thus, we have

$$\begin{aligned} |\mu |  &   = |\tau +\xi ^m-d_{2,m}(\xi ,\xi _1)| \le |\tau +\xi ^m|+|d_{2,m}(\xi ,\xi _1)| \le |\tau +\xi ^m|+\xi ^m \\  &   \lesssim (1+|\tau +\xi ^m|)+\xi ^m. \end{aligned}$$

This change of variables gives us

5.18 $$\begin{aligned} I_{3}  &   \lesssim \int _{|\mu |\lesssim (1+|\tau +\xi ^m|)+\xi ^m} \frac{ 1 }{ (1+|\mu |)^{4b'-1} } \frac{1}{|\partial _{\xi _1}\mu |} d\mu \nonumber \\  &   \lesssim \frac{1}{|\xi |^{m-1}} \int _{|\mu |\lesssim (1+|\tau +\xi ^m|)+\xi ^m} \frac{ 1 }{ (1+|\mu |)^{4b'-1} } d\mu , \end{aligned}$$

where, in the last step, we use $\partial _{\xi _1} \mu =-\partial _{\xi _1}d_{2,m}$ and the inequality (5.8), which states that $|\partial _{\xi _1} d_{2,m}(\xi ,\xi _1)| \gtrsim |\xi |^{m-1}$. Therefore, integrating the variable $\mu$, from inequality (5.18) we get (for $|\xi |>1$)

5.19 $$\begin{aligned} I_{3}(\xi ,\tau )&\lesssim \frac{1}{|\xi |^{m-1}} \big [ (1+|\tau +\xi ^m|)+\xi ^m \big ]^{2-4b'} \nonumber \\&\lesssim \frac{ (1+|\tau +\xi ^m|)^{2-4b'} }{|\xi |^{m-1}} + |\xi |^{m-4mb'+1} , \quad b'<\frac{1}{2}. \end{aligned}$$

In the last step, we apply the inequality $(|\alpha |+|\beta |)^{\mu } \lesssim |\alpha |^\mu +|\beta |^\mu ,$ $\mu \ge 0,$ with $|\alpha |=(1+|\tau +\xi ^m|)$, $|\beta |=\xi ^m$ and $\mu =2-4b'\ge 0$. Combining inequality (5.19) with inequality (5.16) we obtain

$$\begin{aligned} \Theta _3(\xi ,\tau ) \lesssim \chi _{|\xi |>1}(\xi ) \cdot \frac{ (1+|\tau +\xi ^m|)^{2-6b'} }{|\xi |^{m-1}} + \chi _{|\xi |>1}(\xi ) \cdot \frac{ |\xi |^{m-4mb'+1}}{(1+|\tau +\xi ^m|)^{2b'}}, \end{aligned}$$

which is bounded if $2-6b'\le 0$, $m-4mb'+1\le 0$ and $2b'\ge 0$. It suffices to have $b'\ge \frac{1}{3}$, $b'\ge \frac{1}{4}+\frac{1}{4\,m}$ and $b'\ge 0$. For $m\ge 2$, it suffices to have $b'\ge \frac{1}{4}+\frac{1}{8}=\frac{3}{8}$. This completes the proof of Lemma 5.3. $\square$

**Proof in Microlocalization III-2** ($|\xi _1|> |\xi |>1$). Then, the $L^2$ bilinear estimate (5.10) reads

5.20 $$\begin{aligned} \Big \Vert \int _{\mathbb {R}^2} (\chi _{A_{3,2}}Q_0) (\xi _1,\tau _1,\xi ,\tau ) \cdot c_f(\xi -\xi _1,\tau -\tau _1) c_{g}(\xi _1,\tau _1) d\xi _1 d\tau _1 \Big \Vert _{L^2_{\xi ,\tau }} \lesssim \Vert c_f\Vert _{L^2_{\xi ,\tau }} \Vert c_{g}\Vert _{L^2_{\xi ,\tau }}. \end{aligned}$$

Using duality and the Cauchy–Schwarz inequality twice, we have

5.21 $$\begin{aligned} \text {LHS of }{(5.20)} \lesssim \Big \Vert \int _{\mathbb {R}^2} (\chi _{A_{3,2}} Q_0^2)(\xi _1,\tau _1,\xi ,\tau ) d\xi d\tau \Big \Vert _{L^{\infty }_{\xi _1,\tau _1} }^{1/2} \cdot \Vert c_{f}\Vert _{L^2_{\xi ,\tau }} \Vert c_{g}\Vert _{L^2_{\xi ,\tau }}. \end{aligned}$$

Thus, to prove $L^2$ bilinear estimate (5.10) in this situation, it suffices to show the following result.

#### Lemma 5.4

Let $m=2j\ge 2$ be an even number. If $\frac{3}{8}\le b'<\frac{1}{2}$, then for $\xi _1,\tau _1\in \mathbb {R}$, we have

5.22 $$\begin{aligned}&\Theta _{4} (\xi , \tau ) \doteq \frac{1}{ (1+|\tau _1-\xi _1^m|)^{2b'} } \int _{\mathbb {R}^2} \nonumber \\&\qquad \frac{ \chi _{A_{3,2}} (\xi _1,\tau _1,\xi ,\tau ) }{ (1+|\tau -\tau _1+(\xi -\xi _1)^m|)^{2b'} (1+|\tau +\xi ^m|)^{2b'} } \, d\tau d\xi \lesssim 1. \end{aligned}$$

#### Proof of Lemma 5.4

Due to the symmetry of $d_{2,m}(\xi ,\xi _1)$ with respect to $\xi$ and $\xi _1$ (i.e., $d_{2,m}(\xi ,\xi _1)=d_{2,m}(\xi _1,\xi )$), the proof of this result is similar to the proof of Lemma 5.3. $\square$

**Bilinear estimate for** $s< 0$ If $|\xi _1|\le 1$ or $|\xi -\xi _1|\le 1$, then for $s<0$ we have $Q'\lesssim Q_0$. Thus, for $s<0$, in order to prove $L^2$ bilinear estimate (5.3), it suffices to show that

5.23 $$\begin{aligned}&\Big \Vert \int _{\mathbb {R}^2} \chi _{E} (\xi _1,\tau _1,\xi ,\tau ) \cdot Q_1(\xi _1,\tau _1,\xi ,\tau ) \cdot c_f(\xi -\xi _1,\tau -\tau _1) c_{g}(\xi _1,\tau _1) d\xi _1 d\tau _1 \Big \Vert _{L^2_{\xi ,\tau }} \nonumber \\&\quad \lesssim \Vert c_f\Vert _{L^2_{\xi ,\tau }} \Vert c_{g}\Vert _{L^2_{\xi ,\tau }}, \end{aligned}$$

where the region *E* is given by

5.24 $$\begin{aligned} E \doteq \{ (\xi _1,\tau _1,\xi ,\tau )\in \mathbb {R}^4: |\xi _1|>1, \,\, \text{ and } \,\, |\xi -\xi _1|>1 \}, \end{aligned}$$

and $Q_1$ is the multiplier defined by

5.25 $$\begin{aligned} Q_1 \doteq \frac{ (1+|\xi |)^s }{ |\xi _1(\xi -\xi _1)|^{s} } \cdot \frac{1}{(1+|\tau +\xi ^2|)^{b'}} \cdot \frac{1}{ (1+|\tau _1-\xi _{1}^{2}|)^{b'} (1+|\tau -\tau _1+(\xi -\xi _1)^2|)^{b'} }.\nonumber \\ \end{aligned}$$

Next, we will consider the following two possible microlocalizations:

$\bullet$ **Microlocalization I.** ($|\xi _1|\le |\xi |$). It is defined by the region

5.26 $$\begin{aligned} E_{1} \doteq \big \{ (\xi _1,\tau _1,\xi ,\tau ) \in \mathbb {R}^4: |\xi _1|>1, \quad |\xi -\xi _1|>1 \quad \text {and} \quad |\xi _1|\le |\xi | \big \}. \end{aligned}$$

$\bullet$ **Microlocalization II. **($|\xi _1|> |\xi |$). It is defined by the region

5.27 $$\begin{aligned} E_{2} \doteq \big \{ (\xi _1,\tau _1,\xi ,\tau ) \in \mathbb {R}^4: |\xi _1|>1, \quad |\xi -\xi _1|>1 \quad \text {and} \quad |\xi _1|> |\xi | \big \}. \end{aligned}$$

**Proof in Microlocalization I.** ($1< |\xi _1|\le |\xi |$). Then, the $L^2$ bilinear estimate (5.23) reads

5.28 $$\begin{aligned} \Big \Vert \int _{\mathbb {R}^2} (\chi _{E_{1}}Q_1)(\xi _1,\tau _1,\xi ,\tau ) \cdot c_f(\xi -\xi _1,\tau -\tau _1) c_{g}(\xi _1,\tau _1) d\xi _1 d\tau _1 \Big \Vert _{L^2_{\xi ,\tau }} \lesssim \Vert c_f\Vert _{L^2_{\xi ,\tau }} \Vert c_{g}\Vert _{L^2_{\xi ,\tau }}. \end{aligned}$$

Using the Cauchy–Schwarz inequality with respect to $\xi _1,\tau _1$, we have

5.29 $$\begin{aligned} \text {LHS of }{(5.28)} \lesssim \Big \Vert \int _{\mathbb {R}^2} (\chi _{E_{1}} Q_1^2)(\xi _1,\tau _1,\xi ,\tau ) d\xi _1 d\tau _1 \Big \Vert _{L^{\infty }_{\xi ,\tau } }^{1/2} \cdot \Vert c_{f}\Vert _{L^2_{\xi ,\tau }} \Vert c_{g}\Vert _{L^2_{\xi ,\tau }}. \end{aligned}$$

Thus, to prove $L^2$ bilinear estimate (5.28), it suffices to show the following result.

#### Lemma 5.5

Let $m=2j\ge 2$ be an even number. If $-j+\frac{1}{2}<s<0$ and $\max \{\frac{1}{3},-\frac{1}{2m}s+\frac{1}{4}+\frac{1}{4m}\}\le b'<\frac{1}{2}$, then for $\xi ,\tau \in \mathbb {R}$, we have

5.30 $$\begin{aligned}&\Theta _{5}(\xi ,\tau ) \doteq \frac{1}{ (1+|\tau +\xi ^{m}|)^{2b'} } \int _{\mathbb {R}^2} \frac{ (1+|\xi |)^{2s} }{ |\xi _1(\xi -\xi _1)|^{2s} }\nonumber \\&\qquad \frac{ \chi _{E_{1}} (\xi _1,\tau _1,\xi ,\tau ) \, d\tau _1 d\xi _1 }{ (1+|\tau -\tau _1+(\xi -\xi _1)^m|)^{2b'} (1+|\tau _1-\xi _1^m|)^{2b'} } \, \lesssim 1. \end{aligned}$$

#### Proof of Lemma 5.5

For $(\xi _1,\tau _1,\xi ,\tau )$ in $E_1$, we have $1<|\xi _1|\le |\xi |$. This implies that $|\xi -\xi _1|\le |\xi |+|\xi _1|\lesssim |\xi |$, which gives us $\chi _{E_{1}} (\xi _1,\tau _1,\xi ,\tau ) \cdot \frac{ (1+|\xi |)^{2\,s} }{ |\xi _1(\xi -\xi _1)|^{2\,s} } \lesssim \frac{ \chi _{|\xi |>1}(\xi ) }{ |\xi _1|^{2\,s} } \lesssim \chi _{|\xi |>1}(\xi )\cdot |\xi |^{-2\,s},$ for $s<0.$ Thus, factoring out $\chi _{|\xi |>1}(\xi )\cdot |\xi |^{-2s}$, from (5.30) we get

5.31 $$\begin{aligned}&\Theta _{5}(\xi ,\tau ) \lesssim \frac{\chi _{|\xi |>1}(\xi )\cdot |\xi |^{-2s}}{ (1+|\tau +\xi ^{m}|)^{2b'} } \int _{\mathbb {R}^2}\nonumber \\&\qquad \frac{ \chi _{E_{1}} (\xi _1,\tau _1,\xi ,\tau ) }{ (1+|\tau -\tau _1+(\xi -\xi _1)^m|)^{2b'} (1+|\tau _1-\xi _1^m|)^{2b'} } \, d\tau _1 d\xi _1. \end{aligned}$$

For the $\tau _1$ integral in (5.31), applying calculus estimate (3.24) with $\ell =\ell '=b'>\frac{1}{4}$, $x=\tau _1$, $\alpha =\tau +(\xi -\xi _1)^m$, $\beta =\xi _1^m$, we get

5.32 $$\begin{aligned}&\Theta _{5}(\xi , \tau ) \lesssim \frac{\chi _{|\xi |>1}(\xi )\cdot |\xi |^{-2s}}{ (1+|\tau +\xi ^{m}|)^{2b'} } \int _{\mathbb {R}}\nonumber \\&\qquad \frac{ \chi _{E_{1}} (\xi _1,\tau _1,\xi ,\tau ) }{ (1+|\tau +(\xi -\xi _1)^2-\xi _1^m|)^{4b'-1} } d\xi _1 \le \frac{\chi _{|\xi |>1}(\xi )\cdot |\xi |^{-2s}}{ (1+|\tau +\xi ^{m}|)^{2b'} } I_3(\xi ,\tau ), \end{aligned}$$

where the integral $I_3$ is defined in (5.17), which is bounded in (5.19), that is $I_{3} \lesssim \frac{1}{|\xi |^{m-1}} \big [ (1+|\tau +\xi ^m|)^{2-4b'} + |\xi |^{m(2-4b')} \big ].$ Combining above inequalities, we obtain

$$\begin{aligned} \Theta _5(\xi ,\tau ) \lesssim \chi _{|\xi |>1}(\xi ) \cdot \frac{ |\xi |^{-2s-m+1} }{ (1+|\tau +\xi ^m|)^{6b'-2} } + \chi _{|\xi |>1}(\xi ) \cdot \frac{ |\xi |^{-2s+m+1-4mb'} }{ (1+|\tau +\xi ^m|)^{2b'} }. \end{aligned}$$

The first fraction $\chi _{|\xi |>1}(\xi ) \cdot \frac{ |\xi |^{-2\,s-m+1} }{ (1+|\tau +\xi ^m|)^{6b'-2} }$ is bound if $6b'-2\ge 0$ and $-2s-m+1\le 0$. It suffices to have $b' \ge \frac{1}{3},$ and $s \ge -\frac{1}{2}m+\frac{1}{2} = -j+\frac{1}{2}.$ And, the second fraction $\chi _{|\xi |>1}(\xi ) \cdot \frac{ |\xi |^{-2\,s+m+1-4mb'} }{ (1+|\tau +\xi ^m|)^{2b'} }$ is bound if $b'\ge 0$ and $-2\,s+m+1-4mb'\le 0$. It suffices to have $b' \ge -\frac{1}{2\,m}s+\frac{1}{4}+\frac{1}{4\,m}.$ Since $b'<\frac{1}{2}$, we need $-\frac{1}{2m}s+\frac{1}{4}+\frac{1}{4m}<\frac{1}{2}$, or

5.33 $$\begin{aligned} s > -\frac{1}{2}m+\frac{1}{2} = -j+\frac{1}{2}, \end{aligned}$$

which is the critical exponent $s_2$ for the nonlinearity $N_2$. This completes the proof of Lemma 5.5. $\square$

**Proof in microlocalization II** ($|\xi _1|> |\xi |$ and $|\xi _1|>1$). Then, $L^2$ bilinear estimate (5.23) reads

5.34 $$\begin{aligned} \Big \Vert \int _{\mathbb {R}^2} (\chi _{E_{2}}Q_1)(\xi _1,\tau _1,\xi ,\tau ) \cdot c_f(\xi -\xi _1,\tau -\tau _1) c_{g}(\xi _1,\tau _1) d\xi _1 d\tau _1 \Big \Vert _{L^2_{\xi ,\tau }} \lesssim \Vert c_f\Vert _{L^2_{\xi ,\tau }} \Vert c_{g}\Vert _{L^2_{\xi ,\tau }}. \end{aligned}$$

Using duality and the Cauchy–Schwarz inequality twice, we get

$$\begin{aligned}&\Big \Vert \int _{\mathbb {R}^2} (\chi _{E_{2}}Q_1) \cdot c_f(\xi -\xi _1,\tau -\tau _1) c_{g}(\xi _1,\tau _1) d\xi _1 d\tau _1 \Big \Vert _{L^2_{\xi ,\tau }}\\&\qquad \lesssim \Big \Vert \int _{\mathbb {R}^2} (\chi _{E_{2}} Q_1^2) d\xi d\tau \Big \Vert ^{\frac{1}{2}}_{L_{\xi _1,\tau _1}^\infty } \cdot \Vert c_f\Vert _{L^2_{\xi ,\tau }} \Vert c_{g}\Vert _{L^2_{\xi ,\tau }}. \end{aligned}$$

Thus, in order to prove $L^2$ bilinear estimate (5.23), it suffices to show the following result

#### Lemma 5.6

Let $m=2j\ge 2$ be an even number. If $-\frac{1}{2} j+\frac{1}{4}<s<0$ and $\max \{\frac{1}{3},-\frac{1}{m}s+\frac{1}{4}+\frac{1}{4m}\}\le b'<\frac{1}{2}$, then for $\xi ,\tau \in \mathbb {R}$, we have

5.35 $$\begin{aligned}&\Theta _{6} \doteq \frac{1}{ (1+|\tau _1-\xi _1^m|)^{2b'} } \int _{\mathbb {R}^2} \frac{ (1+|\xi |)^{2s} }{ |\xi _1(\xi -\xi _1)|^{2s} } \nonumber \\&\qquad \cdot \frac{ \chi _{E_{2}} (\xi _1,\tau _1,\xi ,\tau ) \, d\tau d\xi }{ (1+|\tau -\tau _1+(\xi -\xi _1)^m|)^{2b'} (1+|\tau +\xi ^m|)^{2b'} } \lesssim 1. \end{aligned}$$

#### Proof of Lemma 5.6

Again, due to the symmetry of $d_{2,m}(\xi ,\xi _1)$ with respect to $\xi$ and $\xi _1$, the proof of this lemma is similar to the proof of Lemma 5.5. Therefore, we omit the detailed proof here. $\square$

### Optimality of $f\bar{g}$ spatial bilinear estimates

We will prove the following result.

#### Lemma 5.7

If $s<-\frac{1}{2}j+\frac{1}{4}$, then bilinear estimate $\Vert f\bar{g}\Vert _{X^{s,-b}} \lesssim \Vert f\Vert _{X^{s,b}} \Vert g\Vert _{X^{s,b}}$ fails for any $b\in \mathbb {R}$.

#### Proof of Lemma 5.7

It suffices to prove that if $s< -\frac{1}{2}j+\frac{1}{4}$, then $L^2$ bilinear estimate (5.1) fails for any $b=b'\in \mathbb {R}$. To prove this, for fix $N\in \mathbb {Z}^+$ we define the $L^2$ functions $c_{f}, c_{g}$ as follows

5.36 $$\begin{aligned} c_{f}(\xi _2,\tau _2) = \chi _{A}(\xi _2,\tau _2) \in L^2_{\xi _2,\tau _2}, \qquad c_{g}(\xi _1,\tau _1) = \chi _{\tilde{A}^+}(\xi _1,\tau _1) \in L^2_{\xi _1,\tau _1}, \end{aligned}$$

where $\chi _{A}(\cdot )$ is the characteristic function of the set *A* defined by

5.37 $$\begin{aligned} A = \Big \{ (\xi _2,\tau _2) \in \mathbb {R}^2 : -N-\frac{10}{N^{m-1}} \le \xi _2 \le -N+\frac{10}{N^{m-1}}, \quad |\tau _2+\xi _2^m| \le 10^m(m+1)! \Big \}, \end{aligned}$$

and $\chi _{\tilde{A}^+}(\cdot )$ is the characteristic function of the set $\tilde{A}^+$ defined by

5.38 $$\begin{aligned} \tilde{A}^+ = \Big \{ (\xi _1,\tau _1) \in \mathbb {R}^2 : N-\frac{1}{N^{m-1}} \le \xi _1 \le N+\frac{1}{N^{m-1}}, \quad |\tau _1-\xi _1^m| \le 1 \Big \}. \end{aligned}$$

Furthermore, by a straightforward computation we get

5.39 $$\begin{aligned} \Vert c_{f}\Vert _{L^2(\mathbb {R}^2)}^2 = \Vert c_{g}\Vert _{L^2(\mathbb {R}^2)}^2 \simeq \frac{1}{N^{m-1}}. \end{aligned}$$

Next, we define the integrand function $\Theta (\xi ,\tau )$ in $L^2$ bilinear estimate (5.1)

5.40 $$\begin{aligned}&\Theta \doteq \frac{(1+|\xi |)^s}{(1+|\tau +\xi ^m|)^{b}} \int _{\mathbb {R}^2}\nonumber \\&\quad \frac{c_{f}(\xi -\xi _1,\tau -\tau _1) c_{g}(\xi _1,\tau _1) \,d\xi _1 d\tau _1 }{(1+|\xi -\xi _1|)^s(1+|\tau -\tau _1+(\xi -\xi _1)^m|)^{b}(1+|\xi _1|)^s(1+|\tau _1-\xi _1^m|)^{b}}, \end{aligned}$$

and estimate its $L^2$ norm. In fact, below we shall show that the following key estimate holds

5.41 $$\begin{aligned} \Vert \Theta \Vert _{L^2_{\xi ,\tau }} \gtrsim \frac{1}{N^{2s+\frac{3}{2}(m-1)}}. \end{aligned}$$

Now, using inequalities (5.39) and (5.41), if $L^2$ bilinear estimate (5.1) holds, then $\frac{1}{N^{2\,s+\frac{3}{2}(m-1)}} \lesssim \frac{1}{N^{m-1}}$ or $N^{-2\,s} \lesssim N^{\frac{1}{2}(m-1)}.$ Since $N\gg 1$, we must have $-2s\le \frac{1}{2}(m-1)$, that is $s \ge -\frac{1}{2}j+\frac{1}{4}.$ This completes the proof of Lemma 5.7. $\square$

**Proof of inequality** (5.41) By performing a straightforward computation, we can establish the following result: If $(\xi ,\tau )\in \Delta \doteq \Big \{ (\xi ,\tau )\in \mathbb {R}^2: -\frac{1}{N^{m-1}} \le \xi \le \frac{1}{N^{m-1}}, \quad |\tau | \le 1 \Big \}$ and $(\xi _1,\tau _1)\in \tilde{A}^+$, then $(\xi -\xi _1,\tau -\tau _1)\in A$. Furthermore, restricting to the regions $(\xi ,\tau )\in \Delta$ and $(\xi _1,\tau _1)\in \tilde{A}$, we can derive the following estimate for $\Theta$ (similar to inequality (3.88))

$$\begin{aligned} \Vert \Theta \Vert _{L^2} \ge&\Big \Vert \frac{\chi _{\Delta }(\xi ,\tau )\cdot (1+|\xi |)^s}{(1+|\tau +\xi ^m|)^{b}} \int _{\mathbb {R}^2} \\&\quad \frac{\chi _{\tilde{A}^+}(\xi _1,\tau _1) \cdot c_{f}(\xi -\xi _1,\tau -\tau _1) c_{g}(\xi _1,\tau _1)\,d\xi _1 d\tau _1}{(1+|\xi -\xi _1|)^s(1+|\tau -\tau _1+(\xi -\xi _1)^m|)^{b}(1+|\xi _1|)^s(1+|\tau _1-\xi _1^m|)^{b}} \Big \Vert _{L^2_{\xi ,\tau }} \\ \ge&\Big \Vert \chi _{\Delta }(\xi ,\tau )\cdot \int _{\mathbb {R}^2} \frac{\chi _{\tilde{A}^+}(\xi _1,\tau _1)}{N^{2s}} d\xi _1 d\tau _1 \Big \Vert _{L^2_{\xi ,\tau }}\\&\quad = \frac{1}{N^{2s}} \cdot \int _{\mathbb {R}^2} \chi _{\tilde{A}^+}(\xi _1,\tau _1) d\xi _1 d\tau _1 \cdot \big \Vert \chi _{\Delta } \big \Vert _{L^2_\xi L^2_{\tau }} \gtrsim \frac{1}{N^{2s+\frac{3}{2}(m-1)}}, \end{aligned}$$

which is the desired inequality (5.41). $\square$

## Bilinear estimates in temporal Bourgain spaces

In this section, we will prove the temporal bilinear estimates for all nonlinearities.

### $\bar{f}\bar{g}$ temporal bilinear estimate

Following the notation used in $\bar{f}\bar{g}$ spatial bilinear estimate (inequality (3.3)), we reduce the proof of the $\bar{f}\bar{g}$ temporal bilinear to the following $L^2$ inequality

6.1 $$\begin{aligned} \Big \Vert \int _{\mathbb {R}^2} Q(\xi _1,\tau _1,\xi ,\tau ) \cdot c_{f}(\xi -\xi _1,\tau -\tau _1) c_{g}(\xi _1,\tau _1) d\xi _1 d\tau _1 \Big \Vert _{L^2_{\xi ,\tau }} \lesssim \Vert c_{f}\Vert _{L^2_{\xi ,\tau }} \Vert c_{g}\Vert _{L^2_{\xi ,\tau }}, \end{aligned}$$

where the multiplier $Q=Q(\xi _1,\tau _1,\xi ,\tau )$ is defined as

6.2 $$\begin{aligned}&Q \doteq \frac{ (1+|\tau |)^{s/m} }{ (1+|\xi _1|)^{s} (1+|\xi -\xi _1|)^{s} } \cdot \frac{1}{(1+|\tau +\xi ^m|)^{b}} \nonumber \\&\quad \cdot \frac{1}{ (1+|\tau _1-\xi _{1}^{m}|)^{b'} (1+|\tau -\tau _1-(\xi -\xi _1)^m|)^{b'} }. \end{aligned}$$

By the symmetry of convolution writing, we assume that $|\tau -\tau _1-(\xi -\xi _1)^m| \le |\tau _1-\xi _1^m|.$ And, using $b'\le b$, the $L^2$ bilinear estimate (6.1), is reduced to the following $L^2$ bilinear estimate

6.3 $$\begin{aligned} \Big \Vert \int _{\mathbb {R}^2} \chi _{A} \cdot Q' \cdot c_{f}(\xi -\xi _1,\tau -\tau _1) c_{g}(\xi _1,\tau _1) d\xi _1 d\tau _1 \Big \Vert _{L^2_{\xi ,\tau }} \lesssim \Vert c_{f}\Vert _{L^2_{\xi ,\tau }} \Vert c_{g}\Vert _{L^2_{\xi ,\tau }}, \end{aligned}$$

where the region *A* is the region defined in (3.8) and the multiplier $Q'$ is defined as follows

6.4 $$\begin{aligned}&Q' \doteq \frac{ (1+|\tau |)^{s/m} }{ (1+|\xi _1|)^{s} (1+|\xi -\xi _1|)^{s} } \cdot \frac{1}{(1+|\tau +\xi ^m|)^{b'}}\nonumber \\&\quad \cdot \frac{1}{ (1+|\tau _1-\xi _{1}^{m}|)^{b'} (1+|\tau -\tau _1-(\xi -\xi _1)^m|)^{b'} }. \end{aligned}$$

Similar to the proof of the $\bar{f}\bar{g}$ spatial bilinear estimate, we consider the following two cases:

$$\bullet $$ Case: $$s\ge 0$$       $$\bullet $$ Case: $$s< 0$$.

**Bilinear estimate for** $s\ge 0$ If $|\tau |\le 10^m\max \{\xi ^m,\xi _1^m\}$, then $\frac{(1+|\tau |)^{s/m} }{(1+|\xi _1|)^{s}(1+|\xi -\xi _1|)^{s}}\lesssim 1$. This reduces $L^2$ bilinear estimate (6.3) to the $\bar{f}\bar{g}$ spatial $L^2$ bilinear estimate (3.9) with $s=0$. Hence, we assume that $|\tau |> 10^m\max \{\xi ^m,\xi _1^m\}$. Under this assumption, we get $|\tau +\xi ^m|\simeq |\tau |$. This implies that

$$\begin{aligned}  &   \frac{ (1+|\tau |)^{\frac{1}{m}s} }{ (1+|\xi _1|)^{s} (1+|\xi -\xi _1|)^{s} } \cdot \frac{1}{(1+|\tau +\xi ^m|)^{b'}} \lesssim \frac{(1+|\tau |)^{\frac{1}{m}s}}{(1+|\tau |)^{b'}} = \frac{1}{(1+|\tau |)^{b'-\frac{1}{m}s}},\\  &   \quad \text {if} \,\, |\tau |> 10^m\max \{\xi ^m,\xi _1^m\}. \end{aligned}$$

Therefore, in order to show temporal $L^2$ bilinear estimate (6.3) for $s\ge 0$, it suffices to show that

6.5 $$\begin{aligned} \Big \Vert \int _{\mathbb {R}^2} \chi _{A^+} \cdot Q_1 \cdot c_{f}(\xi -\xi _1,\tau -\tau _1) c_{g}(\xi _1,\tau _1) d\xi _1 d\tau _1 \Big \Vert _{L^2_{\xi ,\tau }} \lesssim \Vert c_{f}\Vert _{L^2_{\xi ,\tau }} \Vert c_{g}\Vert _{L^2_{\xi ,\tau }}, \end{aligned}$$

where the region $A^+\subset A$ is defined as follows

6.6 $$\begin{aligned}  &   A^+ \doteq \{ (\xi _1,\tau _1,\xi ,\tau )\in \mathbb {R}^4: |\tau -\tau _1-(\xi -\xi _1)^m|\nonumber \\  &   \quad \le |\tau _1-\xi _1^m| \quad \text {and} \quad |\tau | > 10^m\max \{\xi ^m,\xi _1^m\} \}, \end{aligned}$$

and the multiplier $Q_1$ is defined by

6.7 $$\begin{aligned} Q_1 \doteq \frac{1}{(1+|\tau |)^{b'-\frac{1}{m}s}} \cdot \frac{1}{ (1+|\tau _1-\xi _{1}^{m}|)^{b'} (1+|\tau -\tau _1-(\xi -\xi _1)^m|)^{b'} }. \end{aligned}$$

Next, applying Cauchy–Schwarz inequality in $\xi _1,\tau _1$ we get

6.8 $$\begin{aligned}&\Big \Vert \int _{\mathbb {R}^2} \chi _{A^+} \cdot Q_1 \cdot c_{f}(\xi -\xi _1,\tau -\tau _1) c_{g}(\xi _1,\tau _1) d\xi _1 d\tau _1 \Big \Vert _{L^2_{\xi ,\tau }}\nonumber \\&\quad \lesssim \Big \Vert \int _{\mathbb {R}^2} \chi _{A^+} Q_1^2 d\xi _1 d\tau _1 \Big \Vert _{L^{\infty }_{\xi ,\tau } }^{1/2} \Vert c_{f}\Vert _{L^2} \Vert c_{g}\Vert _{L^2}. \end{aligned}$$

Thus, to prove $L^2$ bilinear estimate (6.5), it suffices to show the following result.

#### Lemma 6.1

Let $m=2j\ge 2$ be an even number. If $0\le s<m-\frac{1}{2}$ and $\max \{\frac{1}{3}, \frac{1}{6}+\frac{2\,s+1}{6\,m}\}\le b'<\frac{1}{2}$, then for $\xi ,\tau \in \mathbb {R}$, we have

6.9 $$\begin{aligned}&\Theta _{1} (\xi , \tau ) \doteq \frac{1}{(1+|\tau |)^{2b'-\frac{2}{m}s}} \int _{\mathbb {R}^2}\nonumber \\&\quad \frac{ \chi _{A^+} (\xi _1,\tau _1,\xi ,\tau ) }{ (1+|\tau -\tau _1-(\xi -\xi _1)^m|)^{2b'} (1+|\tau _1-\xi _1^m|)^{2b'} } \, d\tau _1 d\xi _1 \lesssim 1. \end{aligned}$$

#### Proof of Lemma 6.1

For the $\tau _1$ integral in (3.23), applying calculus estimate (3.24) with $\ell =\ell '=b'>\frac{1}{4}$, $x=\tau _1$, $\alpha =\tau -(\xi -\xi _1)^m$, $\beta =\xi _1^m$, we get

6.10 $$\begin{aligned} \Theta _{1}(\xi , \tau )&\lesssim \frac{1}{(1+|\tau |)^{2b'-\frac{2}{m}s}} \int _{\mathbb {R}} \frac{ \chi _{A^+} (\xi _1,\tau _1,\xi ,\tau ) }{ (1+|\tau -(\xi -\xi _1)^m-\xi _1^m|)^{4b'-1} } d\xi _1\nonumber \\&= \frac{1}{(1+|\tau |)^{2b'-\frac{2}{m}s}} I_1(\xi ,\tau ), \end{aligned}$$

where the integral $I_1$ is defined as follows

6.11 $$\begin{aligned} I_1(\xi ,\tau ) \doteq \int _{\mathbb {R}} \frac{ \chi _{A^+} (\xi _1,\tau _1,\xi ,\tau ) }{ (1+|\tau -(\xi -\xi _1)^m-\xi _1^m|)^{4b'-1} } d\xi _1. \end{aligned}$$

Furthermore, later we will prove the following estimate for the integral $I_1$

6.12 $$\begin{aligned} I_1(\xi ,\tau ) \lesssim \frac{1}{(1+|\tau |)^{4b'-1-\frac{1}{m}}}. \end{aligned}$$

Next, combining inequality (6.12) with (6.10), we obtain

6.13 $$\begin{aligned} \Theta _{1}(\xi , \tau )&\lesssim \frac{1}{(1+|\tau |)^{2b'-\frac{2}{m}s}} \frac{1}{(1+|\tau |)^{4b'-1-\frac{1}{m}}} \lesssim \frac{1}{(1+|\tau |)^{6b'-\frac{2}{m}s-1-\frac{1}{m}}}\nonumber \\&= \frac{1}{(1+|\tau |)^{6b'-\frac{2s+1}{m}-1}}, \end{aligned}$$

which is bounded if $6b'-\frac{2\,s+1}{m}-1\ge 0$ or $b' \ge \frac{1}{6}+\frac{2\,s+1}{6\,m}.$ Since $b'<\frac{1}{2}$, we need $\frac{1}{6}+\frac{2s+1}{6m}<\frac{1}{2}$, or

6.14 $$\begin{aligned} s < m-1/2. \end{aligned}$$

This completes the proof of Lemma 6.1 once we prove inequality (6.12). $\square$

**Proof of inequality **(6.12) For $(\xi _1,\tau _1,\xi ,\tau )\in A^+$, we have $|\tau |>10^m\max \{\xi ^m,\xi _1^m\}$, which implies that

6.15 $$\begin{aligned} |\xi _1| \lesssim |\tau |^{\frac{1}{m}} \quad \text {and} \quad |\tau -(\xi -\xi _1)^m-\xi _1^m| \simeq |\tau |, \quad \text {if} \,\, (\xi _1,\tau _1,\xi ,\tau )\in A^+. \end{aligned}$$

In fact, since $|\tau |>10^m\max \{\xi ^m,\xi _1^m\}$, we get $|\xi _1| \lesssim |\tau |^{\frac{1}{m}}$ and $(\xi -\xi _1)^m \le [2\max \{|\xi |,|\xi _1|\}]^m = 2^m \max \{\xi ^m,\xi _1^m\} \le \frac{1}{5^m}|\tau |.$ This gives us that

$$\begin{aligned} \frac{1}{2}|\tau |  &   \le |\tau |-\frac{1}{5^m}-\frac{1}{10^m}\xi _1^m \le |\tau |-(\xi -\xi _1)^m-\xi _1^m \le |\tau -(\xi -\xi _1)^m-\xi _1^m|\\  &   \le |\tau |+(\xi -\xi _1)^m+\xi _1^m \le 3|\tau |, \end{aligned}$$

which implies relation (6.15). Using relation (6.15), we obtain

6.16 $$\begin{aligned} I_{1}&\lesssim \int _{\mathbb {R}} \frac{ \chi _{A^+} (\xi _1,\tau _1,\xi ,\tau ) }{ (1+|\tau |)^{4b'-1} } d\xi _1 = \frac{1}{(1+|\tau |)^{4b'-1}} \int _{A^+} 1 d\xi _1\nonumber \\&\lesssim \frac{1}{(1+|\tau |)^{4b'-1}} |\tau |^{\frac{1}{m}} \lesssim \frac{1}{(1+|\tau |)^{4b'-1-\frac{1}{m}}}, \end{aligned}$$

which is the desired inequality (6.12).    $\square$

**Bilinear estimate for** $s< 0$ Similar to the proof for case $s\ge 0$, if $|\tau |\ge \frac{1}{100} \xi ^m$, then the temporal $L^2$ bilinear estimate (6.3) is reduced to the spatial $L^2$ bilinear estimate (3.9). Therefore, in order to show temporal $L^2$ bilinear estimate (6.3) for $s< 0$, it suffices to show that

6.17 $$\begin{aligned} \Big \Vert \int _{\mathbb {R}^2} (\chi _{A^-}Q_2)(\xi _1,\tau _1,\xi ,\tau ) \cdot c_{f}(\xi -\xi _1,\tau - \tau _1) c_{g}(\xi _1,\tau _1) d\xi _1 d\tau _1 \Big \Vert _{L^2_{\xi ,\tau }} \lesssim \Vert c_{f}\Vert _{L^2_{\xi ,\tau }} \Vert c_{g}\Vert _{L^2_{\xi ,\tau }}, \end{aligned}$$

where the region $A^-\subset A$ is defined as follows

6.18 $$\begin{aligned} A^- \doteq \{ (\xi _1,\tau _1,\xi ,\tau )\in \mathbb {R}^4: |\tau -\tau _1-(\xi -\xi _1)^m| \le |\tau _1-\xi _1^m| \quad \text {and} \quad |\tau | < \frac{1}{100}\xi ^m \},\nonumber \\ \end{aligned}$$

and the multiplier $Q_2$ is defined by

6.19 $$\begin{aligned}&Q_2 \doteq \frac{(1+|\tau |)^{\frac{1}{m}s}}{(1+|\xi _1|)^{s} (1+|\xi -\xi _1|)^{s}} \frac{1}{(1+|\tau +\xi ^m|)^{b'}}\nonumber \\&\quad \cdot \frac{1}{ (1+|\tau _1-\xi _{1}^{m}|)^{b'} (1+|\tau -\tau _1-(\xi -\xi _1)^m|)^{b'} }. \end{aligned}$$

Furthermore, we consider the following two microlocalizations:

$\bullet$ **Microlocalization I.** It is defined by the region

6.20 $$\begin{aligned} A_{1} \doteq \left\{ (\xi _1,\tau _1,\xi ,\tau ) \in A^-: |\tau | < \frac{1}{100}\xi ^m \,\, \text {and} \,\, |\xi _1| \le 100(1+|\xi |) \right\} . \end{aligned}$$

$\bullet$ **Microlocalization II**. It is defined by the region

6.21 $$\begin{aligned} A_{2} \doteq \left\{ (\xi _1,\tau _1,\xi ,\tau ) \in A^-: |\tau | < \frac{1}{100}\xi ^m \,\, \text {and} \,\, |\xi _1| > 100(1+|\xi |) \right\} . \end{aligned}$$

**Proof in Microlocalization I.** Then, $L^2$ bilinear estimate (6.17) reads

6.22 $$\begin{aligned}&\Big \Vert \int _{\mathbb {R}^2} \chi _{A_{1}} (\xi _1,\tau _1,\xi ,\tau ) \cdot Q_2 (\xi _1,\tau _1,\xi ,\tau ) \cdot c_{f}(\xi -\xi _1,\tau -\tau _1) c_{g}(\xi _1,\tau _1) d\xi _1 d\tau _1 \Big \Vert _{L^2_{\xi ,\tau }}\nonumber \\&\quad \lesssim \Vert c_{f}\Vert _{L^2_{\xi ,\tau }} \Vert c_{g}\Vert _{L^2_{\xi ,\tau }}. \end{aligned}$$

Applying the Cauchy–Schwarz inequality in $\xi _1,\tau _1$, we have

6.23 $$\begin{aligned} \text {LHS of }{(6.22)} \lesssim \Big \Vert \int _{\mathbb {R}^2} \chi _{A_{1}}(\xi _1,\tau _1,\xi ,\tau ) \cdot Q_2^2(\xi _1,\tau _1,\xi ,\tau ) d\xi _1 d\tau _1 \Big \Vert _{L^{\infty }_{\xi ,\tau } }^{1/2} \cdot \Vert c_{f}\Vert _{L^2_{\xi ,\tau }} \Vert c_{g}\Vert _{L^2_{\xi ,\tau }}. \end{aligned}$$

Thus, to prove $L^2$ bilinear estimate (6.22), it suffices to show the following result.

#### Lemma 6.2

Let $m=2j\ge 2$ be an even number. If $-j+\frac{1}{4}<s<0$ and $\max \{\frac{1}{3}-\frac{s}{3m},\frac{1}{6}-\frac{4s-1}{6m}\}< b'<\frac{1}{2}$, then for $\xi ,\tau \in \mathbb {R}$, we have

$$\begin{aligned}&\Theta _{2} \doteq \frac{1}{ (1+|\tau +\xi ^m|)^{2b'} } \int _{\mathbb {R}^2} \frac{(1+|\tau |)^{\frac{2}{m}s}}{(1+|\xi _1|)^{2s} (1+|\xi -\xi _1|)^{2s}}\\&\quad \frac{ \chi _{A_{1}} (\xi _1,\tau _1,\xi ,\tau ) d\tau _1 d\xi _1 }{ (1+|\tau -\tau _1-(\xi -\xi _1)^m|)^{2b'} (1+|\tau _1-\xi _1^m|)^{2b'} } \lesssim 1. \end{aligned}$$

#### Proof of Lemma 6.2

For the $\tau _1$ integral in $\Theta _2$, applying calculus estimate (3.24) with $\ell =\ell '=b'>\frac{1}{4}$, $x=\tau _1$, $\alpha =\tau -(\xi -\xi _1)^m$, $\beta =\xi _1^m$, we get

6.24 $$\begin{aligned} \Theta _{2}(\xi , \tau ) \lesssim \frac{\chi _{|\tau | < \frac{1}{100}\xi ^m}(\xi ,\tau )}{ (1+|\tau +\xi ^m|)^{2b'} } I_2(\xi ,\tau ), \end{aligned}$$

where the integral $I_2$ is defined by

6.25 $$\begin{aligned} I_2(\xi ,\tau ) \doteq \int _{\mathbb {R}} \frac{ (1+|\tau |)^{\frac{2}{m}s} }{ (1+|\xi _1|)^{2s} (1+|\xi -\xi _1|)^{2s} } \frac{ \chi _{|\xi _1| \le 100(1+|\xi |)} (\xi _1) }{ (1+|\tau +\xi ^m-d_{3,m}(\xi ,\xi _1)|)^{4b'-1} } d\xi _1. \end{aligned}$$

Here, $d_{3,m}(\xi ,\xi _1)=\xi ^m+\xi _1^m+(\xi -\xi _1)^m$. For $|\tau |<\frac{1}{100}\xi ^m$, we have $|\tau +\xi ^m| \simeq \xi ^m$, which implies that

6.26 $$\begin{aligned} \frac{\chi _{|\tau |<\frac{1}{100}\xi ^m}(\xi ,\tau )}{(1+|\tau +\xi ^m|)^{2b'}} \simeq \frac{1}{(1+\xi ^m)^{2b'}}\simeq \frac{1}{(1+|\xi |)^{2mb'}}. \end{aligned}$$

Similar to the proof of inequality (3.56), we can establish the following bound for the integral $I_2$:

6.27 $$\begin{aligned}&(\chi _{|\tau |<\frac{1}{100}\xi ^m} \cdot I_2) (\xi ,\tau ) \lesssim \frac{1}{(1+|\xi |)^{2s+m(4b'-2)}} + \frac{1}{(1+|\xi |)^{4s+4mb'-m-1}}, \nonumber \\&\quad s<0 \,\, \text {and} \,\, \frac{1}{4}\le b'<\frac{1}{2}. \end{aligned}$$

Next, combining estimate (6.27) with inequalities (6.24), (6.26), we obtain

6.28 $$\begin{aligned} \Theta _{2}(\xi ,\tau ) \lesssim \frac{1}{(1+|\xi |)^{6mb'-2m+2s}} + \frac{1}{(1+|\xi |)^{6mb'-m+4s-1}}. \end{aligned}$$

The first fraction $\frac{1}{(1+|\xi |)^{6mb'-2m+2s}}$ is bounded if $6mb'-2\,m+2\,s\ge 0$ or $b' \ge \frac{1}{3}-\frac{s}{3\,m}.$ Since $b'<\frac{1}{2}$, it suffices to have $-\frac{s}{3m}<\frac{1}{6}$ or $s>-\frac{1}{2}m=-j$. And the second fraction $\frac{1}{(1+|\xi |)^{6mb'-m+4s-1}}$ is bounded if $6mb'-m+4s-1\ge 0$ or $b' \ge \frac{1}{6}-\frac{4\,s-1}{6\,m}.$ Since $b'<\frac{1}{2}$, it suffices to have $\frac{1}{6}-\frac{4s-1}{6m}<\frac{1}{2}$, or $s>-j+\frac{1}{4}.$ This completes the proof of Lemma 6.2. $\square$

**Proof in Microlocalization II** Then, $L^2$ bilinear estimate (6.17) reads

6.29 $$\begin{aligned}&\Big \Vert \int _{\mathbb {R}^2} \chi _{A_{2}} (\xi _1,\tau _1,\xi ,\tau ) \cdot Q_2 (\xi _1,\tau _1,\xi ,\tau ) \cdot c_{f}(\xi -\xi _1,\tau -\tau _1) c_{g}(\xi _1,\tau _1) d\xi _1 d\tau _1 \Big \Vert _{L^2_{\xi ,\tau }} \nonumber \\&\quad \lesssim \Vert c_{f}\Vert _{L^2_{\xi ,\tau }} \Vert c_{g}\Vert _{L^2_{\xi ,\tau }}, \end{aligned}$$

Using duality and the Cauchy–Schwarz inequality twice, we have

$$\begin{aligned}&\Big \Vert \int _{\mathbb {R}^2} (\chi _{A_2}Q_2) \cdot c_{f}(\xi -\xi _1,\tau -\tau _1) c_{g}(\xi _1,\tau _1) d\xi _1 d\tau _1 \Big \Vert _{L^2_{\xi ,\tau }} \\&\quad \lesssim \Big \Vert \int _{\mathbb {R}^2} \chi _{A_2}\cdot Q_2 d\xi d\tau \Big \Vert _{L^{\infty }_{\xi _1,\tau _1} }^{1/2} \Vert c_{f}\Vert _{L^2_{\xi ,\tau }} \Vert c_{g}\Vert _{L^2_{\xi ,\tau }}. \end{aligned}$$

Thus, in this situation to prove $L^2$ bilinear estimate (6.17), it suffices to show the following result.

#### Lemma 6.3

Let $m=2j\ge 2$ be an even number. If $-j+\frac{1}{4}<s<0$ and $\max \{\frac{1}{3},\frac{1}{6}-\frac{4\,s-1}{6\,m}\}\le b' <\frac{1}{2}$, then for $\xi _1,\tau _1\in \mathbb {R}$, we have

$$\begin{aligned} \Theta _{3} \doteq&\frac{1}{ (1+|\tau _1-\xi _1^{m}|)^{2b'} } \int _{\mathbb {R}^2} \frac{(1+|\tau |)^{\frac{2}{m}s}}{(1+|\xi _1|)^{2s} (1+|\xi -\xi _1|)^{2s}}\\&\frac{ \chi _{A_{2}} (\xi _1,\tau _1,\xi ,\tau ) d\tau d\xi }{ (1+|\tau -\tau _1-(\xi -\xi _1)^m|)^{2b'} (1+|\tau +\xi ^m|)^{2b'} } \lesssim 1. \end{aligned}$$

#### Proof of Lemma 6.3

For $s<0$, we have $(1+|\tau |)^{2s/m}<1$, this gives us that

$$\begin{aligned} \Theta _{3}  &   \lesssim \frac{1}{ (1+|\tau _1-\xi _1^{m}|)^{2b'} } \int _{\mathbb {R}^2} \frac{1}{(1+|\xi _1|)^{2s} (1+|\xi -\xi _1|)^{2s}}\\  &   \frac{ \chi _{A_{2}} (\xi _1,\tau _1,\xi ,\tau ) }{ (1+|\tau -\tau _1-(\xi -\xi _1)^m|)^{2b'} (1+|\tau +\xi ^m|)^{2b'} } \, d\tau d\xi . \end{aligned}$$

For the $\tau$ integral in the above inequality, applying calculus estimate (3.24) with $\ell =\ell '=b'>\frac{1}{4}$, $x=\tau _1$, $\alpha =\tau _1+(\xi -\xi _1)^m$, $\beta =-\xi ^m$, we get

6.30 $$\begin{aligned} \Theta _{3} \lesssim \frac{1}{ (1+|\tau _1-\xi _1^{m}|)^{2b'} } I_3(\xi _1,\tau _1), \end{aligned}$$

where the integral $I_3(\xi _1,\tau _1)$ is defined by

6.31 $$\begin{aligned} I_3(\xi _1,\tau _1) \doteq \int _{\mathbb {R}} \frac{1}{(1+|\xi _1|)^{2s} (1+|\xi -\xi _1|)^{2s}} \frac{ \chi _{A_{2}} (\xi _1,\tau _1,\xi ,\tau ) }{ (1+|\tau _1-\xi _1^m+d_{3,m}(\xi ,\xi _1)|)^{4b'-1} } d\xi , \end{aligned}$$

with $d_{3,m}(\xi ,\xi _1)=\xi ^m+\xi _1^m+(\xi -\xi _1)^m$. Also, working similarly to inequality (3.77), we can establish the following bound for the integral $I_3$.

6.32 $$\begin{aligned} I_3(\xi _1,\tau _1) \lesssim \frac{1}{(1+|\tau _1-\xi _1^m|)^{4b'-2+(4s+m-1)/m}}, \quad s<0 \,\, \text {and} \,\, b'<\frac{1}{2}. \end{aligned}$$

Next, combining estimate (6.32) with inequality (6.30), we get

$$\begin{aligned}&\Theta _{3}(\xi _1, \tau _1) \lesssim \frac{1}{(1+|\tau _1-\xi _1^m|)^{2b'}} \frac{1}{(1+|\tau _1-\xi _1^m|)^{4b'-2+(4s+m-1)/m}}\\&\quad = \frac{1}{(1+|\tau _1-\xi _1^m|)^{6b'-2+(4s+m-1)/m}}, \end{aligned}$$

which is bounded if $6b'-2+(4\,s+m-1)/m\ge 0$ or $b' \ge \frac{1}{6}-\frac{4\,s-1}{6\,m}.$ Since $b'<\frac{1}{2}$, it suffices to have $s>-j+\frac{1}{4}.$ This completes the proof of Lemma 6.3. $\square$

### *fg* temporal bilinear estimate

The *fg* temporal bilinear estimate: $\Vert fg\Vert _{Y^{s,-b}} \lesssim \Vert f\Vert _{X^{s,b}} \Vert g\Vert _{X^{s,b}},$ follows from *fg* spatial bilinear estimate and the following result

#### Lemma 6.4

Let $m=2j\ge 2$ be an even number. If $h\in X^{s,b}(\mathbb {R}^{2})$ and $\bar{h}\in Y^{s,b}(\mathbb {R}^{2})$, then we have

6.33 $$\begin{aligned} \Vert h\Vert _{Y^{s,b}(\mathbb {R}^{2})} \lesssim \Vert h\Vert _{X^{s,b}(\mathbb {R}^{2})} + \Vert \bar{h}\Vert _{Y^{s,b}(\mathbb {R}^{2})}, \quad \forall s,b\in \mathbb {R}, \end{aligned}$$

where the spatial Bourgain spaces $X^{s,b}(\mathbb {R}^{2})$ and the temporal Bourgain spaces $Y^{s,b}(\mathbb {R}^{2})$ are defined in (1.12) and (1.16), respectively.

In fact, applying inequality (6.33) with $h=fg$, we get $\Vert fg\Vert _{Y^{s,-b}} \lesssim \Vert fg\Vert _{X^{s,-b}} + \Vert \bar{f}\bar{g}\Vert _{Y^{s,-b}}.$ Then, applying *fg* spatial bilinear estimate and $\bar{f}\bar{g}$ temporal bilinear estimate, we get the *fg* temporal bilinear estimate (1.24). $\square$

#### Proof of Lemma 6.4

First, we split the $Y^{s,b}$ norm as $\Vert h\Vert _{Y^{s,b}(\mathbb {R}^{2})}^2 = I_1+I_2,$ where the integrals $I_1$ and $I_2$ are defined as follows

6.34 $$\begin{aligned}&I_1 \doteq \int _{\mathbb {R}^2} \chi _{A}(\xi ,\tau ) \cdot (1+|\tau |)^{\frac{2}{m}s} (1+|\tau +\xi ^m|)^{2b} |\widehat{h}(\xi ,\tau )|^2 d\xi d\tau , \end{aligned}$$

6.35 $$\begin{aligned}&I_2 \doteq \int _{\mathbb {R}^2} \chi _{A^c}(\xi ,\tau ) \cdot (1+|\tau |)^{\frac{2}{m}s} (1+|\tau +\xi ^m|)^{2b} |\widehat{h}(\xi ,\tau )|^2 d\xi d\tau . \end{aligned}$$

Here, the region *A* is defined by $A \doteq \{ (\xi ,\tau )\in \mathbb {R}^2: \frac{1}{10}|\tau |^{\frac{1}{m}} \le |\xi | \le 10|\tau |^{\frac{1}{m}} \}.$ Since $|\tau |^{\frac{1}{m}}\simeq |\xi |$, when $(\xi ,\tau )\in A$, we get

6.36 $$\begin{aligned} I_1 \lesssim \int _{\mathbb {R}^2} (1+|\xi |)^{2s} (1+|\tau +\xi ^m|)^{2b} |\widehat{h}(\xi ,\tau )|^2 d\xi d\tau = \Vert h\Vert _{X^{s,b}(\mathbb {R}^2)}^2, \quad s,b\in \mathbb {R}. \nonumber \\ \end{aligned}$$

Concerning $I_2$, using the elementary fact $\widehat{h}(\xi ,\tau ) = \widehat{\bar{h}}(-\xi ,-\tau ),$ we get $I_2 = \int _{\mathbb {R}^2} \chi _{A^c}(\xi ,\tau ) \cdot (1+|\tau |)^{\frac{2}{m}s} (1+|\tau +\xi ^m|)^{2b} |\widehat{\bar{h}}(-\xi ,-\tau )|^2 d\xi d\tau .$ Furthermore, making the change of variables $\xi \mapsto -\xi$, $\tau \mapsto -\tau$, we obtain $I_2 = \int _{\mathbb {R}^2} \chi _{A^c}(\xi ,\tau ) \cdot (1+|\tau |)^{\frac{2}{m}s} (1+|\tau -\xi ^m|)^{2b} |\widehat{\bar{h}}(\xi ,\tau )|^2 d\xi d\tau .$ Moreover, using $|\tau -\xi ^m| \simeq |\tau +\xi ^m|,$ when $(\xi ,\tau )\in A^c,$ we get

6.37 $$\begin{aligned} I_2&\simeq \int _{\mathbb {R}^2} \chi _{A^c}(\xi ,\tau ) \cdot (1+|\tau |)^{\frac{2}{m}s} (1+|\tau +\xi ^m|)^{2b} |\widehat{\bar{h}}(\xi ,\tau )|^2 d\xi d\tau \lesssim \Vert \bar{h}\Vert _{Y^{s,b}(\mathbb {R}^2)}^2, \nonumber \\&\quad \ \forall \, s,b\in \mathbb {R}. \end{aligned}$$

Combining inequalities (6.36) and (6.37), we complete the proof of Lemma 6.4. $\square$

### $f\bar{g}$ temporal bilinear estimate

Working like in the case of $\bar{f}\bar{g}$ temporal bilinear estimate, we reduce a part of the proof to that of $f\bar{g}$ spatial bilinear estimate. Another part of the proof is similar to the proof of the $f\bar{g}$ spatial bilinear estimate. Here, we omit the details.

## Proof of Well-posedness Theorem

Here, we outline the proof of our main result, that is Theorem  1.3. For initial data $u_0\in H^s(\mathbb {R})$, boundary data $g_\ell$ in $H_t^\frac{2s+m-1-2\ell }{2m}(0,T)$, and for $T^*$ such that $0<T^*\le T<1/2,$ we replace the iteration map (1.11) for our QNLSm ibvp (1.1), defined by the Fokas solution formula (1.6)

7.1 $$\begin{aligned}&u(x, t) = \Phi _{T^*} u \doteq S \Big [ u_0,g_0,\dots ,g_{j-1}; -\psi _{2T^*} N_k(u,\bar{u}) \Big ], \nonumber \\&\quad (x, t) \in \mathbb {R}^+ \times (0, T),\, \, T<1/2, \end{aligned}$$

where $$\psi _{T^*}(t)=\psi (t/{T^*})$$. We shall choose appropriate $$ {T^*}$$ and use the contraction mapping theorem to show that there is a fixed point of the iteration map (7.1) in the ball

7.2 $$\begin{aligned} B(r) \doteq \Big \{ u: u\in X^{s,b}_{\mathbb {R}^+\times (0,T)} \quad \text {and} \quad \Vert u\Vert _{X^{s,b}_{\mathbb {R}^+\times (0,T)}} \le r\Big \} \subseteq X^{s,b}_{\mathbb {R}^+\times (0,T)}. \end{aligned}$$

$\Phi _{T^*}$**is onto.** Using linear estimates (1.20) with forcing replaced by $-N_k(u,\bar{u})$, and applying bilinear estimates (1.21), (1.24) we get

7.3 $$\begin{aligned} \Vert \Phi _ {T^*}(u)\Vert _{X^{s,b}_{\mathbb {R}^+\times (0,T)}} \le c_1 \Big ( \Vert u_0 \Vert _{H^s(\mathbb {R}^+)} + \sum \limits _{\ell =0}^{j-1}\Vert g_\ell \Vert _{H_t^\frac{2s+m-1-2\ell }{2m}(0,T)} + 2c_2 \big \Vert \psi _{2{T^*}}(\cdot ) \tilde{u} \big \Vert _{X^{s,b'}} \big \Vert \tilde{u} \big \Vert _{X^{s,b'}} \Big ), \end{aligned}$$

where $\tilde{u}$ is the extension of *u* from $\mathbb {R}^+\times (0,T)$ to $\mathbb {R}^2$, which satisfies $\Vert {\tilde{u}}\Vert _{X^{s,b}} \le 2 \Vert u\Vert _{X^{s,b}_{\mathbb {R}^+\times (0,T)}}.$ At this point, we need the following multipliers result, whose proof can be found in [56].

### Lemma 7.1

Let $\eta (t)$ be a function in the Schwartz space $\mathcal {S}(\mathbb {R})$. If $-\frac{1}{2}< b' \le b < \frac{1}{2},$ then for any $0<{T^*}\le 1$ we have

7.4 $$\begin{aligned}&\Vert \eta (t/{T^*})u\Vert _{X^{s,b'}} \le c_3(\eta ,b',b) \,\, {T^*}^{b-b'}\Vert u\Vert _{X^{s,b}}. \end{aligned}$$

Next, applying inequality (7.4) with: $b = \frac{1}{2}-\frac{1}{2}\beta _k$ and $b' = \frac{1}{2}-\beta _k,$ where $\beta _k$ is defined in (1.25), we get $\Vert \psi _{2{T^*}} \tilde{u} \Vert _{X^{s,b'}} \le c_3 {T^*}^{\beta _k/2} \big \Vert \tilde{u} \big \Vert _{X^{s,b}}.$ Combining this with inequality (7.3) gives

7.5 $$\begin{aligned} \Vert \Phi _ {T^*}(u)\Vert _{X^{s,b}_{\mathbb {R}^+\times (0,T)}} \le c_4 \Big ( \Vert u_0 \Vert _{H^{s}(\mathbb {R}^+)} + \sum \limits _{\ell =0}^{j-1}\Vert g_\ell \Vert _{H_t^\frac{2s+m-1-2\ell }{2m}(0,T)} + {T^*}^{\beta _k/2} \big \Vert u \big \Vert _{X^{s,b}_{\mathbb {R}^+\times (0,T)}}^2 \Big ), \end{aligned}$$

where $c_4\doteq c_1+8c_1c_2c_3$. Finally, using inequality (7.5), we see that $\Phi _{T^*}$ is onto *B*(*r*), if we have

7.6 $$\begin{aligned}&c_4 \Big ( \Vert u_0 \Vert _{H^{s}(\mathbb {R}^+)} + \sum \limits _{\ell =0}^{j-1}\Vert g_\ell \Vert _{H_t^\frac{2s+m-1-2\ell }{2m}(0,T)} \Big ) + c_4 {T^*}^{\beta _k/2} r^2 \le r. \end{aligned}$$

$\Phi _{T^*}$**is contraction.** Working like in the onto case, for $u,v\in B(r)$ we apply the linear estimate (1.20) with the forcing term $N_k(u,\bar{u})-N_k(v,\bar{v})$, utilizing appropriate extensions of *u* and *v*. Then, we use the bilinear estimates (1.21) and (1.24) along with the multiplier estimate (7.4) and the same choices of *b* and $b'$ as in the onto case. Thus, we obtain the following inequality

7.7 $$\begin{aligned} \Vert \Phi _{T^*}(u)-\Phi _{T^*}(v)\Vert _{X^{s,b}_{\mathbb {R}^+\times (0,T)}} \le 4c_4r {T^*}^{\beta _k/2} \Vert u-v \Vert _{X^{s,b}_{\mathbb {R}^+\times (0,T)}}. \end{aligned}$$

Finally, combining (7.6), (7.7), and choosing *r* as: $r = 2c_4 \Big ( \Vert u_0 \Vert _{H^{s}(\mathbb {R}^+)} + \sum \nolimits _{\ell =0}^{j-1}\Vert g_\ell \Vert _{H_t^\frac{2\,s+m-1-2\ell }{2\,m}(0,T)} \Big ),$ we see that $\Phi _{T^*}$ is a contraction, if $T^*$ satisfies $T^* \lesssim \Big [ \Vert u_0 \Vert _{H^{s}(\mathbb {R}^+)} + \sum \nolimits _{\ell =0}^{j-1}\Vert g_\ell \Vert _{H_t^\frac{2\,s+m-1-2\ell }{2\,m}(0,T)} +1 \Big ]^{-2/\beta _k},$ which holds if we choose a lifespan $T^*=T_0<\frac{1}{2}$ that satisfies the estimate (1.30) stated in our well-posedness Theorem 1.3. The proofs for the Lip-continuity of the data-to-solution map and of the uniqueness of solution are similar to the ones presented in [2] for the well-posedness on the whole line. $\square$

## Fokas solution formula

Finally, we outline the derivation of the Fokas UTM solution formula following [22]. This derivation is similar to the one for a higher dispersion KdV presented in [34].

**Step 1: Solving ibvp **(1.5) **via half-line Fourier transform** For our derivation, we use the transpose of the homogeneous linear QNLSm, that is

8.1 $$\begin{aligned} -i\partial _t \tilde{u} + (-1)^{j+1} \partial ^{m}_x \tilde{u} = 0. \end{aligned}$$

Multiplying (8.1) by $-u$ and equation (1.5a) by $\tilde{u}$, and adding the resulting equations gives

8.2 $$\begin{aligned} ({\tilde{u}}u)_t + (-1)^{j}i \cdot [\tilde{u}\partial _x^{2j-1} u +\cdots +(-1)^{k}\partial _x^k\tilde{u}\partial _x^{2j-k-1}u+\cdots -\partial _x^{2j-1}{\tilde{u}}u]_x = -i {\tilde{u}}f.\nonumber \\ \end{aligned}$$

Then, we use the following exponential solutions to equation (8.1)

8.3 $$\begin{aligned} {\tilde{u}} = e^{-i\xi x+i\xi ^{m}t}, \quad \xi \in \mathbb {C}. \end{aligned}$$

Substituting (8.3) into (8.2) gives the divergence form

8.4 $$\begin{aligned}&\Big ( e^{-i\xi x+i\xi ^{m}t} u \Big )_t +(-1)^ji \nonumber \\&\cdot \Big ( e^{-i\xi x+i\xi ^{m}t} \big [ \partial _x^{2j-1} u+ \cdots +(i\xi )^k \partial _x^{2j-k-1} u + \cdots + (i\xi )^{2j-1} u \big ] \Big )_x \nonumber \\ =&-ie^{-i\xi x+i\xi ^mt} f(x, t). \end{aligned}$$

Integrating the divergence form (8.4) from $x=0$ to $\infty$ (assuming appropriate decay at $\infty$) gives

8.5 $$\begin{aligned} (e^{i\xi ^mt}{\widehat{u}}(\xi ,t))_t = -i e^{i\xi ^mt}{\widehat{f}}(\xi ,t)+(-1)^{j}i\cdot e^{i\xi ^mt}g(\xi ,t), \end{aligned}$$

where ${\widehat{u}}$ and ${\widehat{f}}$ are the half-line Fourier transforms of *u* and *f*, which are defined in (1.8), and *g* is the following combination of $m=2j$ boundary data (some of which are not given)

8.6 $$\begin{aligned} g(\xi ,t) \doteq \partial _x^{2j-1}u(0,t)+\cdots +(i\xi )^k\partial _x^{2j-k-1}u(0,t)+\dots +(i\xi )^{2j-1}u(0,t). \end{aligned}$$

Integrating (8.5) from 0 to *t*, $0\le t\le T$, we find the following global relation

8.7 $$\begin{aligned} e^{i\xi ^mt}{\widehat{u}}(\xi ,t)&= {\widehat{u}}_0(\xi ) -i F(\xi ,t) +(-1)^{j}i\cdot [{\tilde{g}}_{2j-1}(\xi ^m,t)+\cdots +(i\xi )^k\tilde{g}_{2j-k-1}(\xi ^m,t)\nonumber \\&\quad +\cdots +(i\xi )^{2j-1}{\tilde{g}}_0(\xi ^m,t)] , \quad \text {Im}(\xi )\le 0, \end{aligned}$$

where $F(\xi ,t)$ and ${\tilde{g}}_{\ell }(\xi ,t)$ are given in (1.9) and (1.10), respectively. Now, inverting (8.7) we get

8.8 $$\begin{aligned} u(x,t)&= \frac{1}{2\pi }\int _{-\infty }^\infty e^{i\xi x-i\xi ^mt} [{\widehat{u}}_0(\xi )-iF(\xi ,t)]d\xi \nonumber \\&\quad + \frac{(-1)^{j}i}{2\pi }\int _{-\infty }^\infty e^{i\xi x-i\xi ^mt} [{\tilde{g}}_{2j-1}(\xi ^m,t)+\cdots +(i\xi )^k\tilde{g}_{2j-k-1}(\xi ^m,t) \nonumber \\&\quad \ +\cdots +(i\xi )^{2j-1}{\tilde{g}}_0(\xi ^m,t)]d\xi . \end{aligned}$$

**Step 2: Deforming in the complex plane** Next, we provide another expression for the solution via a deformation in the complex plane, which allows us to eliminate the unknown data in formula (8.8).

### Lemma 8.1

The solution *u* to the linear QNLSm ibvp (1.5) can be expressed in the following form

8.9 $$\begin{aligned} u(x,t)&= \frac{1}{2\pi }\int _{-\infty }^\infty e^{i\xi x-i\xi ^mt} [{\widehat{u}}_0(\xi )-iF(\xi ,t)]d\xi \nonumber \\&+ \frac{(-1)^{j}i}{2\pi } \sum \limits _{p=1}^{j} \int _{\partial D_{2p-1}^+} e^{i\xi x-i\xi ^mt} [{\tilde{g}}_{2j-1}(\xi ^m,t)+\cdots +(i\xi )^k\tilde{g}_{2j-k-1}(\xi ^m,t)\nonumber \\&+\cdots +(i\xi )^{2j-1}{\tilde{g}}_0(\xi ^m,t)]d\xi , \end{aligned}$$

where the domains $D^+_1, \ldots , D_{2j-1}^+$ are displayed in Fig. 1 (if *j* is odd) or Fig. 2 (if *j* is even), and the orientation of the boundaries $\partial D^+_{2p-1}$, $p=1,2,\ldots ,j$, is given by the left-hand rule.

The proof of Lemma 8.1 is similar to that of the KdVm equation (see Lemma 7.1 in [34]).

**Step 3: Eliminating the unknown data** Since our equation has dispersion of order *m* (even), to eliminate the unknown data we need $j=\frac{m}{2}$ boundary conditions. In fact, for general dispersive equations, whether *m* is even or odd, the number of boundary conditions is provided in [22] (equation (1.5)). Now, we construct a linear system with *j* equations for each $p=1,2,\ldots ,j$. This is achieved by applying the invariant transformations of $\xi ^m$, specifically $\xi \mapsto \alpha _{p,n}\xi$, $n=1,2,\ldots ,j$, where $\alpha _{p,n}$ are the rotation numbers defined in (1.7). We also utilize the global relation (8.7). This process yields a linear system with the following *j* equations:

$$\begin{aligned} {\left\{ \begin{array}{ll} e^{i\xi ^mt}{\widehat{u}}(\alpha _{p,1}\xi ,t) = \widehat{u}_0(\alpha _{p,1}\xi ) -i F(\alpha _{p,1}\xi ,t) \\ +(-1)^{j}i\cdot [\tilde{g}_{2j-1}(\xi ^m,t)+\cdots +(i\alpha _{p,1}\xi )^k\tilde{g}_{2j-k-1}(\xi ^m,t) +\cdots +(i\alpha _{p,1}\xi )^{2j-1}\tilde{g}_0(\xi ^m,t)], \\ \cdots \\ e^{i\xi ^mt}{\widehat{u}}(\alpha _{p,n}\xi ,t) = \widehat{u}_0(\alpha _{p,n}\xi ) -i F(\alpha _{p,n}\xi ,t) \\ +(-1)^{j}i\cdot [\tilde{g}_{2j-1}(\xi ^m,t)+\cdots +(i\alpha _{p,n}\xi )^k\tilde{g}_{2j-k-1}(\xi ^m,t) +\cdots +(i\alpha _{p,n}\xi )^{2j-1}\tilde{g}_0(\xi ^m,t)], \\ \cdots \end{array}\right. } \end{aligned}$$

By solving these equations for $(i\xi )^k{\tilde{g}}_{2j-k-1}(\xi ^m,t)$, where $k=0,1,\ldots ,j-1$, and substituting the obtained solutions into formula (8.9), we derive the desired Fokas solution formula (1.6). Observe that this formula involves only the given data and does not depend on any unknown data.

## Declarations

### Availability of data and materials.

Data sharing is not applicable to this article as no datasets were used in this study.
